# Catalytic Hydrophosphorylation of Propiolates and Three-Component Phosphorylation of Aldehydes

**DOI:** 10.1021/acs.joc.5c02398

**Published:** 2026-01-21

**Authors:** Samuel Delgado-Hernández, Alejandro Peixoto de Abreu Lima, Eva M. Martín-Díaz, Jimena Scoccia, Romen Carrillo, David Tejedor

**Affiliations:** † 83098 Instituto de Productos Naturales Y Agrobiología, Consejo Superior de Investigaciones Científicas, Avda. Astrofísico Francisco Sánchez 3, Tenerife, La Laguna, Islas Canarias 38 206, Spain; ‡ Departamento de Química Orgánica, Facultad de Química, 201894 Universidad de la República, Av. General Flores 2124, Montevideo 11800, Uruguay; § Instituto Pasteur de Montevideo, Mataojo 2020, Montevideo 11400, Uruguay

## Abstract

A practical and efficient regio- and stereoselective hydrophosphorylation of propiolates, as well as a multicomponent reaction incorporating an aldehyde component, is reported. Both processes proceed with atom economy in very straightforward experimental procedures. The reactions are catalyzed by DABCO (1,4-diazabicyclo[2.2.2]­octane) and use readily available H-phosphonates as the phosphorylating agent.

## Graphical abstract

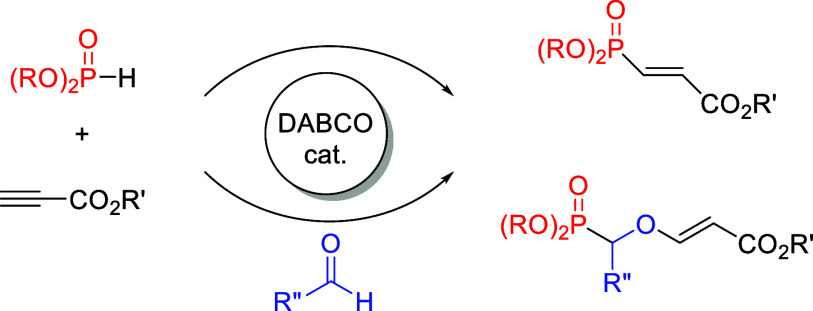

## Introduction

Organophosphorus compounds play an important role in many different areas of organic chemistry. Among them, those containing a phosphoryl P­(O) group have found many applications in medicinal, agricultural, or material chemistry.^1^ To construct the P–C bond, the most straightforward strategy has been the addition of H-phosphonates across unsaturated systems, exploiting the high reactivity of the P–H bond. The most relevant examples are the hydrophosphorylation of alkynes^2^ and the phospha–aldol reaction of carbonyl compounds^3^ (Scheme 1a–b).

**Scheme 1:**
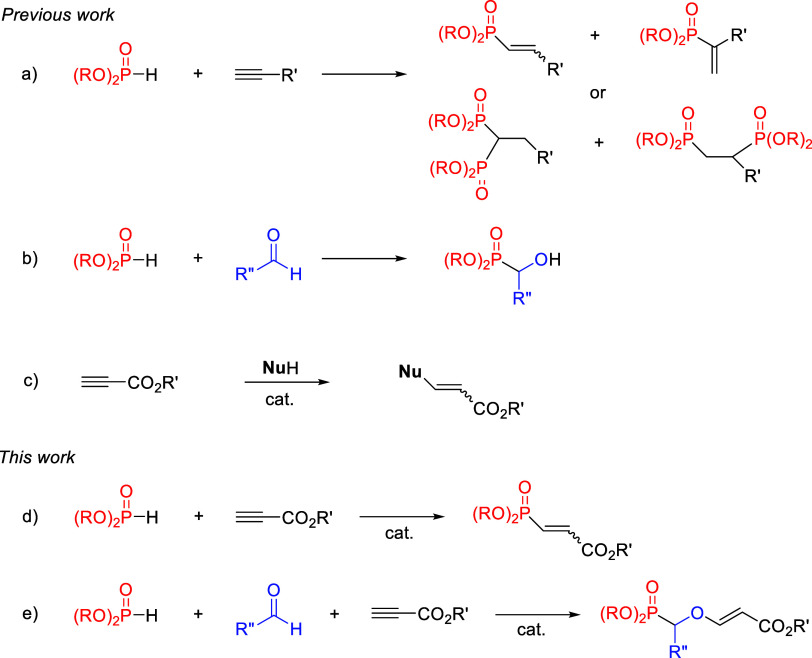
Phosphorylation Reactions and Organocatalyzed Nucleophilic Additions to Propiolates

The hydrophosphorylation of alkynes can lead to Markovnikov and/or anti-Markovnikov addition depending on the reaction conditions. Furthermore, bishydrophosphorylation can also occur due to the nucleophilic addition of a second molecule of H-phosphonate to the newly formed electron-deficient unsaturated system.^4^ Regarding alkynes, the use of propiolates is more scarce^5^ and the corresponding β-phosphonyl acrylate products, which are excellent dienophiles, often need to be prepared using older procedures that rely on less readily available haloacrylates.^6^

Our group has been working over the years on the base-catalyzed addition of nucleophiles onto activated alkynes (Scheme 1c).^7^ This powerful click methodology includes the addition of alcohols, amines, or thiols, among others, but several additional nucleophiles such as cyanide ions or simply water can be used. This strategy relies on the presence of a catalytic amount of a tertiary amine as a nucleophilic catalyst and a pronucleophile containing a relatively acidic hydrogen (more acidic than the propiolate itself, whose p*K*_a_ is reported to be <18.8).^8^ It occurred to us that certain organophosphoros compounds could possibly be candidates for this reaction, as their reported p*K*_a_s are lower than that of methyl propiolate.^9^ Thus, as depicted in Figure 1, unlike H-phosphine oxides or H-phosphinates, H-phosphonates should be sufficiently acidic to be deprotonated in the presence of the alkyne. Therefore, we herein describe our own findings on the DABCO-catalyzed hydrophosphorylation of propiolic esters (Scheme 1d) and its extension to the first multicomponent reaction of H-phosphonates, aldehydes, and alkynes (Scheme 1e).

**Figure 1:**
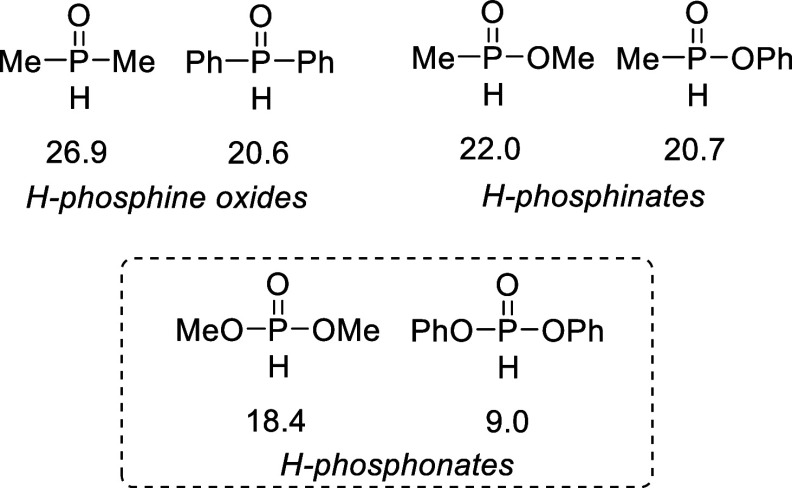
Reported p*K*_a_ values for selected organophosphoros compounds.^9^

## Results and Discussion

Based on our previous knowledge of the reactivity of propiolates, the mechanistic proposal that would sustain our hypothesis is outlined in Scheme 2. Initially, a catalytic amount of a suitable nucleophilic amine would add to the alkyne, delivering the zwitterion **I**, which would be far more basic than the starting catalyst. Most importantly, at this stage, the zwitterion would be protonated by the most acidic hydrogen present in the reaction medium, that is, a suitable H-phosphonate **2**. Thus, ammonium **II** would form along with the corresponding anion **III**. Finally, the formation of **3** would be the consequence of the coupling of those two intermediates and the subsequent elimination of the catalyst from intermediate **IV**. In the case that the organophosphoros compound would not be more acidic than the propiolate itself, the process would be funneled toward the unproductive formation of the dimer **4**. It should be pointed out that the stereochemistry of the double bonds formed during the elimination of the catalyst from intermediate **IV** is anticipated to be predominantly or exclusively the more stable and less hindered *E-*isomer, following the trend of the nucleophiles which have been studied so far.^7^

**Scheme 2:**
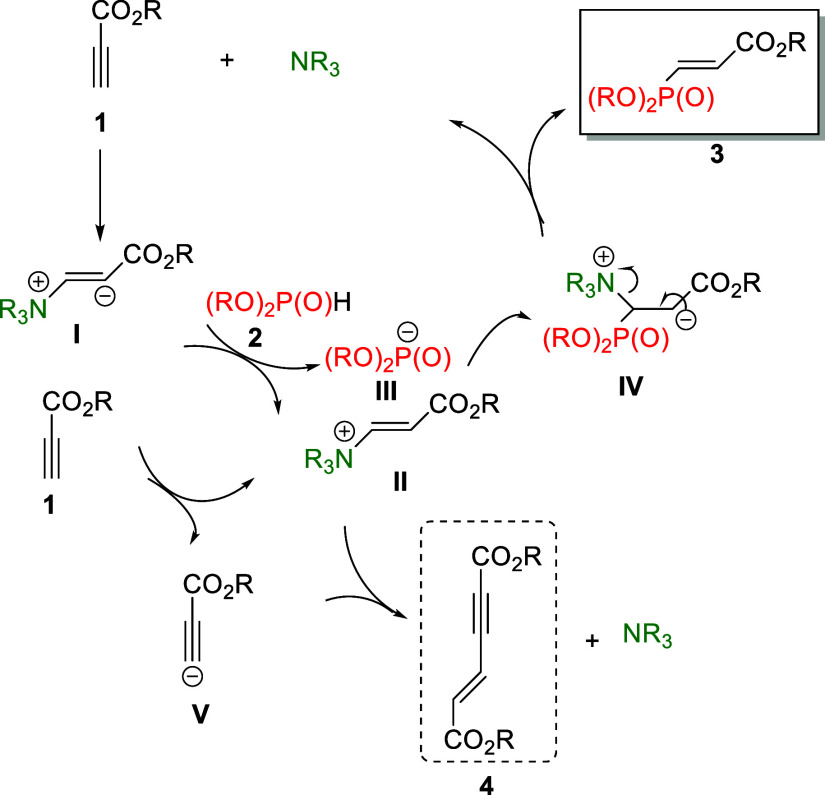
Mechanistic Proposal for the Tertiary Amine-Catalyzed Phosphorylation of Propiolates and the Competitive Formation of Dimer **4**

To begin this study, we chose diphenyl phosphonate because we anticipated a greater chance of success due to its higher acidity compared with other commercially available H-phosphonates. Therefore, we submitted it along with methyl propiolate to our typically most successful reaction conditions (dichloromethane as the solvent and DABCO as the catalyst),^7^ and to our delight, we quickly obtained the desired β-phosphoryl acrylate within minutes at room temperature, in high yield, and with excellent stereoselectivity (only trace amounts of *Z*-isomer were detected). As can be observed in Table 1, the reaction worked better with an excess of the alkyne and using DABCO as the catalyst. The use of at least 0.25 equiv of DABCO was necessary to achieve better yields (entry 5, 85% yield), probably due to the use of commercially available technical grade (85%) diphenyl phosphonate, which may contain impurities that deactivate the catalyst. It should be highlighted at this point that the scalability of the process is straightforward, as the reaction was conducted on a larger scale (10.0 mmol) and the result was maintained (86%, 2.75 g of product **3aa**, entry 6).

**Table 1:**
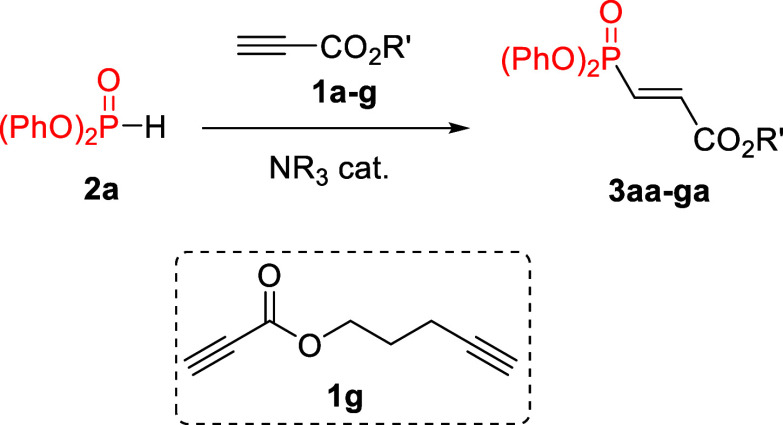
Tertiary Amine-Catalyzed Addition of Diphenyl Phosphonate to Methyl propiolate^a^

	1a–f (equiv)	2a (equiv)	NR_3_ (mol %)	R′	3^b^
1	1.0	1.2	DABCO (10)	Me	**3aa** (59)
2	1.3	1.0	DABCO (10)	Me	(68)
3	1.5	1.0	DABCO (10)	Me	(72)
4	1.5	1.0	DABCO (5)	Me	(traces)
5	1.5	1.0	DABCO (25)	Me	(95) 85
6^c^	1.5	1.0	DABCO (25)	Me	86
7	1.5	1.0	DABCO (50)	Me	(96)
8	1.5	1.0	Et_3_N (10)	Me	(46)
9	1.5	1.0	NMM (10)	Me	0
10	1.5	1.0	DMAP (10)	Me	0
11	1.5	1.0	no cat.	Me	0
12	1.5	1.0	DABCO (25)	Et	**3ba** 88
13	1.5	1.0	DABCO (25)	Oct	**3ca** 87
14	1.5	1.0	DABCO (25)	Bn	**3da** 91
15	1.5	1.0	DABCO (25)	Ph	**3ea** 58
16	1.5	1.0	DABCO (10)	Ph	**3ea** 71
17	1.5	1.0	DABCO (10)	Naph	**3fa** 70
18	1.5	1.0	DABCO (25)	Pent-4-yn	**3ga** 81

a 1 h at room temperature.

b Isolated yields. In parentheses, NMR yields in the crude reaction mixture using Me_3_SiSiMe_3_ as the internal standard.

c Run on a 10 mmol scale of **2a**. NMM = *N*-methyl morpholine. DMAP = 4-(dimethylamino)­pyridine.

Although Et_3_N can be used, it is less efficient (entry 8), and NMM and DMAP are not even able to deliver the desired product under these reaction conditions (entries 9 and 10).^10^ Furthermore, the control experiment without DABCO showed that in the absence of the catalyst, the starting materials remain unreactive (entry 11). With the best conditions at hand, we next explored the use of other readily available alkyl or aryl propiolates, always obtaining the desired products **3aa**–**3ag** in good to excellent yields (entries 12–18). It must be pointed out that aryl propiolates gave lower yields due to the undesired formation of byproducts arising from the reaction of DABCO with the carbonyl group, which thus recommended the use of lower amounts of the catalyst. Finally, and demonstrating the selectivity toward the electron-deficient alkynoate group, propiolate **1g** delivered the desired product **3ga** (81%) with no signs of the phosphorylation product from the terminal unactivated alkyne.

Next, we explored the use of other commercially available dialkyl H-phosphonates, realizing that their P–H bond would behave differently due to its much lower acidity as compared to the diphenyl analog (Table 2). We began studying the use of diethyl phosphonate **2c**, and we soon became aware of its different behavior. An equimolar amount of the phosphonate and methyl propiolate only provided a 16% yield of the desired product **3ac** along with a large amount (62%) of undesired **4a**, implying that the alkyne should be slightly more acidic than the H-phosphonate (entry 2). Because acidity and nucleophilicity are solvent-dependent properties, we anticipated that the relative acidities and nucleophilicities of methyl propiolate and the H-phosphonates would vary in different solvents. After screening a small set of solvents (see Supporting Information, Table S1), we arrived at the conclusion that the use of hexane as the solvent favored the formation of the desired product.^7b^ Thus, we were able to successfully increase the yield of product **3ac** to 75% by using hexane as the solvent, a larger excess of methyl propiolate (2.0 equiv), and 15 mol % of the catalyst (entry 5, this was accompanied by 8% of the *Z*-isomer which was isolated separately). It is important to point out that in this case, products arising from the double addition of diethyl phosphonate start to form albeit in low yield. When using dimethyl phosphonate **2b** (entries 6–8), we observed that although the use of hexane as the solvent was also beneficial in terms of acidity as compared to DCM, its higher nucleophilicity gave rise to more undesired double addition. Satisfyingly, we found that when using a mixture of both solvents (Hex:DCM 8:2), the beneficial effects of each solvent could be emphasized, bringing the yield of the desired product to 81% (entry 8). Dibenzyl phosphonate **2d** was found to be a straightforward case, as its acidity allows for the high-yielding access to product **3ad** in DCM (91%, entry 9). Finally, the more hindered diisopropyl and ditertbutyl phosphonates proved to be much less reactive and delivered the desired products in low yields (35% for **3ae** and only traces of **3af**, entries 9 and 10).

**Table 2:**
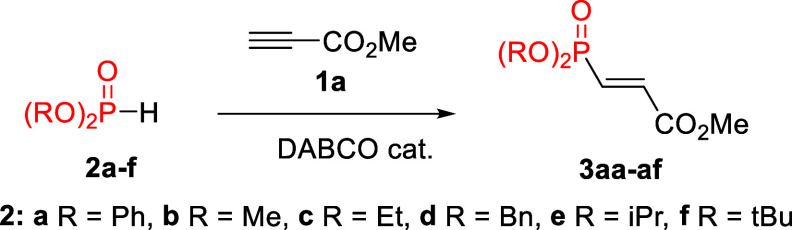
Tertiary Amine-Catalyzed Addition of Disubstituted Phosphonates to Methyl Propiolate^a^

	1a (equiv)	2 (equiv)	R	Solvent	cat. (equiv)	3^b^	4a	bisA^c^	2
1	1.5	**2a** 1.0	Ph	DCM	0.25	**3aa** 85	(6)	n.d.	(13)
2	1.0	**2c** 1.0	Et	DCM	0.1	**3ac** (16)	(62)	n.d.	(44)
3	1.0	**2c** 1.0	Et	Hex	0.1	**3ac** (55)	(10)	(22)	(21)
4	1.5	**2c** 1.0	Et	Hex	0.1	**3ac** (65)	(28)	(20)	(9)
5	2.0	**2c** 1.0	Et	Hex	0.15	**3ac** 75	(43)	(12)	(4)
6	1.5	**2b** 1.0	Me	DCM	0.15	**3ab** (38)	(36)	n.d.	(62)
7	2.0	**2b** 1.0	Me	Hex	0.15	**3ab** (28)	(40)	(60)	n.d.
8	1.5	**2b** 1.0	Me	Hex:DCM 8:2	0.1	**3ab** 81	(18)	(6)	(2)
9	1.5	**2d** 1.0	Bn	DCM	0.1	**3ad** 93	(20)	n.d.	(6)
10	1.5	**2d** 1.0	Bn	Hex	0.1	**3ad** (33)	(20)	(42)	(10)
11	1.5	**2d** 1.0	Bn	Hex:DCM 8:2	0.1	**3ad** (72)	(27)	(16)	(6)
12	1.5	**2e** 1.0	*i*Pr	Hex	0.1	**3ae** (26)	(29)	n.d.	(60)
9	3.0	**2e** 1.0	*i*Pr	Hex	0.1	**3ae** 35	(36)	n.d.	(58)
10	1.5	**2f** 1.0	*t*Bu	Hex	0.1	**3af** (<3)	(50)	n.d.	(97)

a 1 h at room temperature.

b Isolated yields. In parentheses, NMR yields in the crude reaction mixture using Me_3_SiSiMe_3_ as the internal standard.

c Bis-addition products as shown in Scheme 1a. n.d. not detected.

Additionally, we found that the phosphorylation of propiolates can be extended to H-thiophosphonates, as both diphenyl- and dimethyl thiophosphonates gave the desired products in 85% and 57% yields, respectively (Scheme 3). Unfortunately, we also found that the hydrophosphorylation of other electron-deficient alkynes proved to be much more difficult. While alkynones were too reactive and afforded complex mixtures of products, propiolamides or internal alkynoates were unreactive under the conditions studied herein (see Supporting Information).

**Scheme 3:**
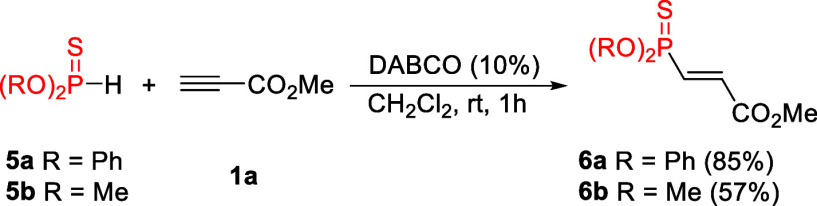
DABCO-Catalyzed Reaction of Methyl Propiolate with H-Thiophosphonates

After studying the reactivity of different organophosphonates with propiolates, we envisioned that a multicomponent reaction could be developed if a proper electrophile was included in the reaction mixture. Thus, analogous to the organocatalytic cyanovinylation of aldehydes carried out in our lab,^11^ in which aldehydes proved to be more electrophilic than intermediate **II** (Scheme 1), the reaction of H-phosphonates, aldehydes, and methyl propiolate delivers phosphomethyl vinyl ethers **8** in a simple procedure and under mild reaction conditions. Following the same trend as above, those reactions using diphenyl phosphonate were performed in dichloromethane (Table 3, entries 1–3 and 11), while those using dialkyl phosphonates were performed in hexane (Table 3, entries 4–10 and 12). Unfortunately, the scope of the reaction regarding the aldehyde component was more or less limited to aromatic aldehydes, as reactions involving an aliphatic aldehyde such as hexanal (entries 11–12) produced the desired products in lower yields (34% and 42%, respectively), accompanied by smaller amounts of the two-component product **3** and mixtures of other unidentified products. This is somewhat unexpected, and we do not have a clear explanation for this trend, since the addition of H–P to carbonyl compounds is known although under different reaction conditions.^3^

**Table 3:**
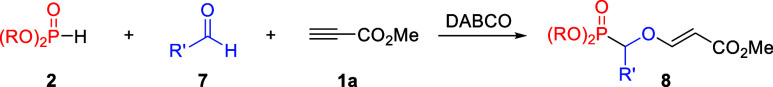
DABCO-Catalyzed Multicomponent Reaction of H-Phosphonate, Aromatic Aldehydes, and Methyl propiolate^a^

	2 R	7 R′	8^b^
1	**2a** Ph	**7a** Ph	**8aa** 61
2	**2a** Ph	**7b** 4-ClPh	**8ab** 73
3	**2a** Ph	**7c** 4-OMePh	**8ac** 48
4	**2b** Me	**7a** Ph	**8ba** 59
5	**2b** Me	**7b** 4-ClPh	**8bb** 54
6	**2b** Me	**7c** 4-OMePh	**8bc** 79
7	**2b** Me	**7d** 3,4,5-(OMe)_3_Ph	**8bd** 80
8	**2b** Me	**7e** 2-thiophenyl	**8be** 73
9	**2b** Me	**7f** 2-naphthyl	**8bf** 55
10	**2c** Et	**7a** Ph	**8ca** 74
11	**2a** Ph	**7g** *n*-Pent	**8ag** 34
12	**2b** Me	**7g** *n*-Pent	**8bg** 42

a 1.0 mmol of H-phosphonate, 1.1 mmol of aldehyde, 1.1 mmol of alkyne, 0.1 mmol of DABCO, 10 mL of solvent (DCM for entries 1–3 and 11, hexane for entries 4–10 and 12), 1 h at room temperature.

b Isolated yield.

## Conclusion

In summary, herein we have reported the practical and efficient hydrophosphorylation of propiolates to access β-phosphoryl acrylates **3** from readily available H-phosphonates and the corresponding alkynes under mild reaction conditions. This transformation is based on the known organocatalytic addition of nucleophiles onto activated alkynes but uses organophosphorus compounds efficiently for the first time. In addition, the process has been extended to develop a new multicomponent synthesis of phosphomethyl vinyl ethers **8** with the successful incorporation of an aldehyde component.

## Experimental Section

### General Remarks

All of the reagents from commercial suppliers were used without further purification. All solvents were freshly distilled before use from the appropriate drying agents. Analytical TLCs were performed with silica gel 60 F254 plates. Visualization was accomplished by the naked eye, by UV light, or by treatment with vanillin in acetic and sulfuric acid in ethanol with heating. Column chromatography was carried out using silica gel 60 (230–400 mesh ASTM). Data were reported as follows: chemical shift, multiplicity (s = singlet, d = doublet, t = triplet, q = quartet, dd = double doublet, m = multiplet, and br = broad), coupling constant (*J* values) in Hz, and integration. High-resolution mass spectra (HRMS) were measured by the ESI method with an Agilent LC-Q-TOF-MS 6520 spectrometer. All H-phosphonates, aldehydes, and alkynes **1a** and **1b** are commercially available, while H-thiophosphonates **5a** ^12^ and **5b** ^13^ and alkynes **1c**–**f** ^7a^ are known and were prepared according to literature procedures.

### General Procedure for the Reaction of H-Phosphonates and Propiolates Catalyzed by DABCO

To an oven-dried round-bottom flask containing the corresponding H-phosphonate **2** (1.00 mmol), were added dry solvent (10 mL) and DABCO (11.2 mg, 0.1 mmol). This was followed by the slow addition of the appropriate propiolate **1** (1.5 mmol). The reaction was stirred for 1 h at room temperature. The solvent was evaporated under reduced pressure to get a crude mixture, which was then subjected to flash chromatography (appropriate mixtures of ethyl acetate:hexanes) to get the desired products (**3**).

### Methyl (*E*)-3-(Diphenoxyphosphoryl)­acrylate **3aa**

261.0 mg, 82% of a white solid purified by column chromatography (ethyl acetate/hexanes = 3:7). ^1^H NMR (CDCl_3_, 400 MHz, δ): 7.32–7.34 (m, 4H), 7.17–7.26 (m, 6H), 7.12 (dd, 1H, *J* = 17.5 and 20.0 Hz), 6.91 (dd, 1H, *J* = 17.5 and 21.8 Hz), 3.81 (s, 3H) ppm. ^13^C­{^1^H} NMR (CDCl_3_, 100 MHz): δ = 164.4 (d, *J* = 30.4), 149.8 (d, *J* = 8.0), 139.2 (d, *J* = 8.0), 130.6 (d, *J* = 189.0), 129.9, 125.6, 120.5 (d, *J* = 4.4), 52.6 ppm. ^31^P­{^1^H} NMR (CDCl_3_, 162 MHz): δ = 7.17 ppm. HRMS (TOF MS ES^+^): *m*/*z* [M + Na]^+^ calculated for C_16_H_15_O_5_PNa 341.0555, found 341.0559. J. Li, Z. Melting point: 56–58 °C. Data in full accordance with that reported in the literature.^5a^ *Z*-isomer (minor): 9 mg, 3% of a pale yellow oil purified by column chromatography (ethyl acetate/hexanes = 1:5): ^1^H NMR (CDCl_3_, 400 MHz, δ): 7.29–7.33 (m, 4H), 7.14–7.22 (m, 6H), 6.70 (dd, 1H, *J* = 13.8 and 50.2 Hz), 6.45 (dd, 1H, *J* = 13.8 and 17.50 Hz), 3.71 (s, 3H) ppm. ^13^C­{^1^H} NMR (CDCl_3_, 100 MHz): δ = 164.3 (d, *J* = 10.5), 150.2 (d, *J* = 8.0), 138.9, 129.8, 128.3 (d, *J* = 190.3), 125.3, 120.6 (d, *J* = 4.4), 52.5 ppm. ^31^P­{^1^H} NMR (CDCl_3_, 162 MHz): δ = 4.07 ppm.

### Ethyl (*E*)-3-(Diphenoxyphosphoryl)­acrylate **3ba**

319.7 mg, 88% of a white solid purified by column chromatography (ethyl acetate/hexanes = 1:4). ^1^H NMR (CDCl_3_, 400 MHz, δ): 7.31–7.35 (m, 4H), 7.18–7.20 (m, 6H), 7.11 (dd, 1H, *J* = 17.3 and 20.0 Hz), 6.91 (dd, 1H, *J* = 17.3 and 21.7 Hz), 4.26 (q, 2H, *J* = 7.0), 1.31 (t, 3H, *J* = 7.0) ppm. ^13^C­{^1^H} NMR (CDCl_3_, 100 MHz): δ = 164.0 (d, *J* = 30.3), 149.8 (d, *J* = 7.3), 139.2 (d, *J* = 7.3), 130.2 (d, *J* = 189.0), 129.9, 125.5, 120.5 (d, *J* = 4.4), 61.7, 14.0 ppm. ^31^P­{^1^H} NMR (CDCl_3_, 162 MHz): δ = 7.38 ppm. HRMS (TOF MS ES^+^): *m*/*z* [M + H]^+^ calculated for C_11_H_18_O_5_P 333.0892, found 333.0898. Melting point: 42–44 °C.

### Octyl (*E*)-3-(Diphenoxyphosphoryl)­acrylate **3ca**

308.7 mg, 87% of a colorless oil purified by column chromatography (ethyl acetate/hexanes = 1:9). ^1^H NMR (CDCl_3_, 400 MHz, δ): 7.31–7.35 (m, 4H), 7.18–7.20 (m, 6H), 7.10 (dd, 1H, *J* = 17.3 and 20.0 Hz), 6.91 (dd, 1H, *J* = 17.3 and 21.8 Hz), 4.19 (t, 2H, *J* = 6.7), 1.63–1.73 m, 2H), 1.24–1.38 (m, 10H), 1.31 (t, 3H, *J* = 6.5) ppm. ^13^C­{^1^H} NMR (CDCl_3_, 100 MHz): δ = 164.1 (d, *J* = 30.5), 149.8 (d, *J* = 7.3), 139.8 (d, *J* = 7.3), 130.7 (d, *J* = 188.9), 129.9, 125.5, 120.5 (d, *J* = 4.4), 65.9, 31.7, 29.12 (2C), 28.4, 25.8, 22.6, 14.1 ppm. ^31^P­{^1^H} NMR (CDCl_3_, 162 MHz): δ = 7.43 ppm. HRMS (TOF MS ES^+^): *m*/*z* [M + H]^+^ calculated for C_23_H_30_O_5_P 417.1831, found 417.1827.

### Benzyl (*E*)-3-(Diphenoxyphosphoryl)­acrylate **3da**

306.6 mg, 91% of a white solid purified by column chromatography (ethyl acetate/hexanes = 1:4): ^1^H NMR (CDCl_3_, 500 MHz, δ): 7.31–7.39 (m, 9H), 7.17–7.20 (m, 6H), 7.14 (dd, 1H, *J* = 17.1 and 20.0 Hz), 6.95 (dd, 1H, *J* = 17.1 and 21.8 Hz), 5.23 (s, 2H) ppm. ^13^C­{^1^H} NMR (CDCl_3_, 100 MHz) δ = 163.8 (d, *J* = 29.9), 149.8 (d, *J* = 8.0), 139.3 (d, *J* = 7.7), 134.9, 130.9 (d, *J* = 189.0), 129.9, 128.7, 128.6, 128.4, 125.6, 120.5 (d, *J* = 4.4), 67.4 ppm. ^31^P­{^1^H} NMR (CDCl_3_, 162 MHz): δ = 7.09 ppm. HRMS (TOF MS ES^+^): *m*/*z* [M + Na]^+^ calculated for C_22_H_19_O_5_PNa 417.0868, found 417.0871. Melting point: 42–44 °C.

### Phenyl (*E*)-3-(Diphenoxyphosphoryl)­acrylate **3ea**

229.6 mg, 71% of a white solid purified by column chromatography (ethyl acetate/hexanes = 4:6): ^1^H NMR (CDCl_3_, 500 MHz, δ): 7.33–7.42 (m, 6H), 7.19–7.30 (m, 8H), 7.06–7.15 (m, 3H) ppm. ^13^C­{^1^H} NMR (CDCl_3_, 100 MHz): δ = 162.4 (d, *J* = 30.5), 150.2, 149.8 (d, *J* = 8.6), 138.7 (d, *J* = 8.2), 132.2 (d, *J* = 188.8), 130.0, 129.6, 126.4, 125.6, 121.1, 120.5 (d, *J* = 4.4) ppm. ^31^P­{^1^H} NMR (CDCl_3_, 162 MHz): δ = 6.57 ppm. HRMS (TOF MS ES^+^): *m*/*z* [M + H]^+^ calculated for C_21_H_18_O_5_P 381.0886, found 381.0892. Melting point: 70–72 °C.

### Naphthalen-1-yl (*E*)-3-(diphenoxyphosphoryl)­acrylate **3fa**

150.6 mg, 70% of a white solid purified by column chromatography (ethyl acetate/hexanes = 2:8): ^1^H NMR (CDCl_3_, 400 MHz, δ): 7.90–7.88 (m, 1H), 7.77–7.82 (m, 2H), 7.50–7.54 (m, 2H), 7.42–7.46 (m, 1H), 7.31–7.36 (m, 6H), 7.21–7.28 (m, 7H) ppm. ^13^C­{^1^H} NMR (CDCl_3_, 100 MHz): δ = 164.5 (d, *J* = 30.5), 149.8 (d, *J* = 7.3), 146.0, 138.5 (d, *J* = 7.3), 134.6, 132.6 (d, *J* = 188.6), 130.0, 128.1, 126.7, 126.64, 126.56, 126.3, 125.7, 125.3, 120.9, 125.6, 120.5 (d, *J* = 4.4), 117.8 ppm. ^31^P­{^1^H} NMR (CDCl_3_, 162 MHz): δ = 6.54 ppm. HRMS (TOF MS ES^+^): *m*/*z* [M + Na]^+^ calculated for C_25_H_19_O_5_PNa 453.0868, found 453.0859. Melting point: 73-74 °C.

### Pent-4-yn-1-yl (*E*)-3-(Diphenoxyphosphoryl)­acrylate **3ga**

149.7 mg, 81% of a white solid purified by column chromatography (ethyl acetate/hexanes = 2:8): ^1^H NMR (CDCl_3_, 400 MHz, δ): 7.31–7.35 (m, 4H), 7.18–7.20 (m, 6H), 7.12 (dd, 1H, *J* = 17.5 and 20.0 Hz), 6.90 (dd, 1H, *J* = 17.5 and 21.6 Hz), 4.31 (t, 2H, *J* = 6.4 Hz), 2.28–2.31 (m, 2H), 1.95 (s, 1H), 1.88–1.92 (m, 2H) ppm. ^13^C­{^1^H} NMR (CDCl_3_, 100 MHz): δ = 163.9 (d, *J* = 30.5), 149.7 (d, *J* = 8.7), 139.4 (d, *J* = 8.5), 130.6 (d, *J* = 188.8), 129.9, 125.6, 120.4 (d, *J* = 4.4), 82.6, 69.3, 64.2, 27.2, 15.1 ppm. ^31^P­{^1^H} NMR (CDCl_3_, 162 MHz): δ = 7.22 ppm. HRMS (TOF MS ES^+^): *m*/*z* [M + Na]^+^ calculated for C_20_H_18_O_5_PNa 393.0868, found 393.0869. Melting point: 83–85 °C.

### Methyl (*E*)-3-(Dimethoxyphosphoryl)­acrylate **3ab**

315.8 mg, 81% of a pale yellow oil purified by column chromatography (ethyl acetate/hexanes = 1:5):^1^H NMR (CDCl_3_, 400 MHz, δ): 6.84 (pseudo t, 1H, *J* = 17.8), 6.70 (dd, 1H, *J* = 17.7 and 20.4 Hz), 3.78 (s, 3H), 3.76 (s, 3H), 3–73 (s, 3H) ppm. ^13^C­{^1^H} NMR (CDCl_3_, 100 MHz): δ = 164.7 (d, *J* = 28.9), 137.6 (d, *J* = 7.3), 130.8 (d, *J* = 184.6), 52.8 (d, *J* = 5.8), 52.4 ppm. ^31^P­{^1^H} NMR (CDCl_3_, 162 MHz): δ = 17.17 ppm. HRMS (TOF MS ES^+^): *m*/*z* [M + H]^+^ calculated for C_6_H_12_O_5_P 195.0422, found 195.0420. Data in full accordance with that reported in the literature.^5a^

### Methyl 3-(Diethoxyphosphoryl)­acrylate **3ac**

*E*-isomer (major): 333.3 mg, 75% of a pale yellow oil purified by column chromatography (ethyl acetate/hexanes = 1:5): ^1^H NMR (CDCl_3_, 400 MHz, δ): 6.88 (pseudo t, 1H, *J* = 17.7), 6.69 (dd, 1H, *J* = 17.7 and 20.5 Hz), 4.07–4.15 (m, 4H), 3.78 (s, 3H), 1.32 (t, 6H, *J* = 7.0) ppm. ^13^C­{^1^H} NMR (CDCl_3_, 100 MHz): δ = 164.9 (d, *J* = 27.8), 136.7 (d, *J* = 6.7), 132.3 (d, *J* = 183.6), 62.5 (d, *J* = 5.8), 52.3, 16.3 (d, *J* = 5.7) ppm. ^31^P­{^1^H} NMR (CDCl_3_, 162 MHz): δ = 14.30 ppm. *Z*-isomer (minor): 35.6 mg, 8% of a pale yellow oil purified by column chromatography (ethyl acetate/hexanes = 1:5): ^1^H NMR (CDCl_3_, 400 MHz, δ): 6.59 (dd, 1H, *J* = 46.8 and 13.9 Hz), 6.18 (t, 1H, *J* = 13.9 Hz), 4.12–4.19 (m, 4H), 3.79 (s, 3H), 1.31 (t, 6H, *J* = 7.0) ppm. ^13^C­{^1^H} NMR (CDCl_3_, 100 MHz): δ = 165.0 (d, *J* = 11.6), 136.7, 129.9 (d, *J* = 186.0), 62.3 (d, *J* = 5.8), 52.2, 16.3 (d, *J* = 5.7) ppm. ^31^P­{^1^H} NMR (CDCl_3_, 162 MHz): δ = 12.02 ppm. Data for both isomers in full accordance with that reported in the literature.^5b^

### Methyl (*E*)-3-(Bis­(benzyloxy)­phosphoryl)­acrylate **3ad**

284.9 mg, 91% of a white solid purified by column chromatography (ethyl acetate/hexanes = 1:1): ^1^H NMR (CDCl_3_, 400 MHz, δ): 7.30–7.38 (m, 10H), 7.12 (pseudo t, 1H, *J* = 18.0 Hz), 6.91 (dd, 1H, *J* = 18.0 and 20.7 Hz), 5.00–5.08 (m, 2H), 3.76 (s, 3H) ppm. ^13^C­{^1^H} NMR (CDCl_3_, 100 MHz): δ = 164.7 (d, *J* = 29.1), 136.4 (d, *J* = 7.0), 135.6 (d, *J* = 5.7), 131.3 (d, *J* = 186.0), 128.6 (2C), 128.0, 67.9 (d, *J* = 5.8), 52.3 ppm. ^31^P­{^1^H} NMR (CDCl_3_, 162 MHz): δ = 15.41 ppm. HRMS (TOF MS ES^+^): *m*/*z* [M + Na]^+^ calculated for C_18_H_19_O_5_PNa 369.0868, found 369.0872. Melting point: 67–69 °C. Data in full accordance with that reported in the literature.^5a^

### Methyl (*E*)-3-(Diisopropoxyphosphoryl)­acrylate **3ae**

177.0 mg, 35% of a pale yellow oil purified by column chromatography (ethyl acetate/hexanes = 1:5): ^1^H NMR (CDCl_3_, 400 MHz, δ): 6.88 (pseudo t, 1H, *J* = 17.7), 6.66 (dd, 1H, *J* = 17.7 and 20.5 Hz), 4.66–4.71 (m, 2H), 3.77 (s, 3H), 1.28–1.34 (m, 12H) ppm. ^13^C­{^1^H} NMR (CDCl_3_, 100 MHz): δ = 165.1 (d, *J* = 27.8), 135.9 (d, *J* = 7.3), 133.9 (d, *J* = 184.8), 71.4 (d, *J* = 5.9), 52.3, 24.0 (d, *J* = 4.3), 23.9 (d, *J* = 4.3) ppm. ^31^P­{^1^H} NMR (CDCl_3_, 162 MHz): δ = 12.02 ppm. HRMS (TOF MS ES^+^): *m*/*z* [M + H]^+^ calculated for C_10_H_20_O_5_P 251.1048, found 251.1043. Data in full accordance with that reported in the literature.^5a^

### General Procedure for the Reaction of H-Thiophosphonates and Propiolates Catalyzed by DABCO

To an oven-dried round-bottom flask containing the corresponding H-thiophosphonate **5** (1.00 mmol, 1.0 equiv), were added dry dichloromethane (10 mL, 0.1 M) and methyl propiolate **1a** (1.5 mmol, 1.5 equiv). This was followed by the addition of DABCO (0.1 mmol, 0.1 equiv). The reaction was stirred for 1 h at room temperature. The solvent was evaporated under reduced pressure to get a crude mixture, which was then subjected to flash chromatography (appropriate mixtures of dichloromethane:hexanes) to get the desired products (**6**).

### Methyl (*E*)-3-(Diphenoxythiophosphoryl)­acrylate **6a**

115.5 mg, 85% of a colorless oil purified by column chromatography (dichloromethane/hexanes = 1:4): ^1^H NMR (CDCl_3_, 500 MHz, δ): 7.40 (dd, 1H, *J* = 16.7 and 18.8 Hz), 7.35 (t, 4H, *J* = 7.8 Hz), 7.22 (td, 2H, *J* = 7.3 and 1.2 Hz), 7.15 (ddd, 4H, *J* = 8.5, 1.9, and 1.1 Hz), 6.94 (dd, 1H, *J* = 23.8 and 16.7 Hz), 3.85 (s, 4H) ppm. ^13^C­{^1^H} NMR (CDCl_3_, 125 MHz): δ = 164.9 (d, *J* = 30.7 Hz), 150.1 (d, *J* = 8.9 Hz), 137.5 (t, *J* = 5.4 Hz), 136.3, 129.8 (d, *J* = 1.7 Hz), 125.8 (d, *J* = 2.1 Hz), 121.8 (d, *J* = 4.6 Hz), 52.7. ^31^P­{^1^H} NMR (CDCl_3_, 162 MHz): δ = 76.36 ppm. HRMS (TOF MS ES^+^): *m*/*z* [M + H]^+^ calculated for C_16_H_16_O_4_PS 335.0507, found 335.0506.

### Methyl (*E*)-3-(Dimethoxythiophosphoryl)­acrylate **6b**

225.8 mg, 57% of a colorless oil purified by column chromatography (dichloromethane/hexanes = 1:4): ^1^H NMR (CDCl_3_, 500 MHz, δ): 7.00 (dd, 1H, *J* = 18.6 and 16.8 Hz), 6.69 (dd, 1H, *J* = 22.5 and 16.8 Hz), 3.79 (s, 3H), 3.73 (d, 6H, *J* = 13.8 Hz) ppm. ^13^C­{^1^H} NMR (CDCl_3_, 125 MHz): δ = 165.1 (d, *J* = 30.0 Hz), 136.9, 135.9, 53.2 (d, *J* = 5.8), 52.4 ppm. ^31^P­{^1^H} NMR (CDCl_3_, 162 MHz): δ = 85.09 ppm. HRMS (TOF MS ES^+^): *m*/*z* [M + H]^+^ calculated for C_6_H_12_O_4_PS 211.0188, found 211.0199.

### General Procedure for the Multicomponent Reaction of H-Phosphonates, Aldehydes, and Methyl Propiolate Catalyzed by DABCO

To an oven-dried round-bottom flask containing the corresponding H-phosphonate **2** (1.00 mmol, 1.0 equiv), were added dry solvent (10 mL, 0.1 M), aldehyde **7** (1.1 mmol, 1.1 equiv), methyl propiolate **1a** (1.1 mmol, 1.1 equiv), and finally DABCO (0.1 mmol, 0.1 equiv). The reaction was stirred for 1 h at room temperature. The solvent was evaporated under reduced pressure to get a crude mixture, which was then subjected to flash chromatography (appropriate mixtures of ethyl acetate: hexanes) to get the desired products (**8**).

### Methyl (*E*)-3-((Diphenoxyphosphoryl)­(phenyl)­methoxy)­acrylate **8aa**

258.9 mg, 61% of a white solid purified by column chromatography (ethyl acetate/hexanes = 4:6): ^1^H NMR (CDCl_3_, 500 MHz, δ): 7.55 (d, 1H, *J* = 12.4 Hz), 7.50–7.52 (m, 2H), 7.38–7.42 (m, 3H), 7.25–7.28 (m, 4H), 7.13–7.16 (m, 2H), 6.99–7.01 (m, 4H), 5.47 (d, 1H, *J* = 14.4 Hz), 5.37 (d, 1H, *J* = 12.4 Hz), 3.65 (s, 3H) ppm. ^13^C­{^1^H} NMR (CDCl_3_, 100 MHz): δ = 167.2, 160.6 (d, *J* = 14.5), 150.1 (d, *J* = 10.1), 149.9 (d, *J* = 8.9), 131.5 (d, *J* = 2.9), 129.8, 129.7, 129.5 (d, *J* = 4.4), 129.0, 127.9 (d, J = 6.1), 125.5, 125.4, 120.4 (d, *J* = 4.4), 100.3, 78.9 (d, *J* = 174.0), 51.3 ppm. ^31^P­{^1^H} NMR (CDCl_3_, 162 MHz): δ = 8.33 ppm. HRMS (TOF MS ES^+^): *m*/*z* [M + Na]^+^ calculated for C_23_H_21_O_6_PNa 447.0968, found 447.0974. Melting point: 115–117 °C.

### Methyl (*E*)-3-((4-Chlorophenyl)­(diphenoxyphosphoryl)­methoxy)­acrylate **8ab**

334.6 mg, 73% of a white solid purified by column chromatography (ethyl acetate/hexanes = 3:7): ^1^H NMR (CDCl_3_, 400 MHz, δ): 7.53 (d, 1H, *J* = 12.4 Hz), 7.42–7.45 (m, 2H), 7.35–7.39 (m, 2H), 7.24–7.29 (m, 4H), 7.14–7.18 (m, 2H), 7.01–7.04 (m, 4H), 5.44 (d, 1H, *J* = 14.4 Hz), 5.36 (d, 1H, *J* = 12.4 Hz), 3.66 (s, 3H) ppm. ^13^C­{^1^H} NMR (CDCl_3_, 100 MHz): δ = 167.0, 160.2 (d, *J* = 14.4), 150.0 (d, *J* = 10.1), 149.7 (d, *J* = 9.5), 135.6 (d, *J* = 4.6), 130.1 (d, *J* = 3.1), 129.8, 129.7, 129.16, 129.14 (d, *J* = 9.5), 125.5 (d, *J* = 7.8), 120.28, 120.26, 120.20, 100.5, 77.6 (d, *J* = 174.5), 51.3 ppm. ^31^P­{^1^H} NMR (CDCl_3_, 162 MHz): δ = 7.70 ppm. HRMS (TOF MS ES^+^): *m*/*z* [M + Na]^+^ calculated for C_23_H_20_^35^ClO_6_PNa 481.0584, found 481.0582. Melting point: 108–110 °C.

### Methyl (*E*)-3-((Diphenoxyphosphoryl)­(4-methoxyphenyl)­methoxy)­acrylate **8ac**

218.1 mg, 48% of a white solid purified by column chromatography (ethyl acetate/hexanes = 3:7): ^1^H NMR (CDCl_3_, 400 MHz, δ): 7.53 (d, 1H, *J* = 12.4 Hz), 7.42–7.44 (m, 2H), 7.24–7.29 (m, 4H), 7.12–7.17 (m, 2H), 7.05 (d, 2H, *J* = 8.1 Hz), 7.00 (d, 2H, *J* = 8.1 Hz), 6.92 (d, 2H, *J* = 8.4 Hz), 5.41 (d, 1H, *J* = 14.4 Hz), 5.38 (d, 1H, *J* = 12.4 Hz), 3.80 (s, 3H), 3.65 (s, 3H) ppm. ^13^C­{^1^H} NMR (CDCl_3_, 100 MHz): δ = 167.3, 160.54, 160.52 (d, *J* = 14.5), 160.51, 150.1 (d, *J* = 10.1), 149.9 (d, *J* = 8.9), 129.7 (d, *J* = 2.9), 129.5 (d, *J* = 4.4), 120.4, 120.35, 120.3, 123.2 (d, *J* = 4.4), 114.4, 100.1, 78.6 (d, *J* = 177.0), 55.3, 51.2 ppm. ^31^P­{^1^H} NMR (CDCl_3_, 162 MHz): δ = 8.67 ppm. HRMS (TOF MS ES^+^): *m*/*z* [M + Na]^+^ calculated for C_24_H_23_O_7_PNa 477.1079, found 477.1089. Melting point: 93–94 °C.

### Methyl (*E*)-3-((Dimethoxyphosphoryl)­(phenyl)­methoxy)­acrylate **8ba**

267.9 mg, 59% of a colorless oil purified by column chromatography (ethyl acetate/hexanes = 3:7): ^1^H NMR (CDCl_3_, 400 MHz, δ): 7.47 (d, 1H, *J* = 12.4 Hz), 7.44–7.29 (m, 5H), 5.31 (d, 1H, *J* = 12.5 Hz), 5.16 (d, 1H, *J* = 14.3 Hz), 3.70 (d, 3H, *J* = 10.5 Hz), 3.64 (d, 3H, *J* = 10.2 Hz), 3.62 (s, 3H) ppm. ^13^C­{^1^H} NMR (CDCl_3_, 100 MHz): δ = 167.4, 161.0 (d, *J* = 13.5 Hz), 132.5 (d, *J* = 2.8 Hz), 129.3 (d, *J* = 3.0 Hz), 128.9 (d, *J* = 2.4 Hz), 127.6 (d, *J* = 5.7 Hz), 99.9, 79.4 (d, *J* = 170.8 Hz), 54.3 (d, *J* = 7.0 Hz), 54.0 (d, *J* = 6.9 Hz), 51.3 ppm. ^31^P­{^1^H} NMR (CDCl_3_, 162 MHz): δ = 18.29 ppm. HRMS (TOF MS ES^+^): *m*/*z* [M + H]^+^ calculated for C_13_H_17_O_6_PNa 323.0655, found 323.0660.

### Methyl (*E*)-3-((4-Chlorophenyl)­(dimethoxyphosphoryl)­methoxy)­acrylate **8bb**

180.7 mg, 54% of a colorless oil purified by column chromatography (ethyl acetate/hexanes = 3:7): ^1^H NMR (CDCl_3_, 400 MHz, δ): 7.46 (d, 1H, *J* = 12.5 Hz), 7.42–7.32 (m, 4H), 5.31 (d, 1H, *J* = 12.4 Hz), 5.13 (d, 1H, *J* = 14.9 Hz), 3.75 (d, 3H, *J* = 10.7 Hz), 3.69 (d, 3H, *J* = 10.7 Hz), 3.66 (s, 3H) ppm. ^13^C­{^1^H} NMR (CDCl_3_, 100 MHz): δ = 167.4, 160.7 (d, *J* = 13.3 Hz), 135.5 (d, *J* = 3.8 Hz), 131.1 (d, *J* = 2.7 Hz), 129.3 (d, *J* = 2.5 Hz), 128.9 (d, *J* = 5.5 Hz), 100.3, 78.9 (d, *J* = 171.4 Hz), 54.5 (d, *J* = 6.9 Hz), 54.1 (d, *J* = 6.9 Hz), 51.5 ppm. ^31^P­{^1^H} NMR (CDCl_3_, 162 MHz): δ = 17.76 ppm. HRMS (TOF MS ES^+^): *m*/*z* [M + H]^+^ calculated for C_13_H_16_O_7_P­[^35^Cl]Na 357.0280, found 357.0266; and for C_13_H_16_O_7_P­[^37^Cl]Na 359.0250, found 359.0242.

### Methyl (*E*)-3-((Dimethoxyphosphoryl)­(4-methoxyphenyl)­methoxy)­acrylate **8bc**

260.9 mg, 79% of a colorless oil purified by column chromatography (ethyl acetate/hexanes = 3:7): ^1^H NMR (CDCl_3_, 400 MHz, δ): 7.47 (d, 1H, *J* = 12.4 Hz), 7.35 (dd, 2H, *J* = 8.9 and 1.6 Hz), 6.92 (d, 2H, *J* = 8.3 Hz), 5.32 (d, 1H, *J* = 12.4 Hz), 5.10 (d, 1H, *J* = 14.2 Hz), 3.81 (s, 3H), 3.75 (d, 3H, *J* = 10.7 Hz), 3.65 (d, 3H, *J* = 10.6 Hz), 3.65 (s, 3H) ppm. ^13^C­{^1^H} NMR (CDCl_3_, 100 MHz): δ = 167.4, 161.0 (d, *J* = 13.8 Hz), 129.2 (d, *J* = 5.8 Hz), 124.3 (d, *J* = 2.8 Hz), 114.5 (d, *J* = 2.2 Hz), 99.9, 79.20 (d, *J* = 173.6 Hz), 55.4, 54.2 (d, *J* = 6.9 Hz), 54.0 (d, *J* = 6.9 Hz), 51.3 ppm. ^31^P­{^1^H} NMR (CDCl_3_, 162 MHz): δ = 18.65 ppm. HRMS (TOF MS ES^+^): *m*/*z* [M + H]^+^ calculated for C_14_H_19_O_7_PNa 353.0761, found 353.0764.

### Methyl (*E*)-3-((Dimethoxyphosphoryl)­(3,4,5-trimethoxyphenyl)­methoxy)­acrylate **8bd**

The synthesis was carried out in 1:9 DCM/Hexane to aid in solubilizing the aldehyde. 292.8 mg, 75% of a colorless oil purified by column chromatography (ethyl acetate/hexanes = 3:7): ^1^H NMR (CDCl_3_, 400 MHz, δ): 7.47 (d, 1H, *J* = 12.4 Hz), 6.63 (d, 2H, *J* = 2.3 Hz), 5.36 (d, 1H, *J* = 12.4 Hz), 5.05 (d, 1H, *J* = 14.6 Hz), 3.86 (s, 6H), 3.84 (s, 3H), 3.77 (d, 3H, *J* = 10.7 Hz), 3.67 (d, 3H, *J* = 10.7 Hz), 3.66 (s, 3H) ppm. ^13^C­{^1^H} NMR (CDCl_3_, 100 MHz): δ = 167.6, 161.0 (d, *J* = 13.8 Hz), 153.7 (d, *J* = 2.6 Hz), 138.8, 127.8 (d, *J* = 2.5 Hz), 104.8 (d, *J* = 5.8 Hz), 100.1, 79.6 (d, *J* = 172.3 Hz), 61.0 (d, *J* = 1.8 Hz), 56.4, 54.4 (d, *J* = 6.9 Hz), 54.0 (d, *J* = 6.9 Hz), 51.4 ppm. ^31^P­{^1^H} NMR (CDCl_3_, 162 MHz): δ = 18.36 ppm. HRMS (TOF MS ES^+^): *m*/*z* [M + H]^+^ calculated for C_16_H_23_O_9_PNa 413.0977, found 413.0983. Melting point: 110–112 °C.

### Methyl (*E*)-3-((Dimethoxyphosphoryl)­(thiophen-2-yl)­methoxy)­acrylate **8be**

334.5 mg, 73% of a yellowish oil purified by column chromatography (ethyl acetate/hexanes = 3:7): ^1^H NMR (CDCl_3_, 400 MHz, δ): 7.48 (d, 1H, *J* = 12.4 Hz), 7.40 (d, 1H, *J* = 5.0 Hz), 7.23 (t, 1H, *J* = 3.3 Hz), 7.04 (t, 1H, *J* = 4.3 Hz), 5.42 (d, 1H, *J* = 12.4 Hz), 5.39 (d, 1H, *J* = 14.8 Hz), 3.80 (d, 3H, *J* = 10.7 Hz), 3.72 (d, 3H, *J* = 10.7 Hz), 3.66 (s, 3H) ppm. ^13^C­{^1^H} NMR (CDCl_3_, 100 MHz): δ = 167.5, 160.6 (d, *J* = 12.2 Hz), 134.2, 128.9 (d, *J* = 7.9 Hz), 128.1 (d, *J* = 3.1 Hz), 127.4 (d, *J* = 2.3 Hz), 100.3, 75.6 (d, *J* = 178.4 Hz), 54.5 (d, *J* = 6.9 Hz), 54.3 (d, *J* = 6.7 Hz), 51.4 ppm. ^31^P­{^1^H} NMR (CDCl_3_, 162 MHz): δ = 17.01 ppm. HRMS (TOF MS ES^+^): *m*/*z* [M + H]^+^ calculated for C_11_H_15_O_6_PSNa 329.0225, found 329.0225.

### Methyl (*E*)-3-((Dimethoxyphosphoryl)­(naphthalen-1-yl)­methoxy)­acrylate **8bf**

192.7 mg, 55% of a colorless oil purified by column chromatography (ethyl acetate/hexanes = 6:4): ^1^H NMR (CDCl_3_, 500 MHz, δ): 7.90–7.88 (m, 2H), 7.86 (ddd, 2H, *J* = 9.9, 5.2, 2.1 Hz), 7.55–7.51 (m, 4H), 5.37 (d, 1H, *J* = 12.6 Hz), 5.34 (d, 1H, *J* = 14.8 Hz), 3.74 (d, 3H, *J* = 10.7 Hz), 3.67 (d, 3H, *J* = 10.6 Hz), 3.62 (s, 3H) ppm. ^13^C­{^1^H} NMR (CDCl_3_, 125 MHz): δ = 167.3, 160.8 (d, *J* = 13.8 Hz), 133.6 (d, *J* = 2.3 Hz), 133.1 (d, *J* = 2.4 Hz), 129.9 (d, *J* = 3.2 Hz), 129.0 (d, *J* = 2.2 Hz), 128.3, 127.9, 127.3 (d, *J* = 7.4 Hz), 127.0, 126.8, 124.6 (d, *J* = 4.2 Hz), 100.1, 79.6 (d, *J* = 171.4 Hz), 54.4 (d, *J* = 6.9 Hz), 54.1 (d, *J* = 6.9 Hz), 51.4 ppm. ^31^P­{^1^H} NMR (CDCl_3_, 162 MHz): δ = 18.25 ppm. HRMS (TOF MS ES^+^): *m*/*z* [M + H]^+^ calculated for C_17_H_19_O_6_PNa 373.0817, found 373.0819.

### Methyl (*E*)-3-((Diethoxyphosphoryl)­(phenyl)­methoxy)­acrylate **8ca**

242.9 mg, 74% of a colorless oil purified by column chromatography (ethyl acetate/hexanes = 3:7): ^1^H NMR (CDCl_3_, 400 MHz, δ): 7.49 (d, 1H, *J* = 12.4 Hz), 7.31–7.40 (m, 5H), 5.31 (d, 1H, *J* = 12.4 Hz), 5.12 (d, 1H, *J* = 14.6 Hz), 3.98–4.12 (m, 3H), 3.88–3.98 (m, 1H), 3.62 (s, 3H), 1.19–1.26 (m, 6H) ppm. ^13^C­{^1^H} NMR (CDCl_3_, 100 MHz): δ = 167.4, 161.1 (d, *J* = 13.1), 132.5 (d, *J* = 2.9), 129.0 (d, *J* = 3.0), 128.7 (d, *J* = 2.7), 127.5 (d, *J* = 5.6), 99.6, 79.6 (d, *J* = 170.1), 63.7 (d, *J* = 7.2), 63.5 (d, *J* = 7.2), 51.2, 16.4 (d, *J* = 5.8), 16.3 (d, *J* = 5.8) ppm. ^31^P­{^1^H} NMR (CDCl_3_, 162 MHz): δ = 15.98 ppm. HRMS (TOF MS ES^+^): *m*/*z* [M + Na]^+^ calculated for C_25_H_21_O_6_PNa 351.0973, found 351.0974.

### Methyl (*E*)-3-((1-(Diphenoxyphosphoryl)­hexyl)­oxy)­acrylate **8ag**

141.2 mg, 34% of a colorless oil purified by column chromatography (ethyl acetate/hexanes = 3:7): ^1^H NMR (CDCl_3_, 400 MHz, δ): 7.54 (d, 1H, *J* = 12.2 Hz), 7.28–7.34 (m, 4H), 7.12–7.20 (m, 6H), 5.42 (d, 1H, *J* = 12.2 Hz), 4.37–4.44 (m, 1H), 3.69 (s, 3H), 2.00–2.05 (m, 2H), 1.55–1.63 (m, 1H), 1.30–1.48 (m, 5H), 0.88 (t, 3H, *J* = 6.8 Hz) ppm. ^13^C­{^1^H} NMR (CDCl_3_, 100 MHz): δ = 167.7, 162.3 (d, *J* = 4.2 Hz), 150.1 (d, *J* = 10.2), 149.9 (d, *J* = 8.7), 129.9, 129.8, 125.54, 125.50, 120.49, 120.45, 99.3, 78.9 (d, *J* = 167.0 Hz), 51.3, 31.2, 29.8, 25.2 (d, *J* = 12.4 Hz), 22.3, 13.9 ppm. ^31^P­{^1^H} NMR (CDCl_3_, 162 MHz): δ = 12.33 ppm. HRMS (TOF MS ES^+^): *m*/*z* [M + Na]^+^ calculated for C_22_H_27_O_6_PNa 441.1443, found 441.1445.

### Methyl (*E*)-3-((1-(Dimethoxyphosphoryl)­hexyl)­oxy)­acrylate **8bg**

122.4 mg, 42% of a colorless oil purified by column chromatography (ethyl acetate/hexanes = 1:1): ^1^H NMR (CDCl_3_, 400 MHz, δ): 7.45 (d, 1H, *J* = 12.2 Hz), 5.38 (d, 1H, *J* = 12.2 Hz), 4.07–4.12 (m, 1H), 3.80 (d, 3H, *J* = 10.5 Hz), 3.77 (d, 3H, *J* = 10.7 Hz), 3.68 (s, 3H), 1.80–1.88 (m, 2H), 1.53–1.45 (m, 1H), 1.40–1.22 (m, 5H), 0.86 (t, 3H, *J* = 6.8 Hz) ppm. ^13^C­{^1^H} NMR (CDCl_3_, 100 MHz): δ = 167.8, 162.4 (d, *J* = 4.2 Hz), 98.7, 79.2 (d, *J* = 166.5 Hz), 53.6 (d, *J* = 6.9 Hz), 53.1 (d, *J* = 6.9 Hz), 51.2, 31.2, 29.7, 25.1 (d, *J* = 12.4 Hz), 22.3, 13.9 ppm. ^31^P­{^1^H} NMR (CDCl_3_, 162 MHz): δ = 21.99 ppm. HRMS (TOF MS ES^+^): *m*/*z* [M + Na]^+^ calculated for C_12_H_23_O_6_PNa 317.1130, found 317.1131.

## Supplementary Material



## Data Availability

The data underlying this study are available in the published article and its Supporting Information

## Notes

^1^H, ^13^C, and ^31^PNMR spectra of all products (PDF)
