# Supplementary material for: Catalytic Hydrophosphorylation of Propiolates and Three-Component Phosphorylation of Aldehydes
Source: J Org Chem. 2026 Jan 21;91(5):1977–85. doi: 10.1021/acs.joc.5c02398 (PMC12887989; doi:10.1021/acs.joc.5c02398)
Supplement: Supplementary file 1 [file jo5c02398_si_001.pdf]

Supporting Information

Catalytic Hydrophosphorylation of Propiolates and a Three-Component Phosphorylation of Aldehydes.

Samuel Delgado-Hernández,[1] Alejandro Peixoto de Abreu Lima,[2] Eva M. Martín-Díaz,[1] Jimena Scoccia,[1] Romen Carrillo[1]* and David Tejedor[1]*

[1]Instituto de Productos Naturales y Agrobiología, Consejo Superior de Investigaciones Científicas, Avda. Astrofísico Francisco Sánchez 3, 38 206 La Laguna, Tenerife, Islas Canarias, Spain.

[2] Departamento de Química Orgánica, Facultad de Química, Universidad de la República, Av. General Flores 2124, 11800, Montevideo, Uruguay; and Instituto Pasteur de Montevideo, Mataojo 2020, 11400, Montevideo, Uruguay.

E-mail: rcarrillo@ipna.csic.es; dtejedor@ipna.csic.es

Table of contents

1. General Information S2

2. Table S1. DABCO-catalyzed addition of diethyl phosphonate to methyl propiolate S2

3. Scaled-up synthesis of 3aa S2

4. Unsuccessful alkynes S3

5. Experimentally observed relative acidities S3

6. ^{1}H NMR, ^{13}C NMR and ^{31}P NMR Spectra S4-S44

Generaleral Information

All reagents from commercial suppliers were used without further purification. All solvents were freshly distilled before use from appropriate drying agents. Analytical TLCs were performed with silica gel 60 F254 plates. Visualization was accomplished by naked eye, or by UV light or vanillin with acetic and sulfuric acid in ethanol with heating. Column chromatography was carried out using silica gel 60 (230-400 mesh ASTM). ^{1}H NMR spectra were recorded at 500 MHz and 400MHz, ^{13}C NMR spectra were recorded at 125 MHz and 100 MHz. NaOH lentils were added to $CDCl_3$ to prevent problems with the residual acid content. High resolution mass spectra (HRMS) were measured by ESI method with an Agilent LC-Q-TOF-MS 6520 spectrometer.

Table S1. DABCO-catalyzed addition of diethyl phosphonate to methyl propiolate.[a]

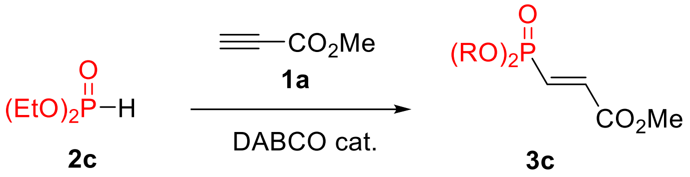

	1a (eq)	Solvent	cat. (eq)	3c (E)[b]	3c (Z)[b]	4a	bisA[c]	2c
1	1.0	DCM	0.1	16	<1	62	n.d.	44
2	1.0	CH_3CN	0.1	25	3	29	n.d.	64
3	1.0	Benzene	0.1	24	<1	42	n.d.	61
4	1.0	Hex	0.1	55	3	10	22	21
5	1.5	Hex	0.1	65	6	28	20	9
6	2.0	Hex	0.1	65	8	51	20	3
**7**	**2.0**	**Hex**	**0.15**	**75**	**8**	**43**	**12**	**4**
8	1.5	Hex	0.05	52	5	11	30	12
9	1.5	Hex	0.2	68	6	20	17	6
10	1.5	Hex/DCM (8:2)	0.1	54	2	55	1	42
11	1.5	Hex/DCM (9:1)	0.1	61	4	56	3	32

[a] 1h at room temperature. Concentration = 0.2M [b] NMR yields in crude reaction mixture using $Me_3SiSiMe_3$ as internal standard. [c] Bis-addition products as shown in scheme 1a. n.d. not detected

Scaled-up synthesis of diphenyl phosphonate and methyl propiolate catalyzed by DABCO

To an oven dried round bottom flask containing the diphenyl phosphonate **2a** (2.76 g of 85% purity, 10.0 mmol), was added dry solvent (100 mL) and DABCO (280 mg, 2.5 mmol). This was followed by slow addition of methyl propiolate **1** (1.26 g, 15 mmol). The reaction was stirred for one hour at room temperature. The solvent was evaporated under reduced pressure to get a crude mixture which was then subjected to Flash chromatography 20% - 50% acetate: hexanes) to get the desired products ***E*-3aa** (2.75 g, 86%) and ***Z*-3aa** (198 mg, 6%).

Unsuccessful alkynes

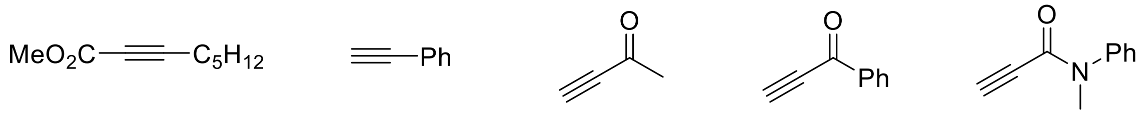

We have also tested other alkynes in our transformations. Alkynones such as 1-phenyl-2-propyn-1-one or 3-butyn-2-one were too reactive and afforded complex mixtures of products which were difficult to separate. We postulate that the enones which would be initially obtained are be more electron deficient and more reactive than the enoates, and they participate in further phosphorylations, giving complex mixtures that include bisaddition products.

An internal alkyne such as methyl 2-octynoate or an unactivated alkyne such as phenylacetylene remained unreactive in the presence of DABCO catalyst. This is in accordance with previous results from our group and others for the well know hydroxyl-yne reaction (see for example references 7a-h in the main text).

Unfortunately we could not obtain positive results when using a propiolamide, as it was unreactive under the conditions studied herein.

Experimentally observed relative acidities

Complementing the pKa's given in references 8 and 9, we present the experimentally observed relative acidities.

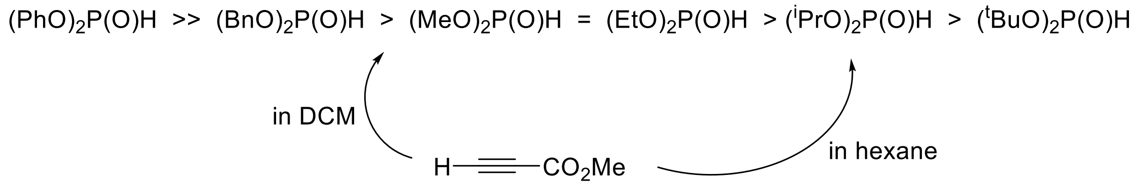

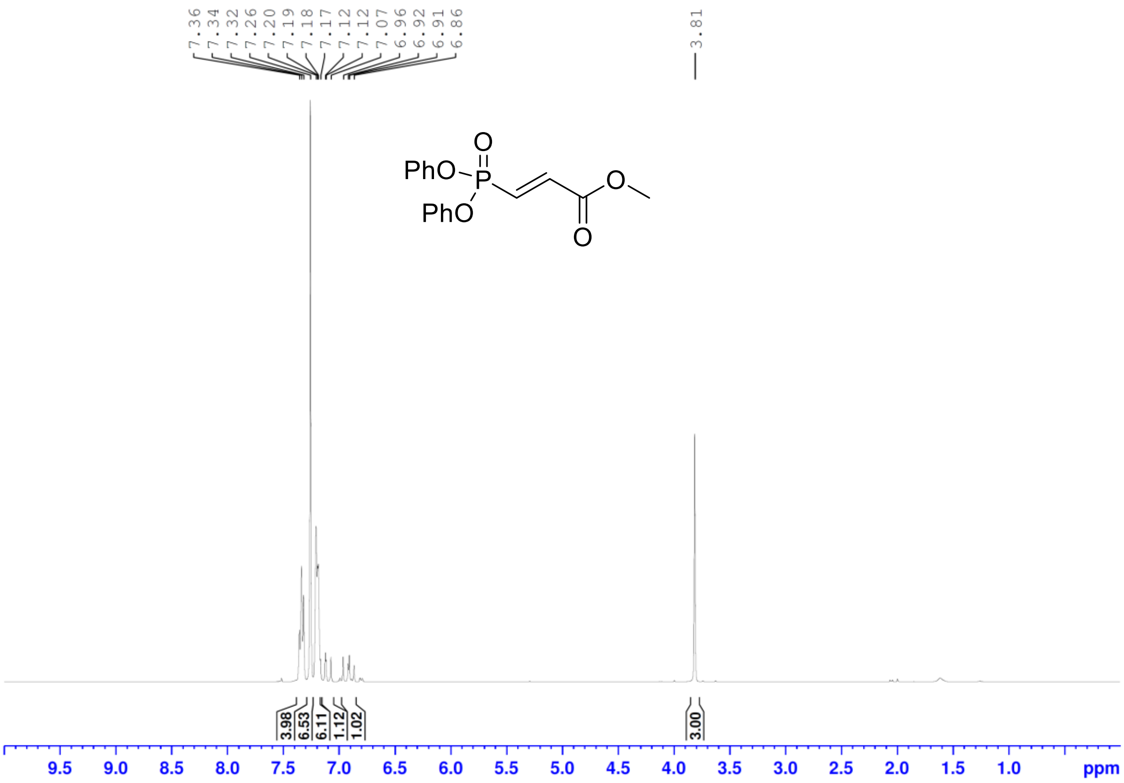

^{1}H NMR (400 MHz, $CDCl_3$) of compound **3aa (*E*)**

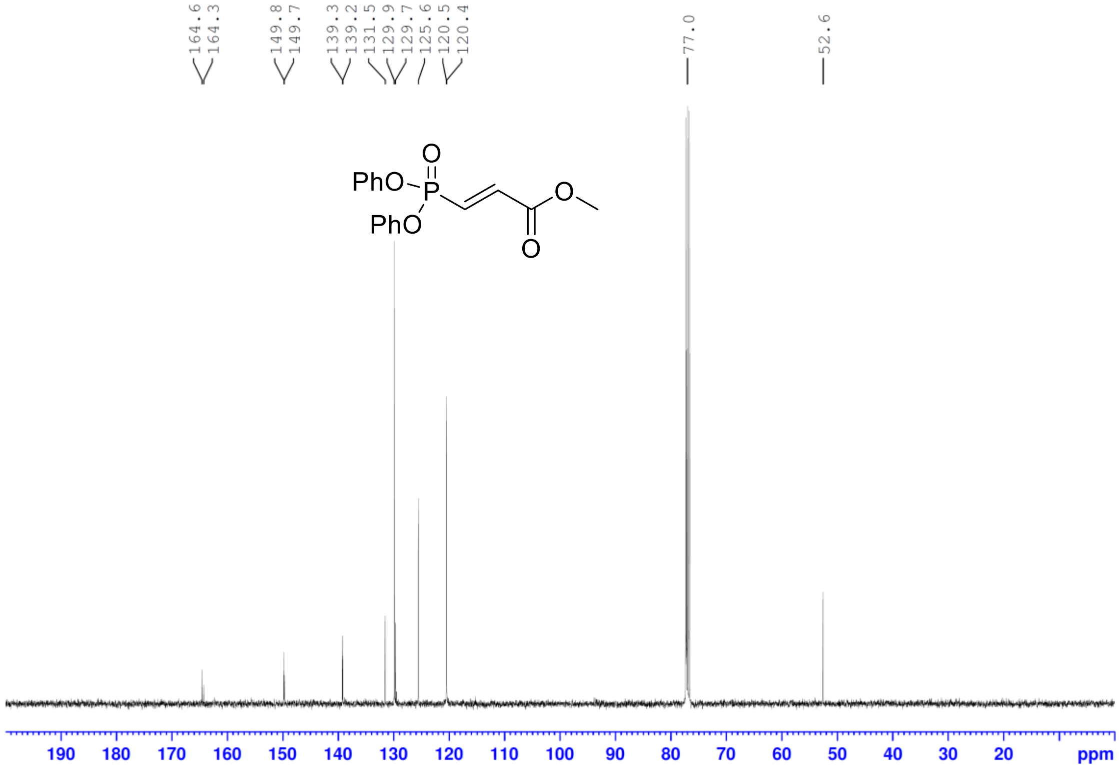

$^{13}C\{^{1}H\}$ NMR (100 MHz, $CDCl_3$) of compound **3aa (*E*)**

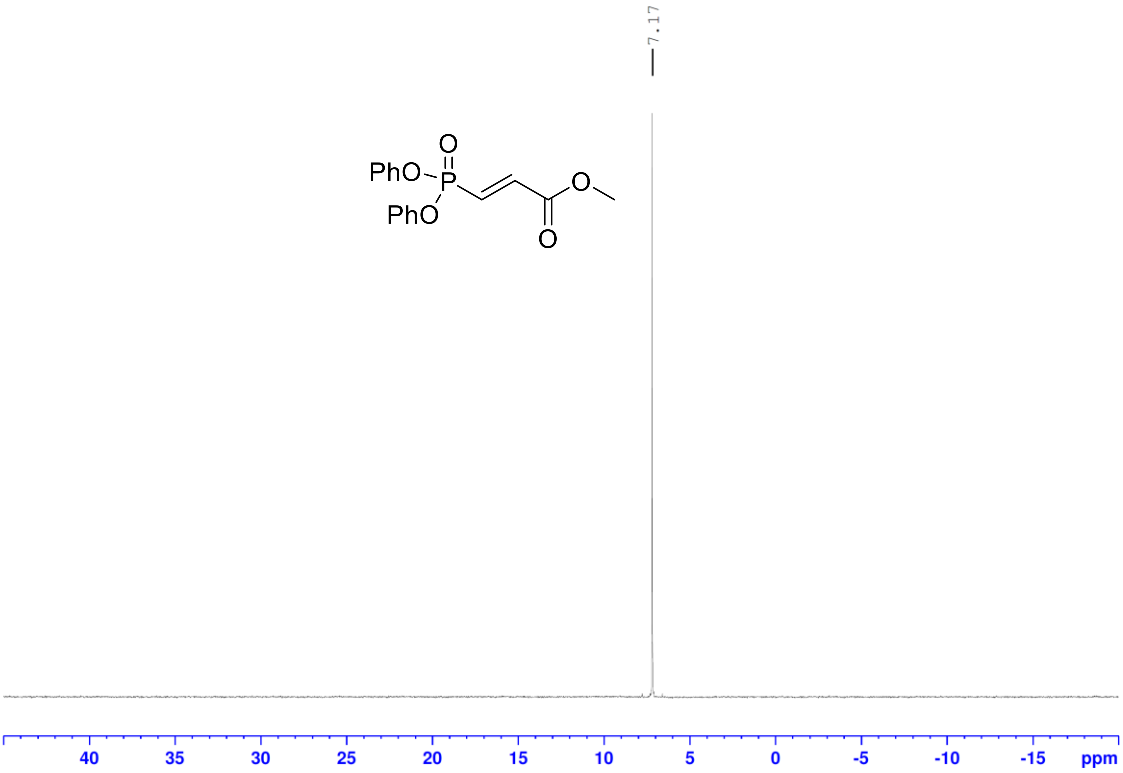

$^{31}P\{^{1}H\}$ NMR (162 MHz, $CDCl_3$) of compound **3aa (*E*)**

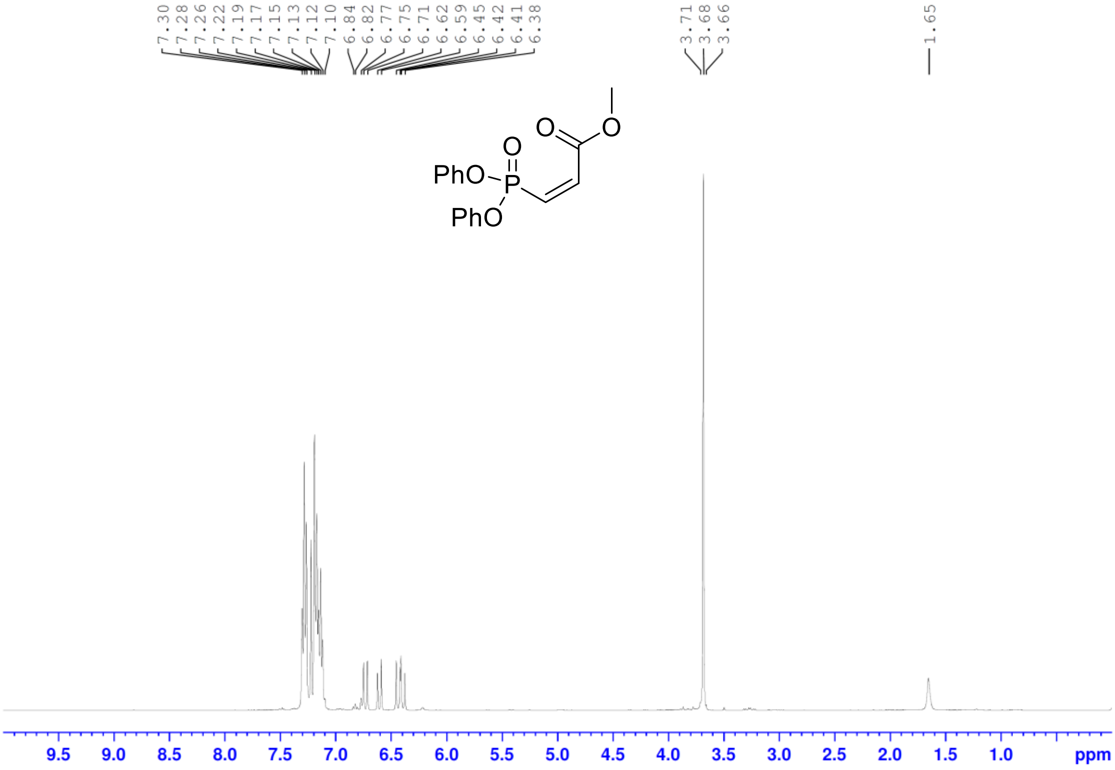

^{1}H NMR (400 MHz, $CDCl_3$) of compound **3aa (*Z*)**

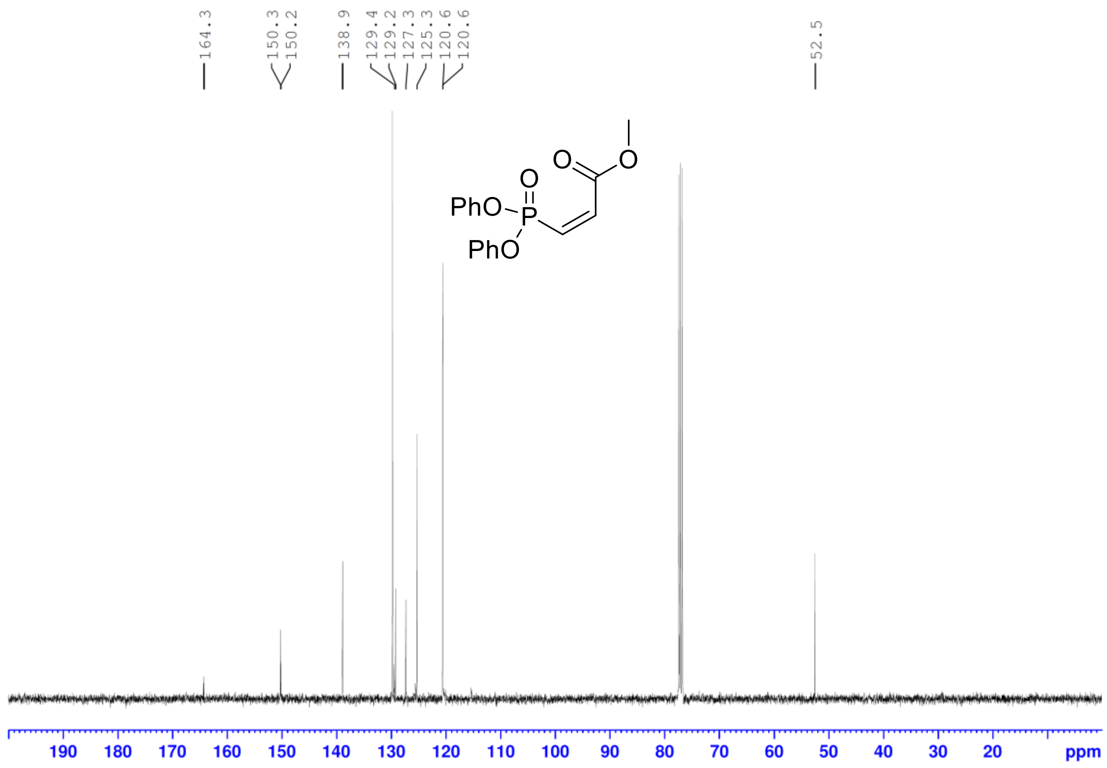

$^{13}C\{^{1}H\}$ NMR (100 MHz, $CDCl_3$) of compound **3aa (*Z*)**

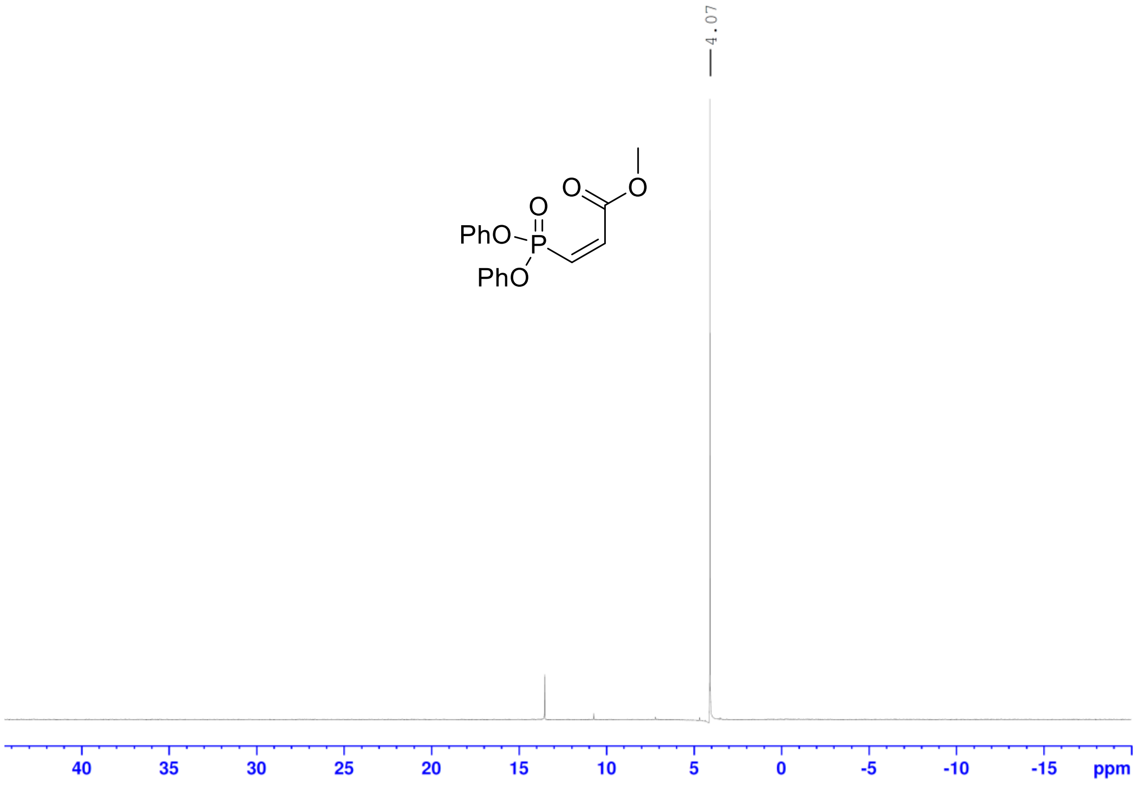

$^{31}P\{^{1}H\}$ NMR (162 MHz, $CDCl_3$) of compound **3aa (*Z*)**

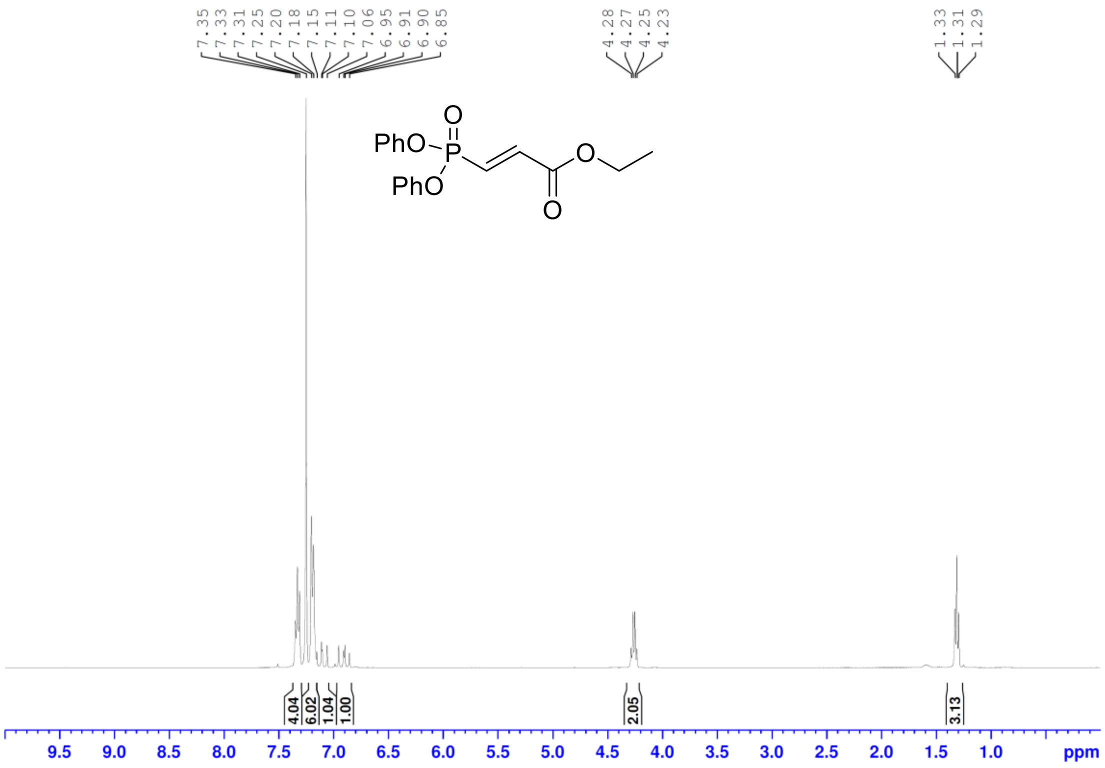

^{1}H NMR (400 MHz, $CDCl_3$) of compound **3ba (*E*)**

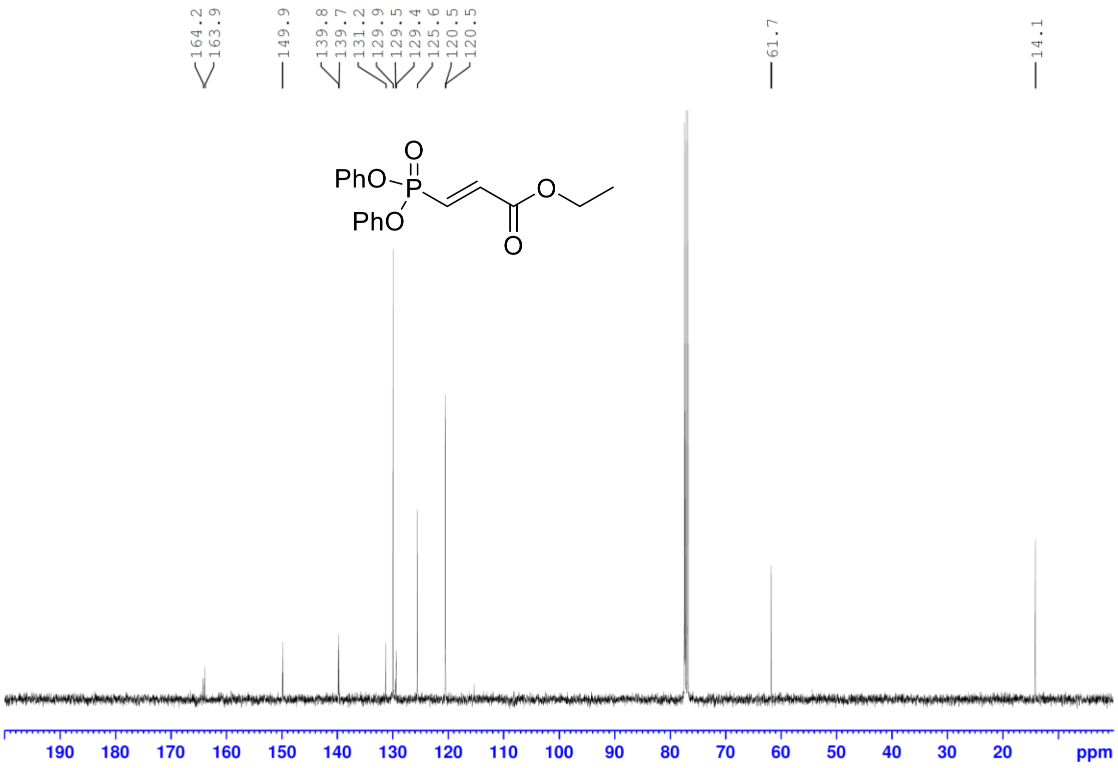

^{13}C{^{1}H} NMR (100 MHz, $CDCl_3$) of compound **3ba (*E*)**

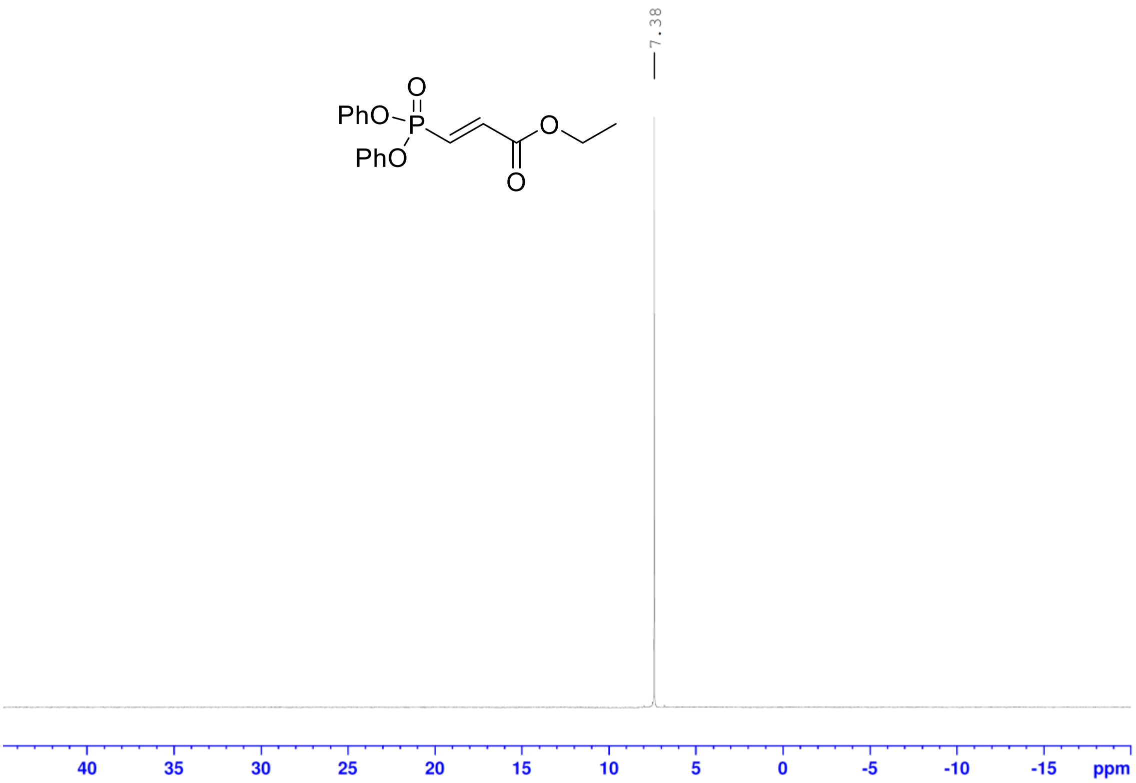

$^{31}P\{^{1}H\}$ NMR (162 MHz, $CDCl_3$) of compound **3ba** (***E***)

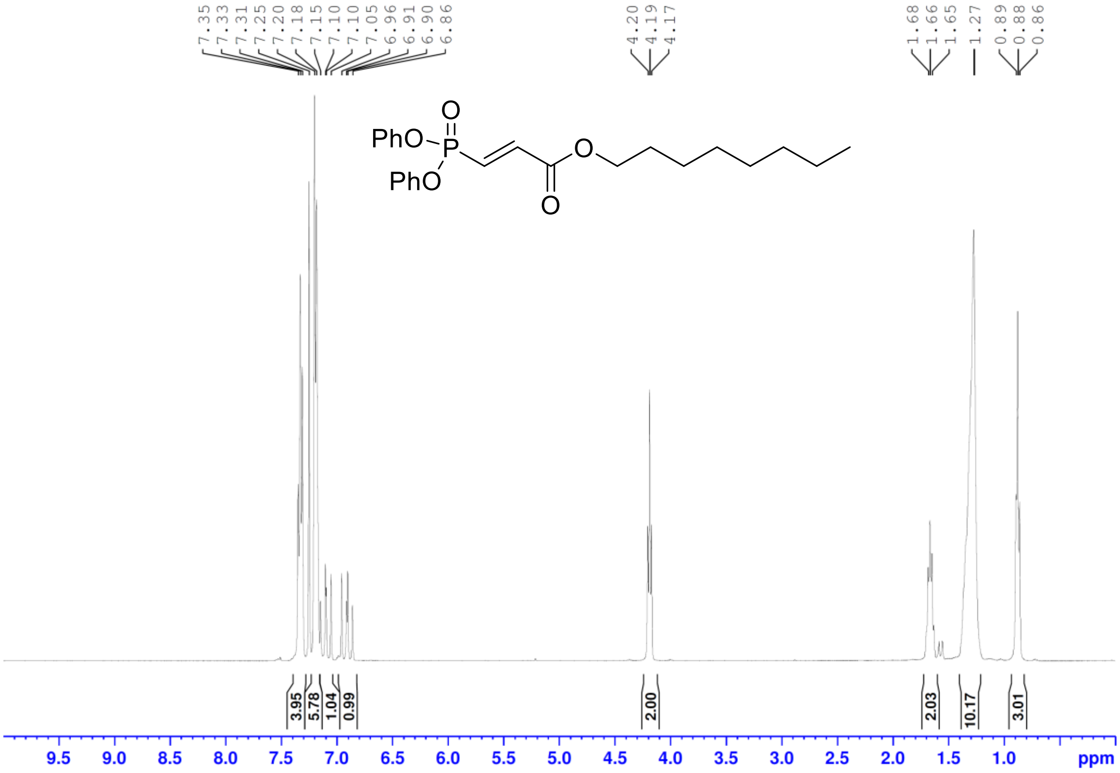

^{1}H NMR (400 MHz, $CDCl_3$) of compound **3ca** (***E***)

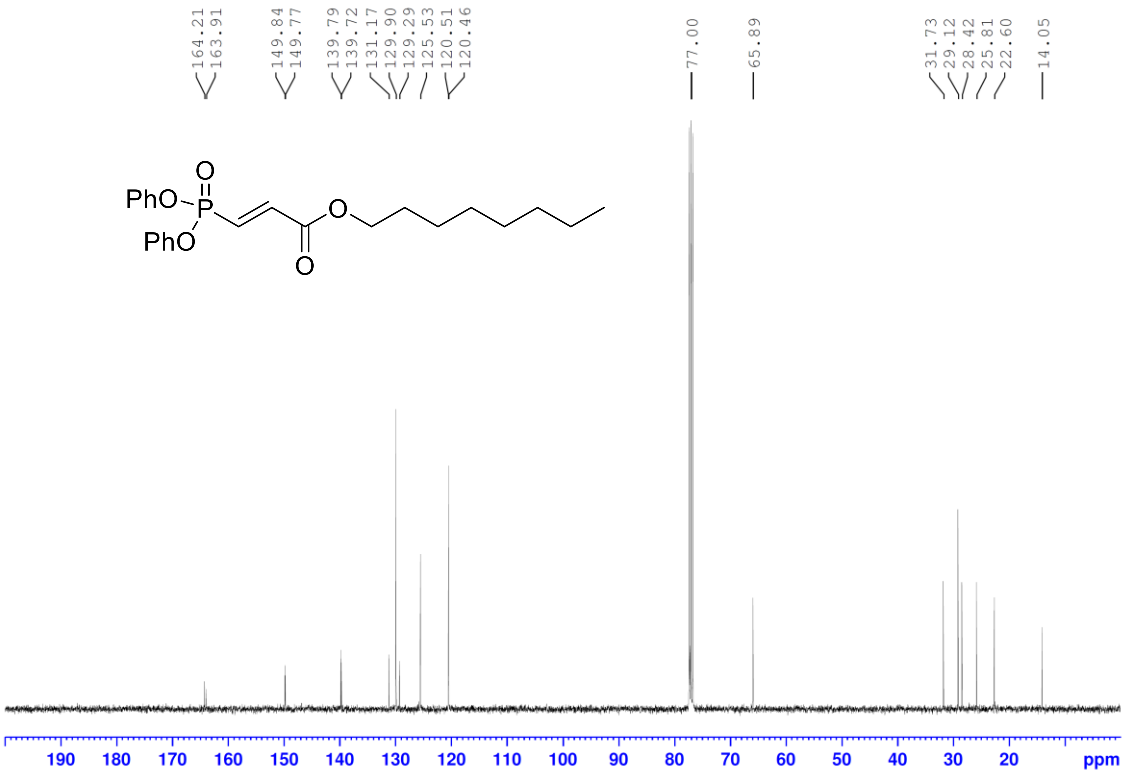

$^{13}C\{^{1}H\}$ NMR (100 MHz, $CDCl_3$) of compound **3ca** (***E***)

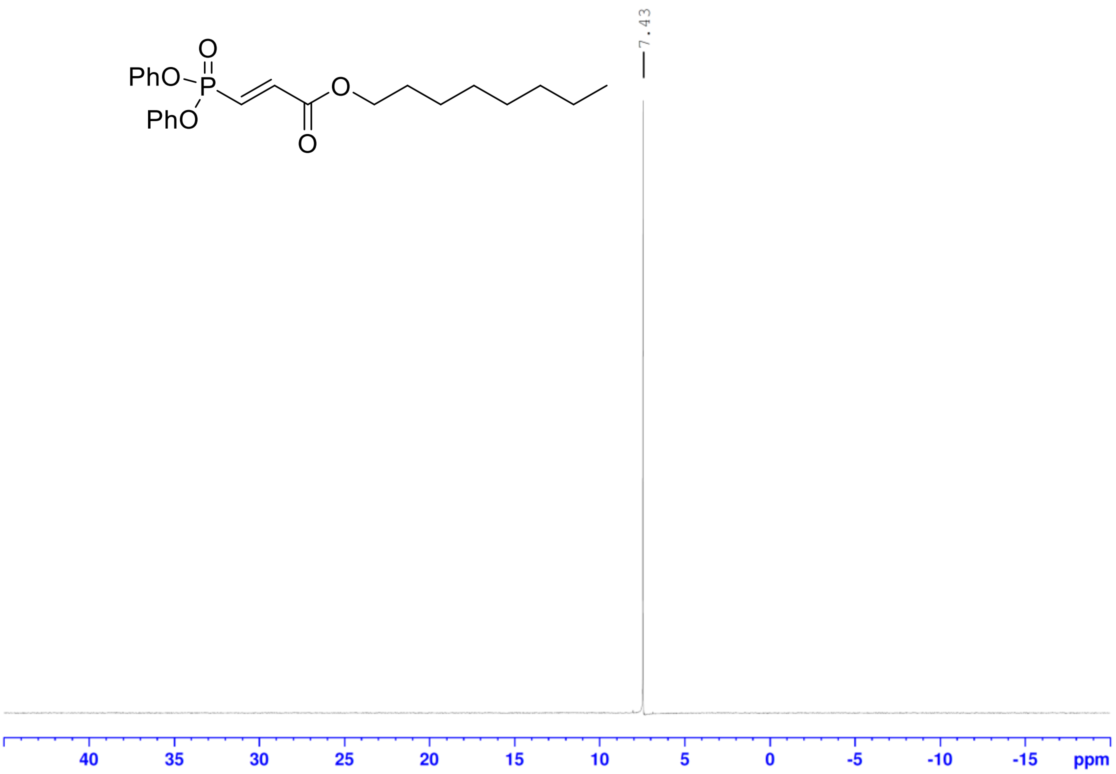

$^{31}P\{^{1}H\}$ NMR (162 MHz, $CDCl_3$) of compound **3ca** (***E***)

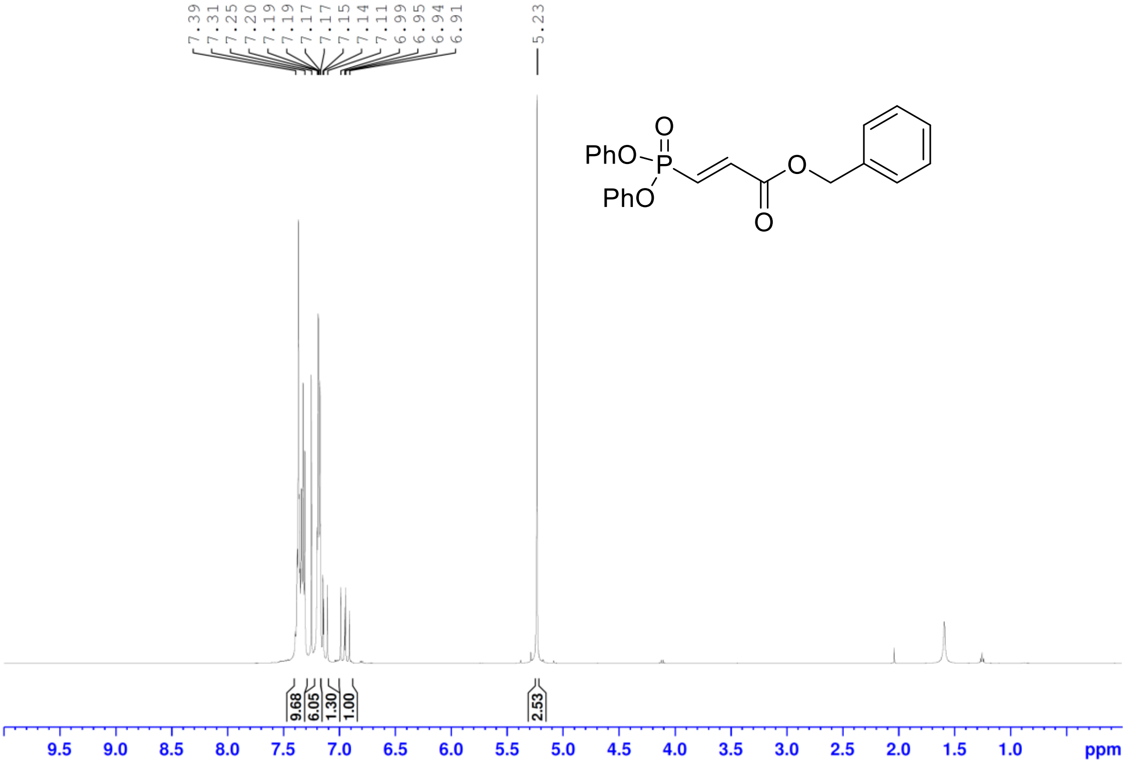

^{1}H NMR (500 MHz, $CDCl_3$) of compound **3da (*E*)**

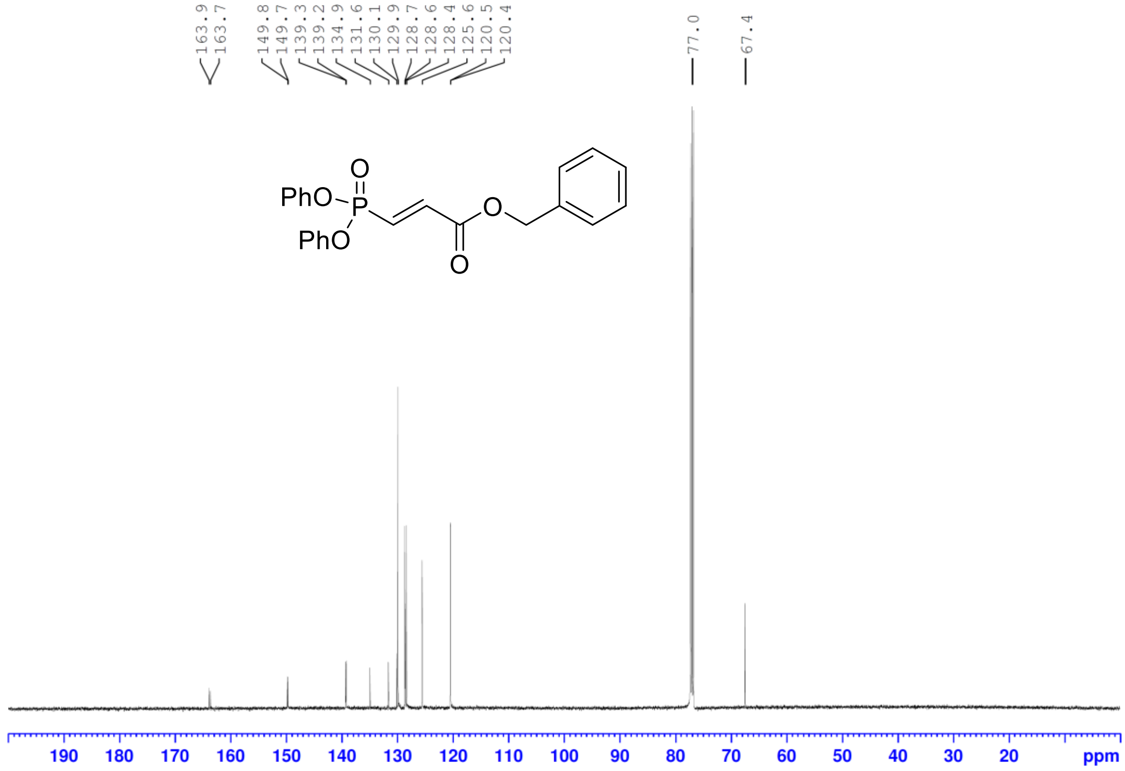

^{13}C{^{1}H} NMR (125 MHz, $CDCl_3$) of compound **3da (*E*)**

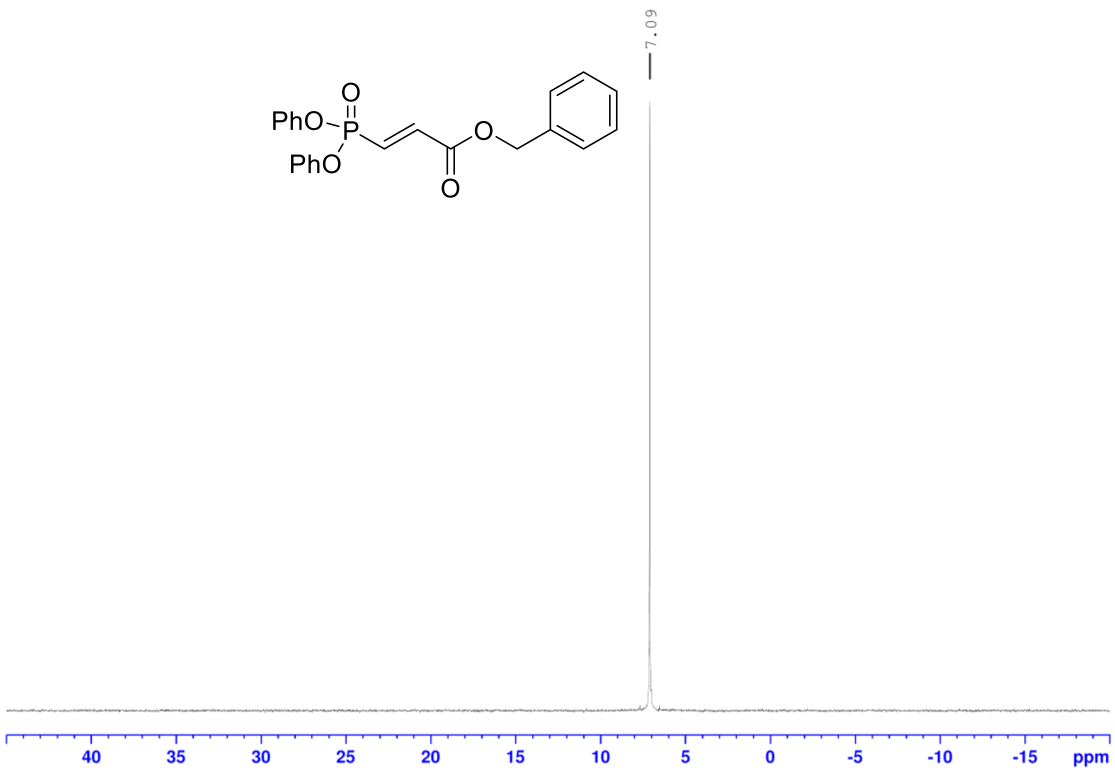

$^{31}P\{^1H\}$ NMR (162 MHz, $CDCl_3$) of compound **3da (*E*)**

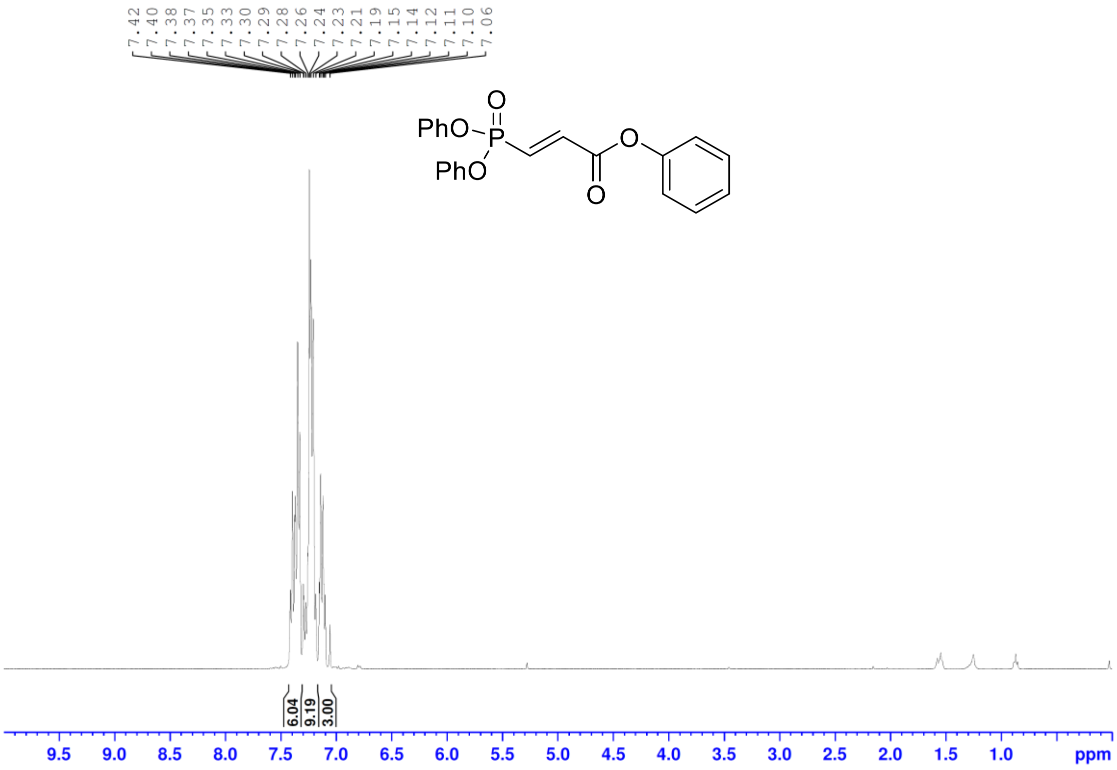

^1H NMR (400 MHz, $CDCl_3$) of compound **3ea (*E*)**

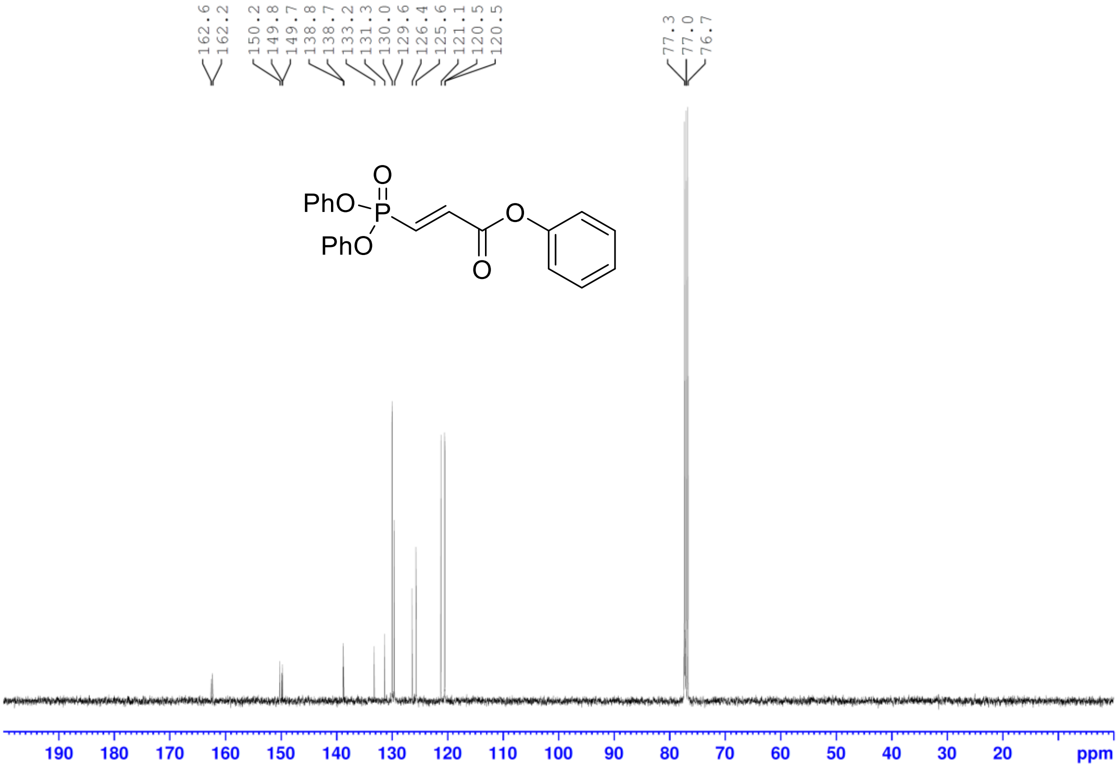

$^{13}C\{^{1}H\}$ NMR (100 MHz, $CDCl_3$) of compound **3ea (*E*)**

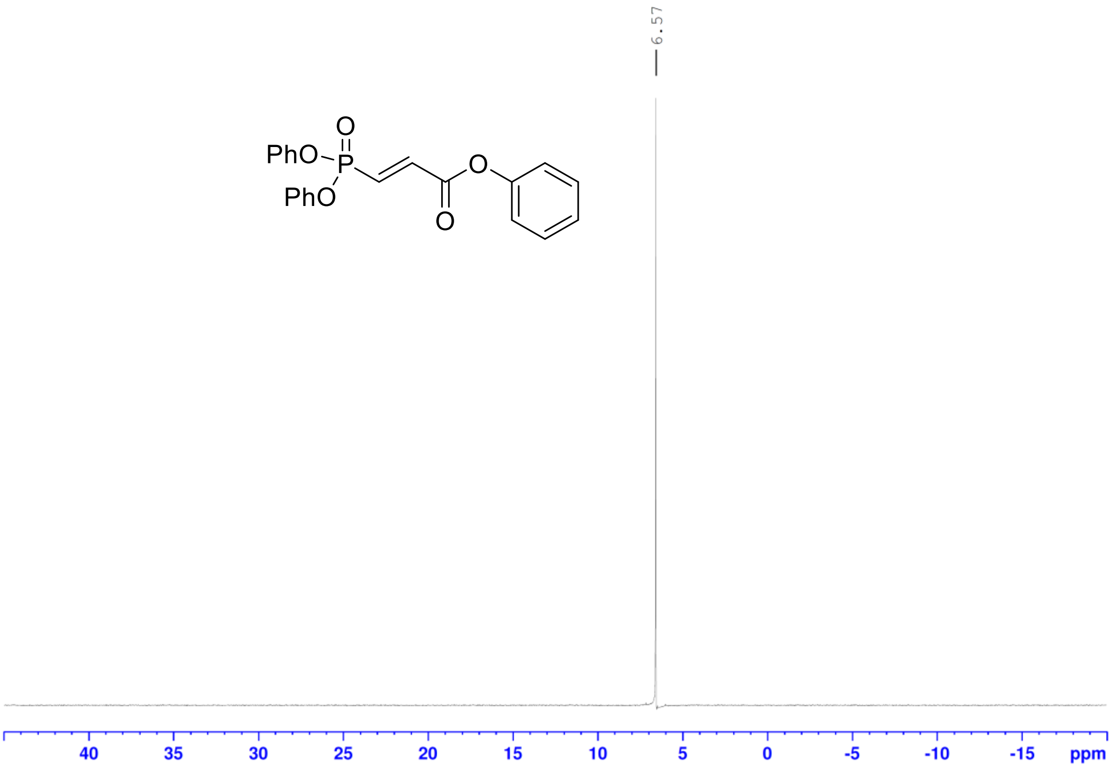

$^{31}P\{^{1}H\}$ NMR (162 MHz, $CDCl_3$) of compound **3ea (*E*)**

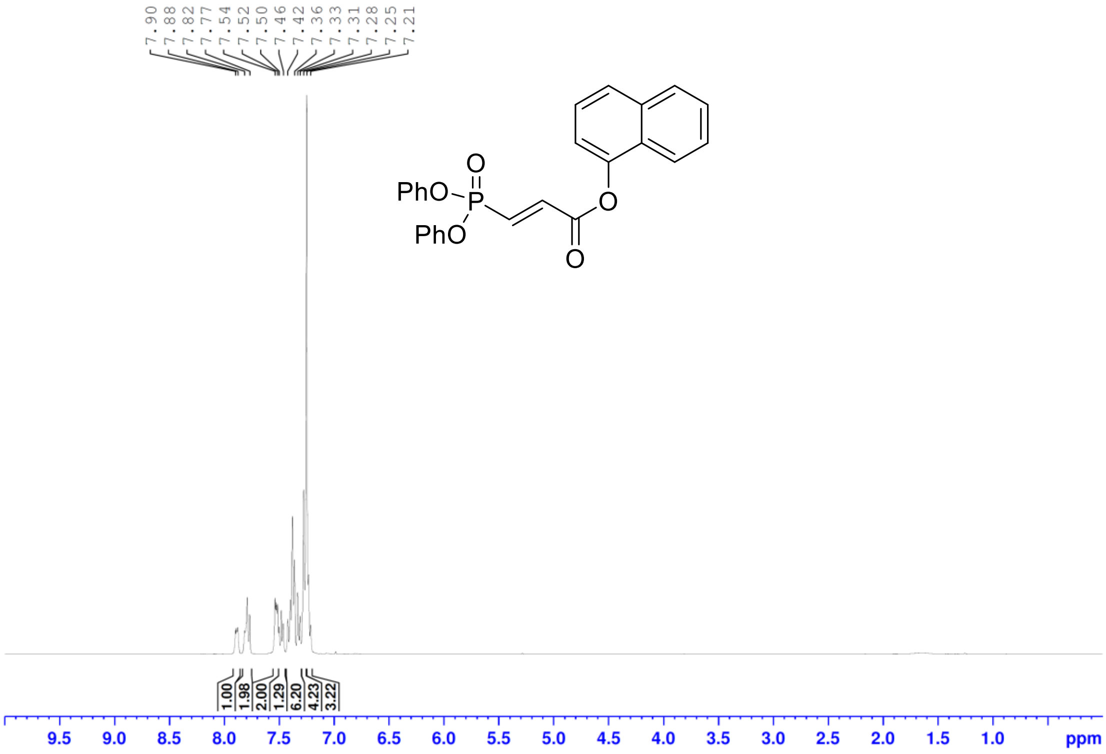

^{1}H NMR (400 MHz, $CDCl_3$) of compound **3fa** (***E***)

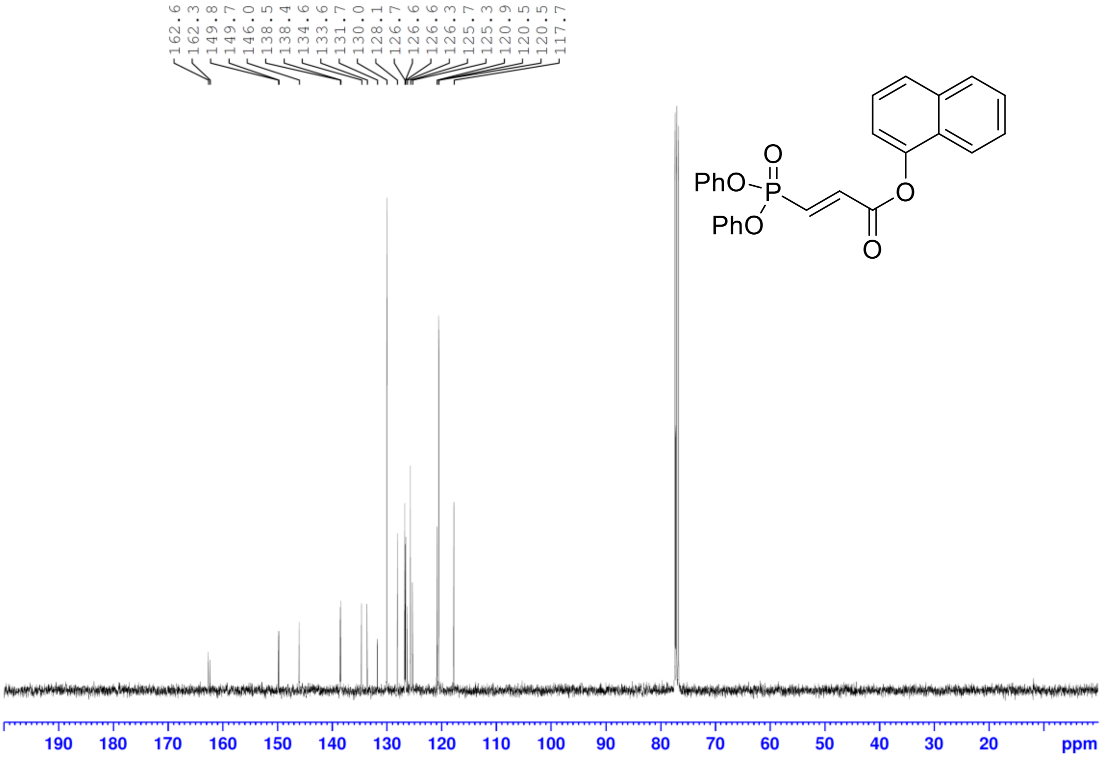

^{13}C{^{1}H} NMR (100 MHz, $CDCl_3$) of compound **3fa** (***E***)

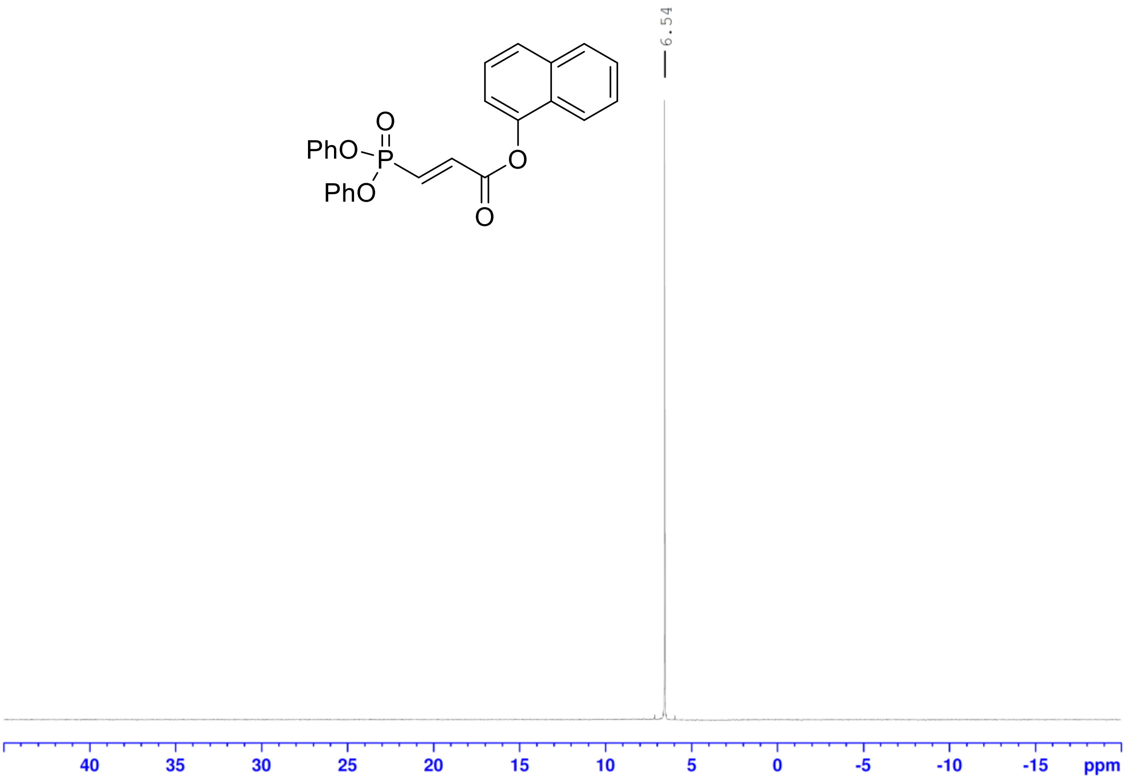

$^{31}P\{^1H\}$ NMR (162 MHz, $CDCl_3$) of compound **3fa (*E*)**

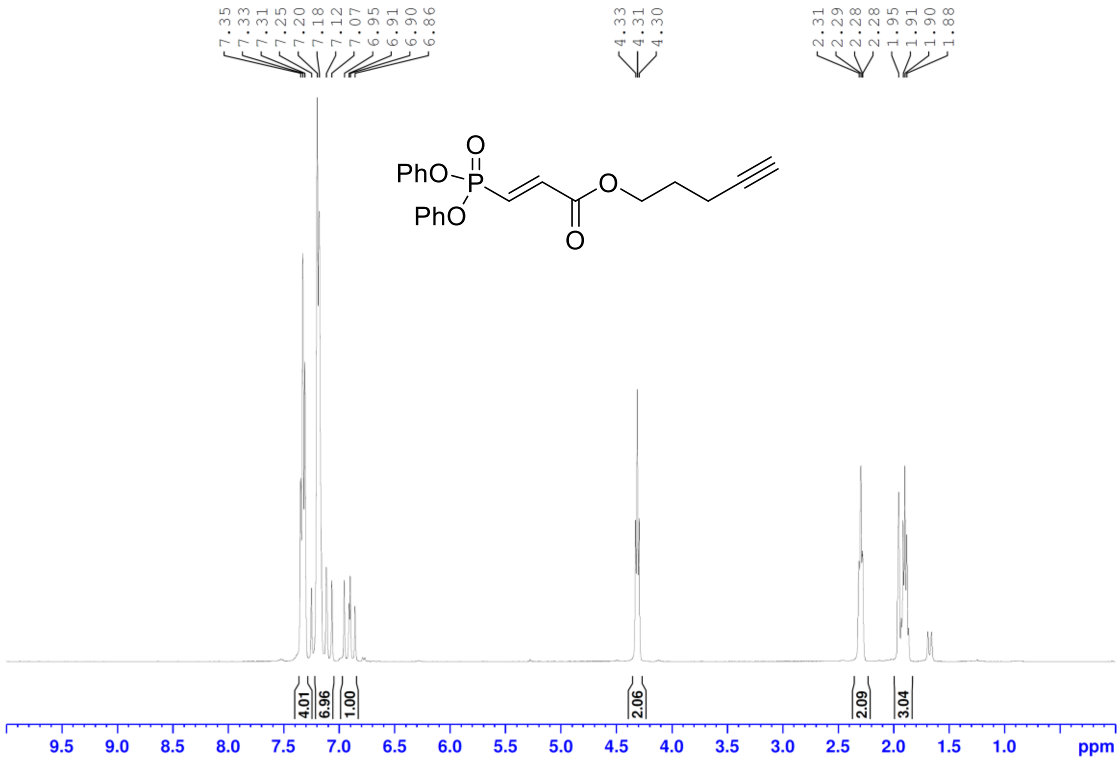

^1H NMR (400 MHz, $CDCl_3$) of compound **3ga (*E*)**

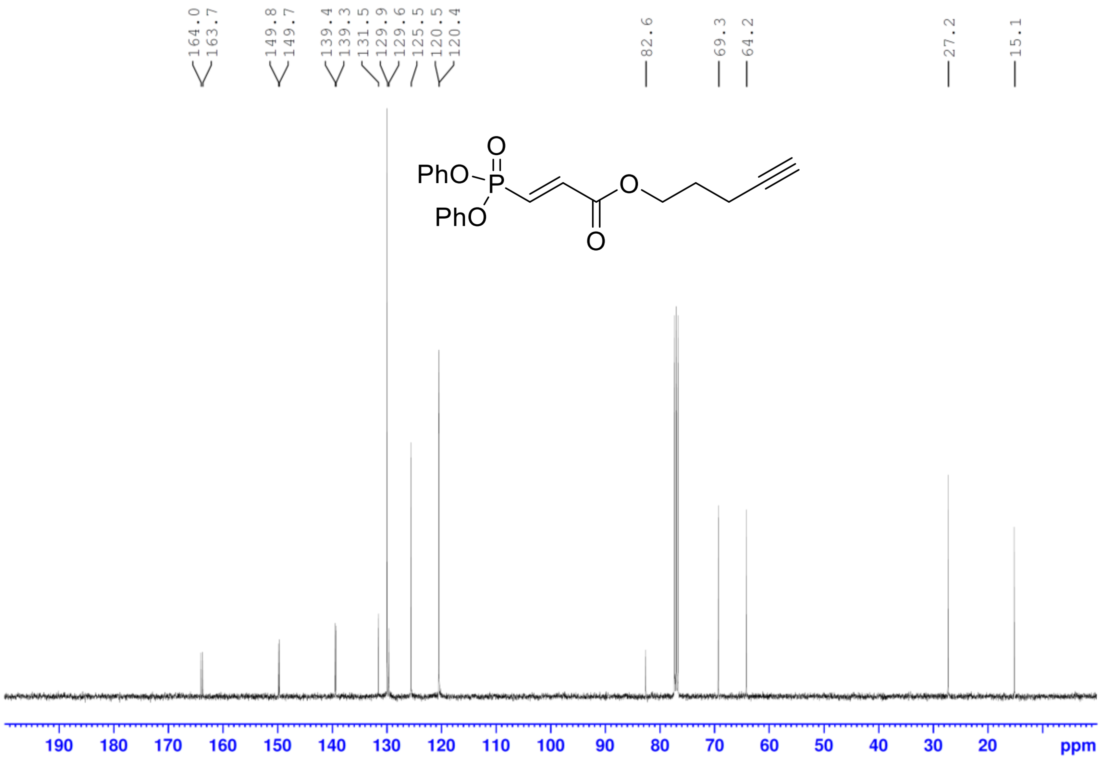

$^{13}C\{^{1}H\}$ NMR (100 MHz, $CDCl_3$) of compound **3ga** (***E***)

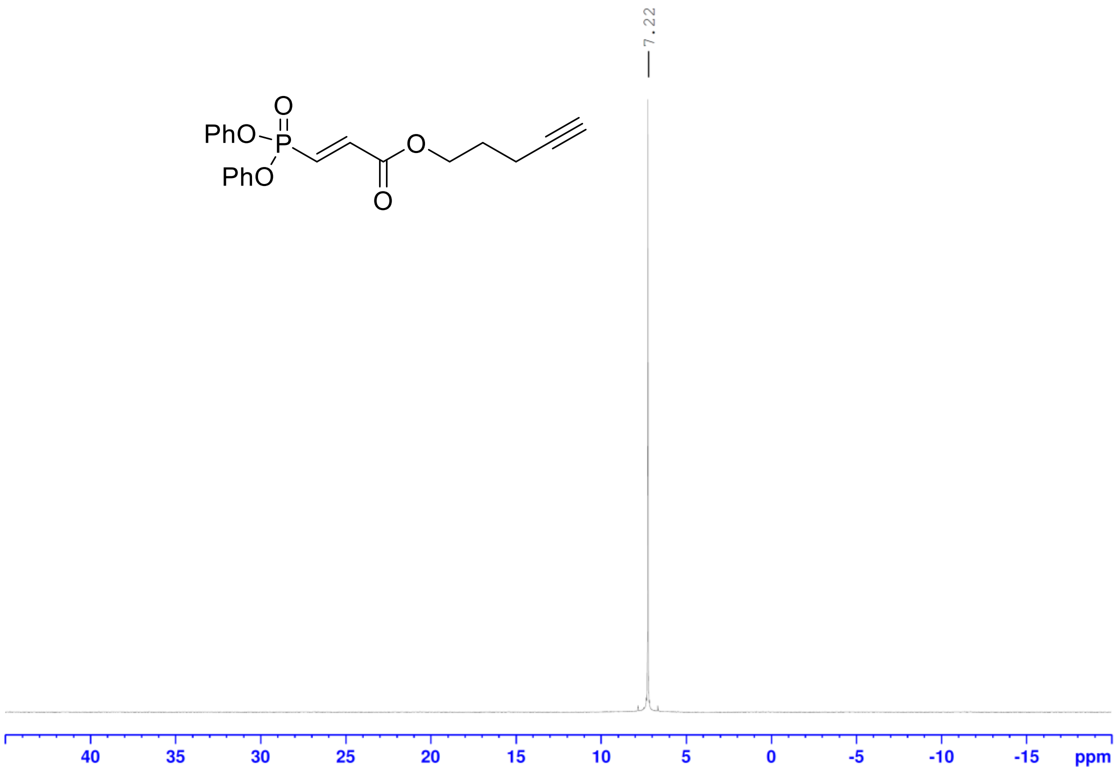

$^{31}P\{^{1}H\}$ NMR (162 MHz, $CDCl_3$) of compound **3ga** (***E***)

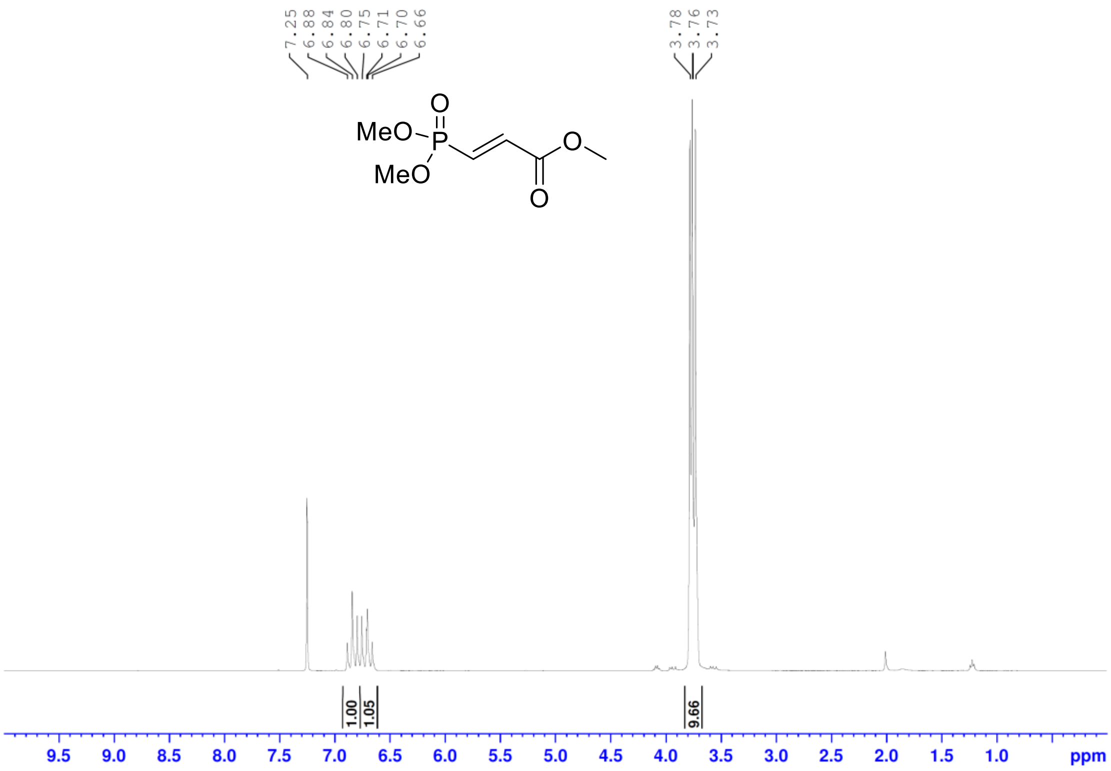

^{1}H NMR (400 MHz, $CDCl_3$) of compound **3ab (*E*)**

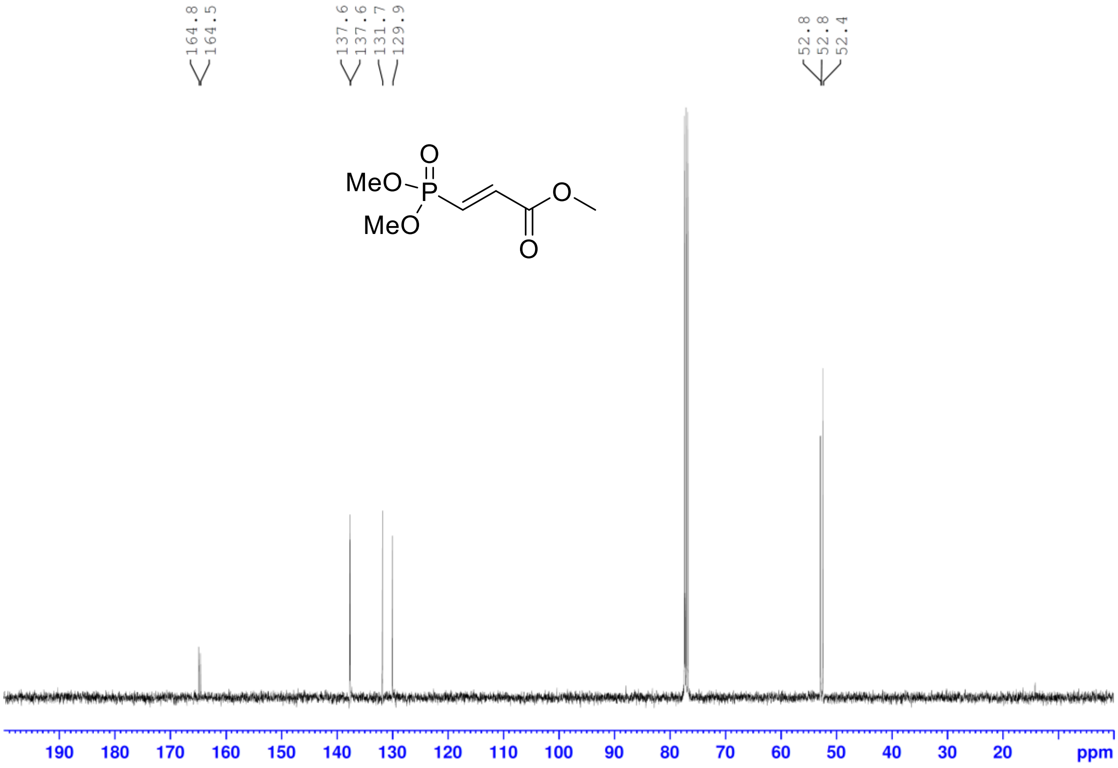

$^{13}C\{^1H\}$ NMR (100 MHz, $CDCl_3$) of compound **3ab (*E*)**

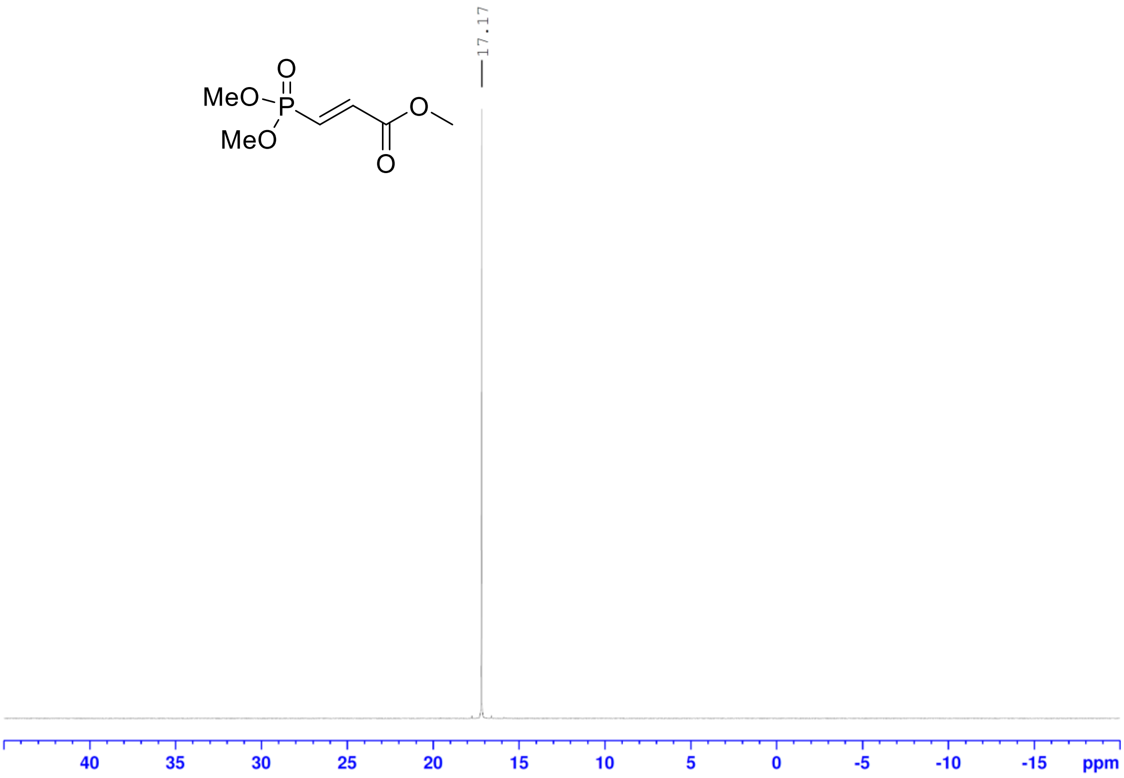

$^{31}P\{^{1}H\}$ NMR (162 MHz, $CDCl_3$) of compound **3ab (*E*)**

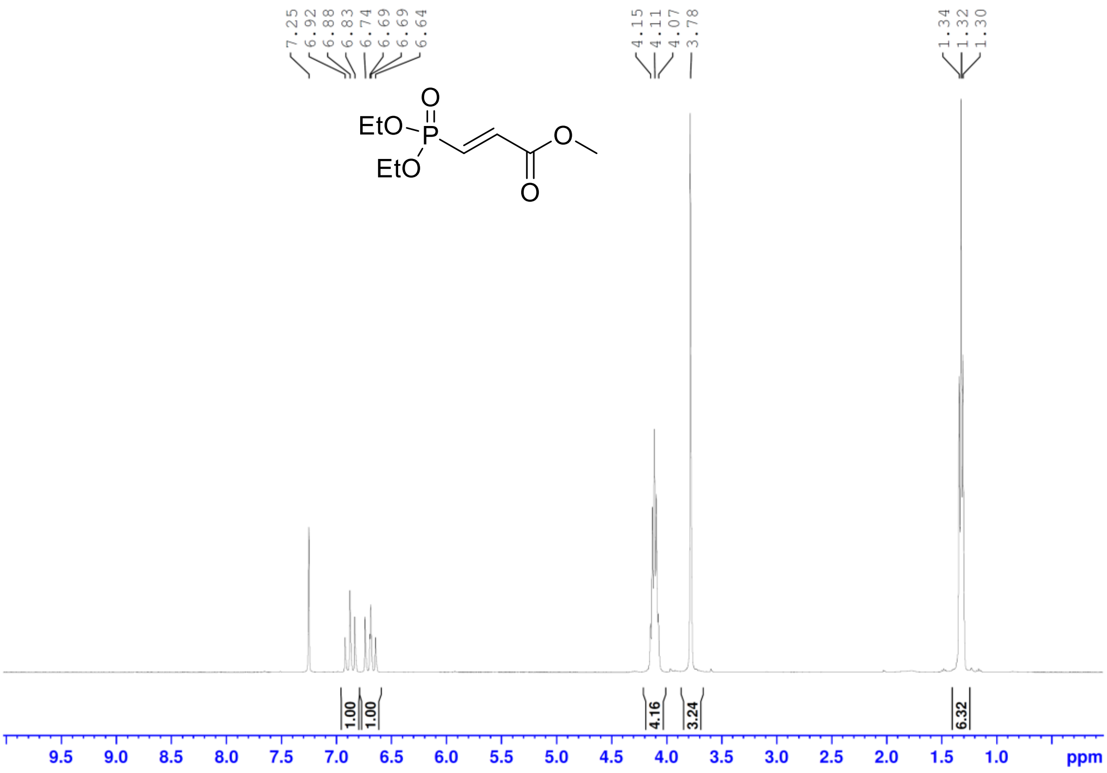

^{1}H NMR (400 MHz, $CDCl_3$) of compound **3ac (*E*)**

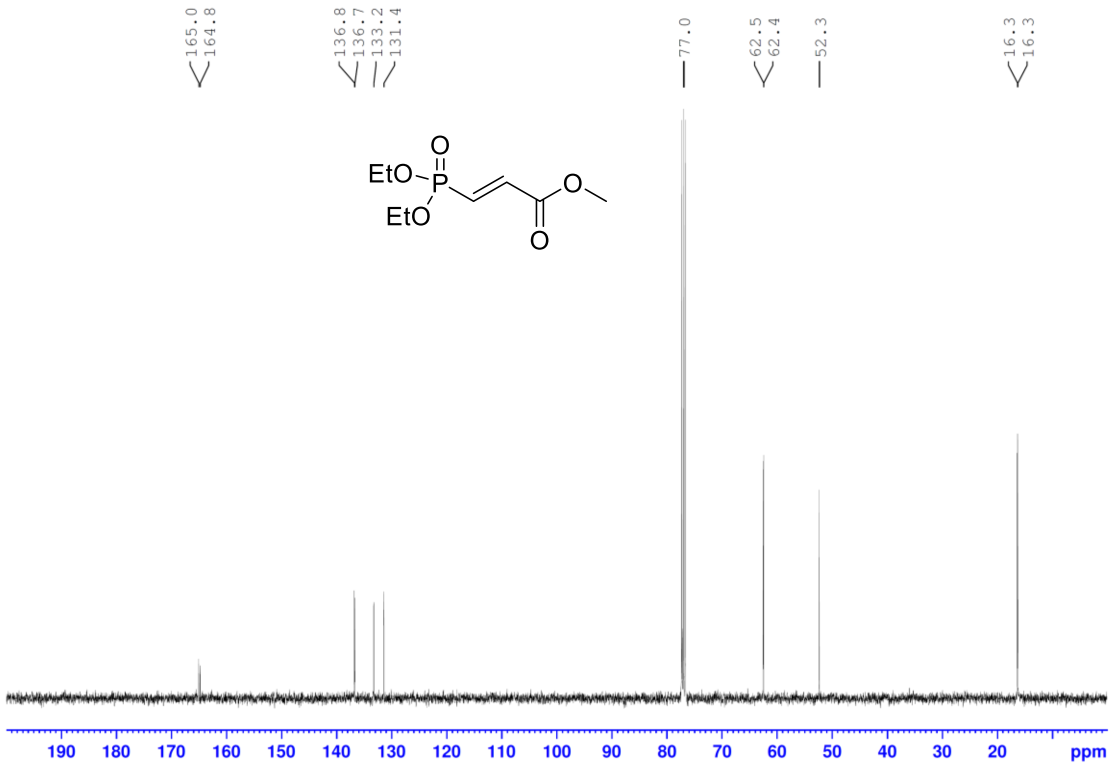

$^{13}C\{^{1}H\}$ NMR (100 MHz, $CDCl_3$) of compound **3ac** (***E***)

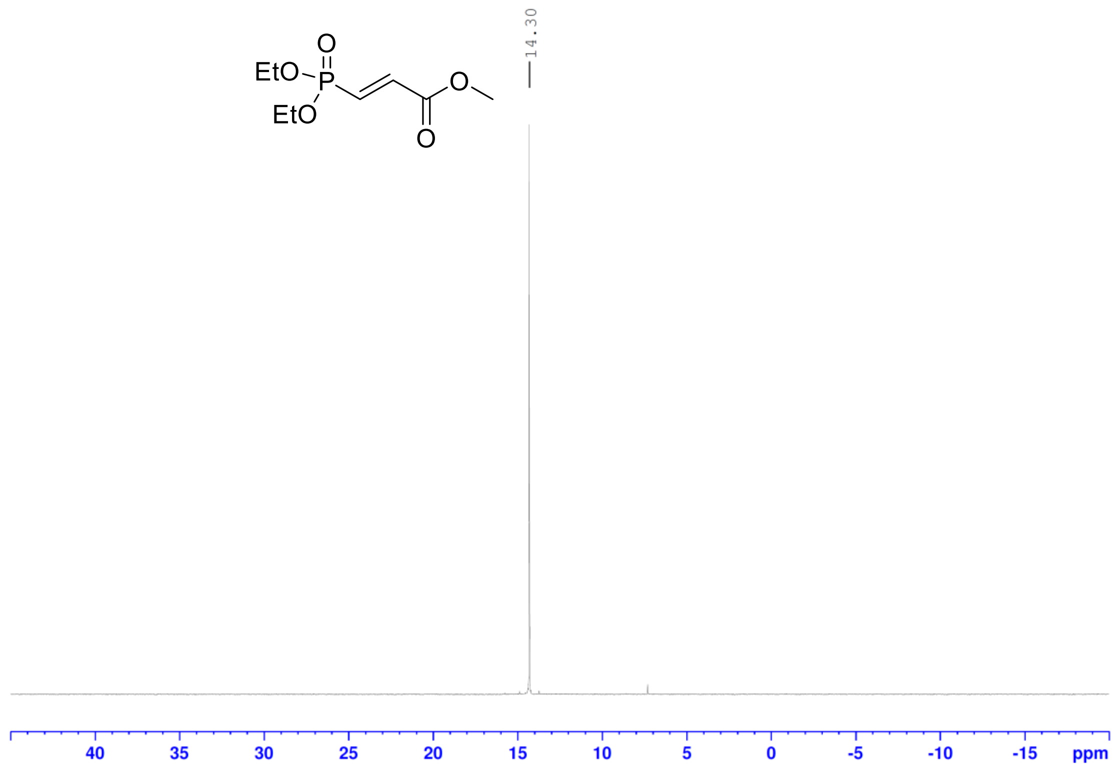

$^{31}P\{^{1}H\}$ NMR (162 MHz, $CDCl_3$) of compound **3ac** (***E***)

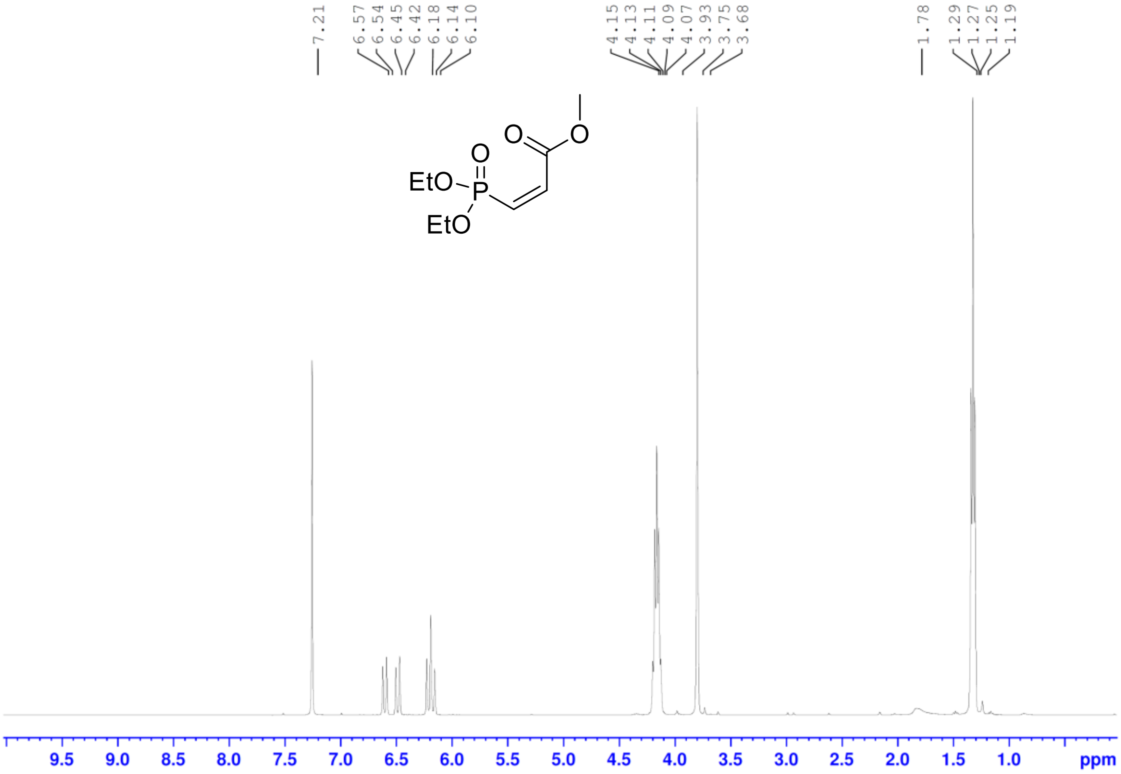

^{1}H NMR (400 MHz, $CDCl_3$) of compound **3ac (*Z*)**

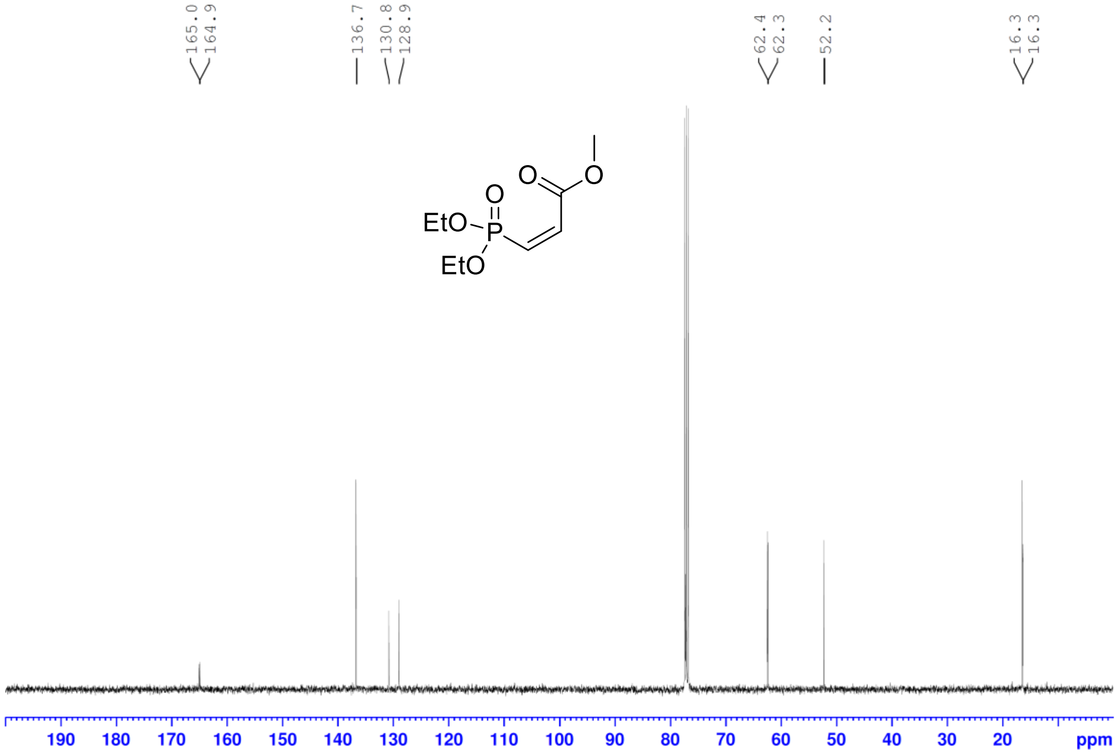

$^{13}C\{^{1}H\}$ NMR (100 MHz, $CDCl_3$) of compound **3ac (*Z*)**

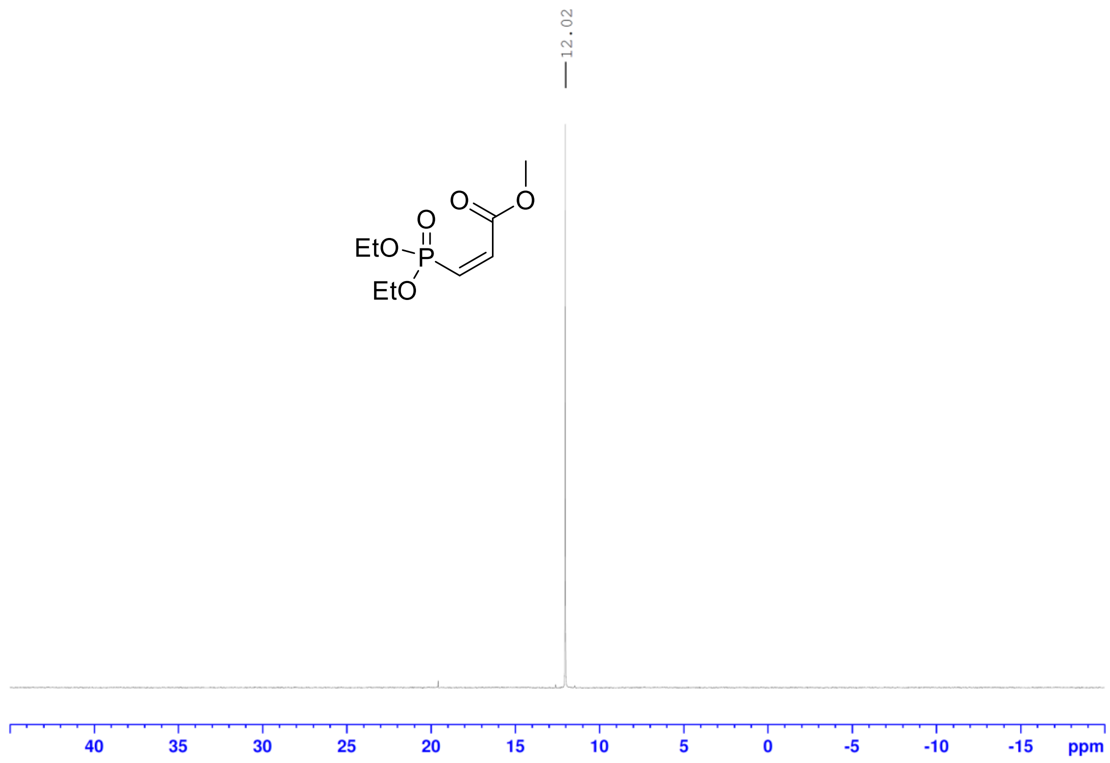

$^{31}P\{^{1}H\}$ NMR (162 MHz, $CDCl_3$) of compound **3ac** (***Z***)

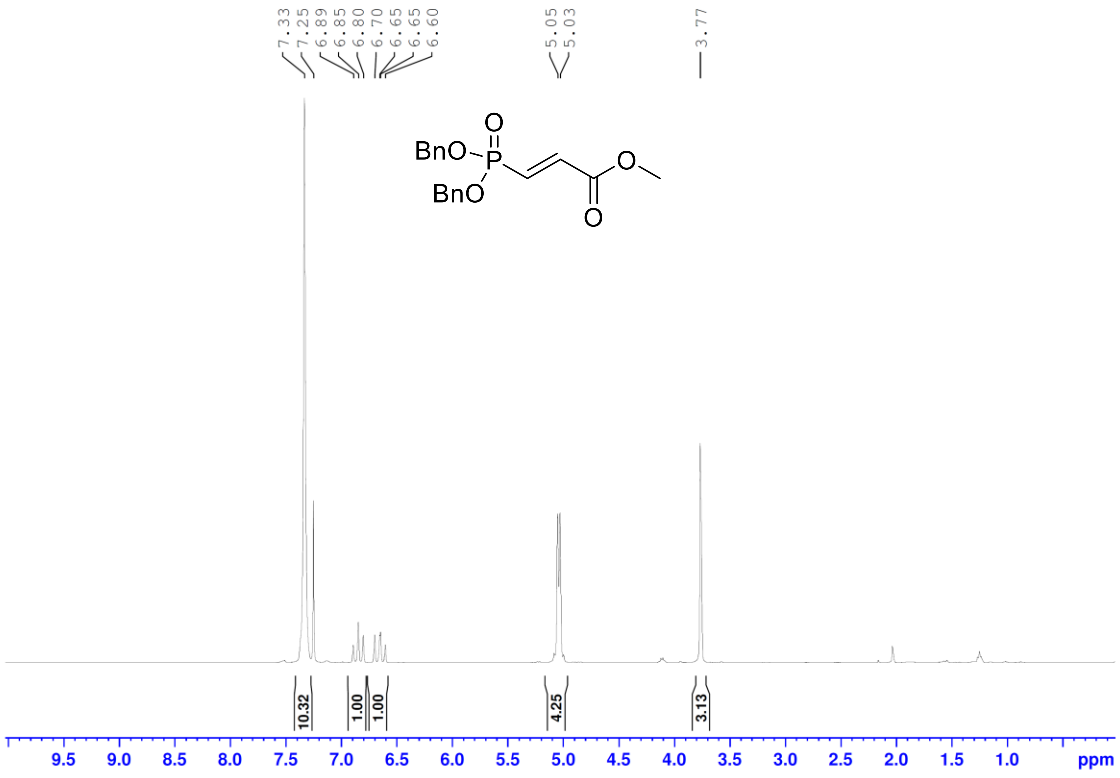

^{1}H NMR (400 MHz, $CDCl_3$) of compound **3ad** (***E***)

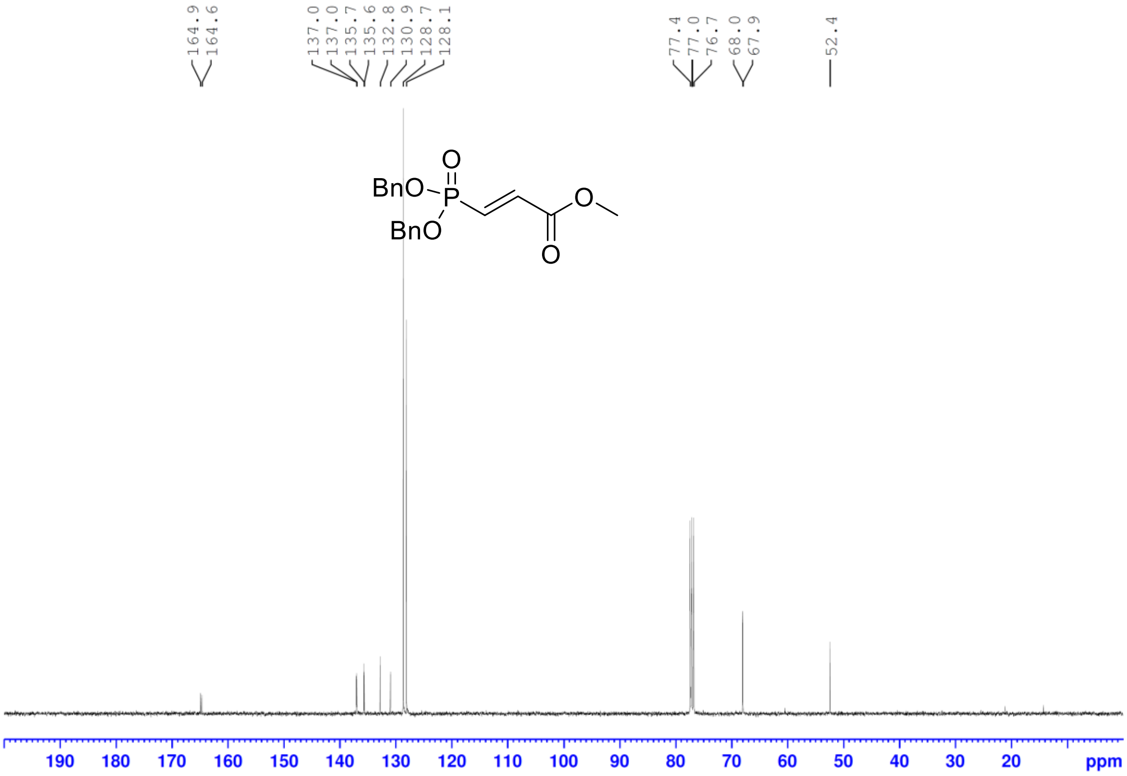

$^{13}C\{^{1}H\}$ NMR (100 MHz, $CDCl_3$) of compound **3ad (*E*)**

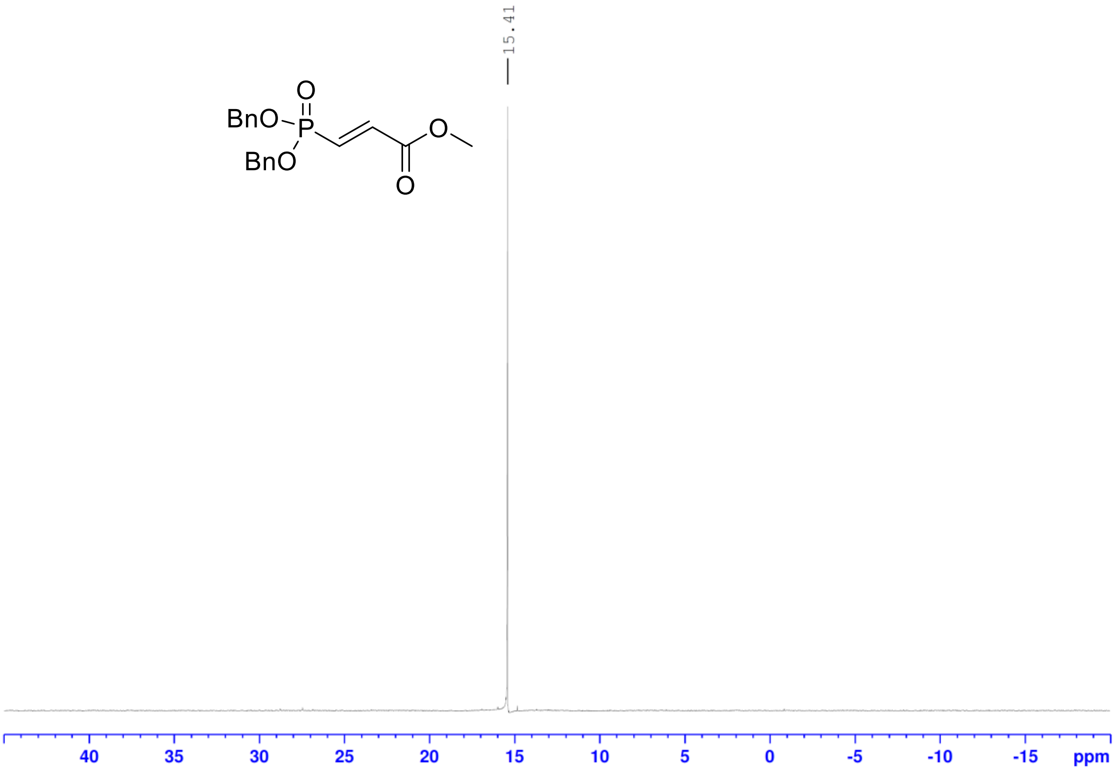

$^{31}P\{^{1}H\}$ NMR (162 MHz, $CDCl_3$) of compound **3ad (*E*)**

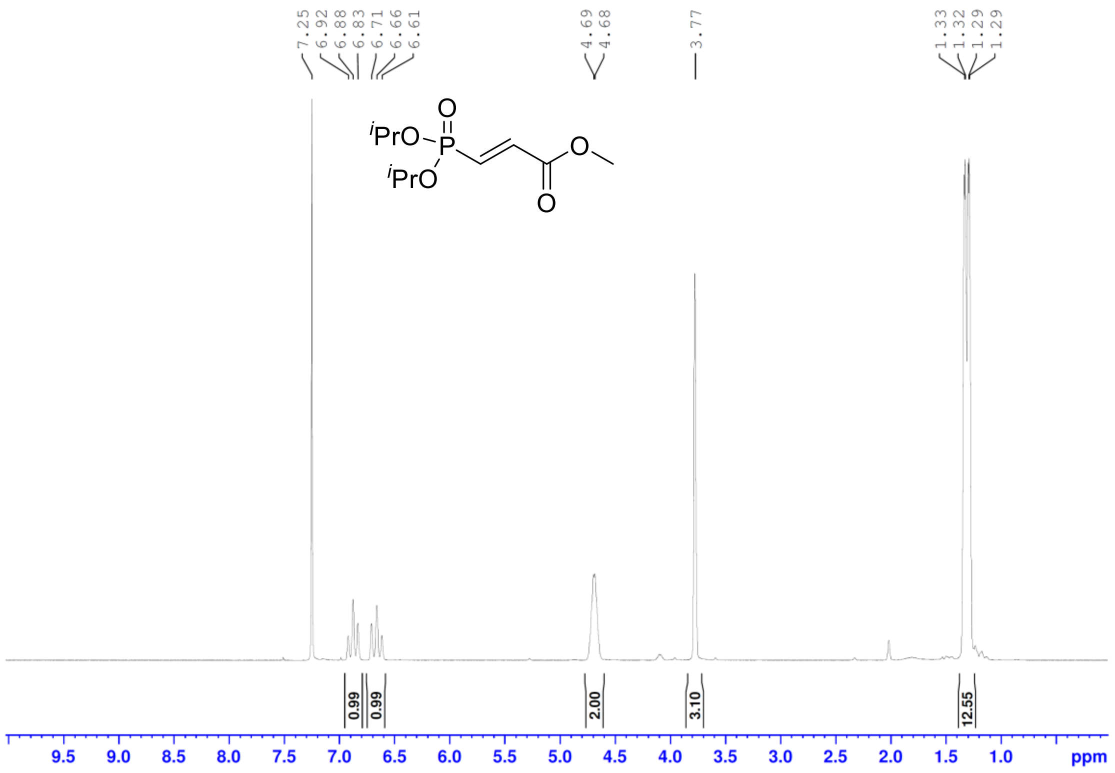

^{1}H NMR (400 MHz, $CDCl_3$) of compound **3ae** **(*E*)**

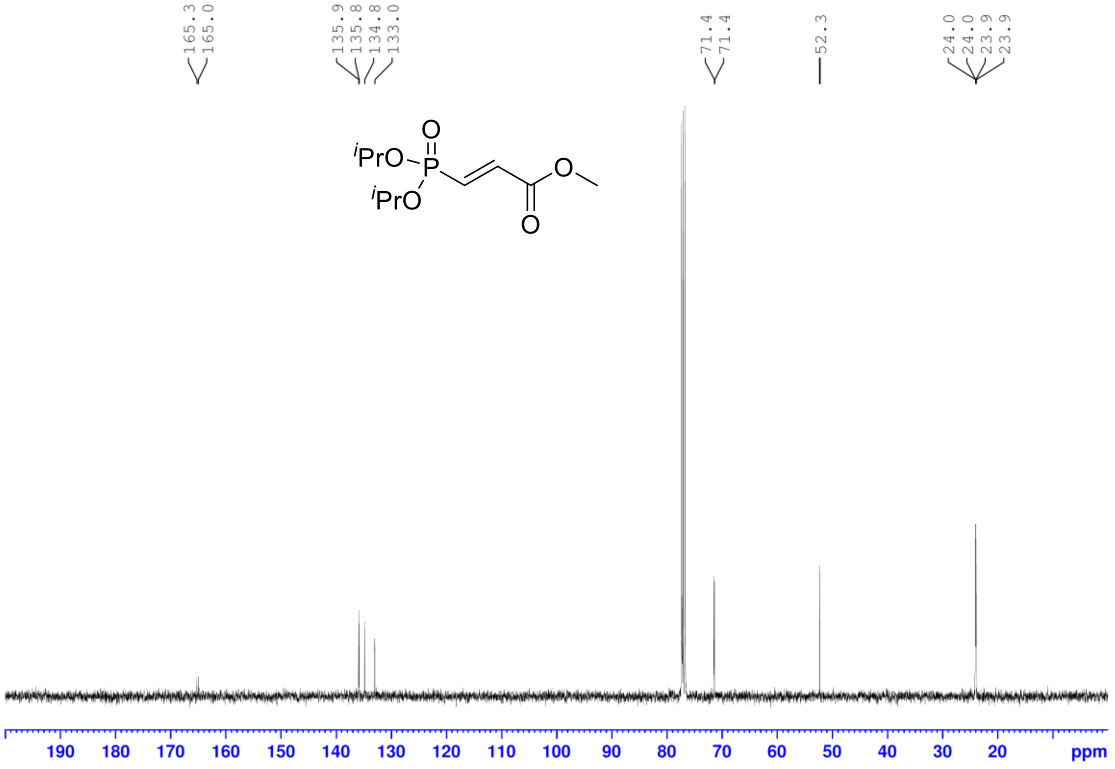

$^{13}C\{^1H\}$ NMR (100 MHz, $CDCl_3$) of compound **3ae** **(*E*)**

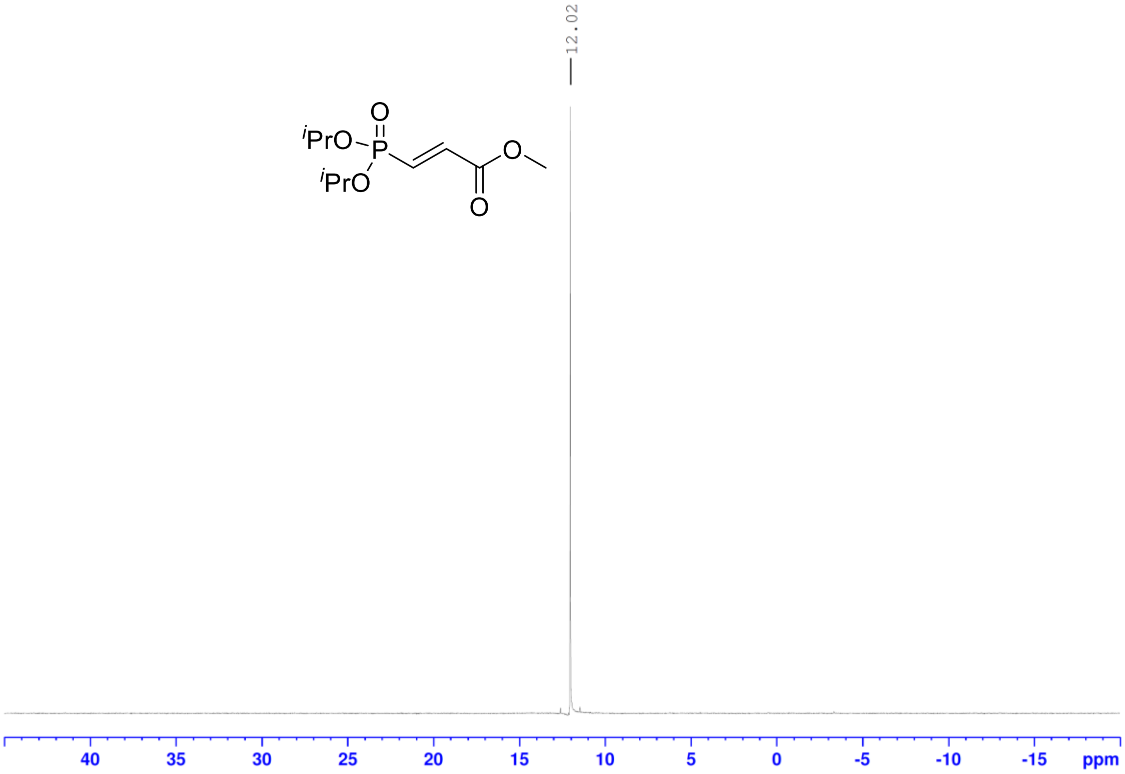

$^{31}P\{^{1}H\}$ NMR (162 MHz, $CDCl_3$) of compound **3ae (*E*)**

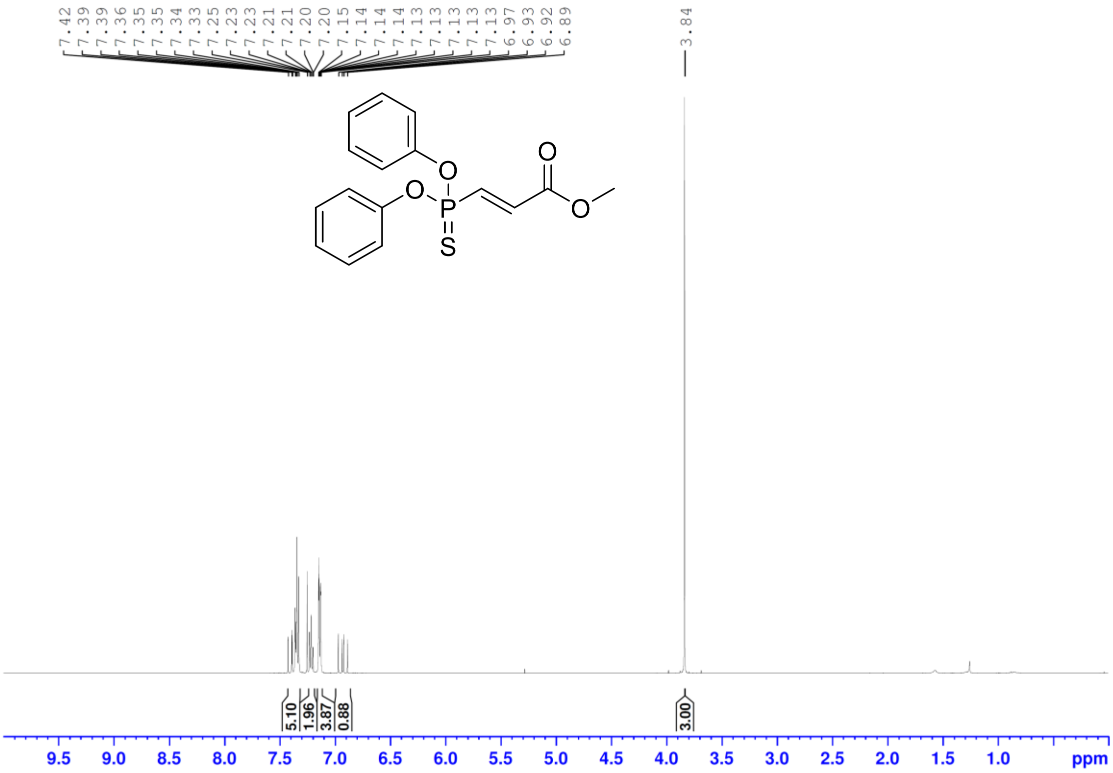

^{1}H NMR (500 MHz, $CDCl_3$) of compound **6a**

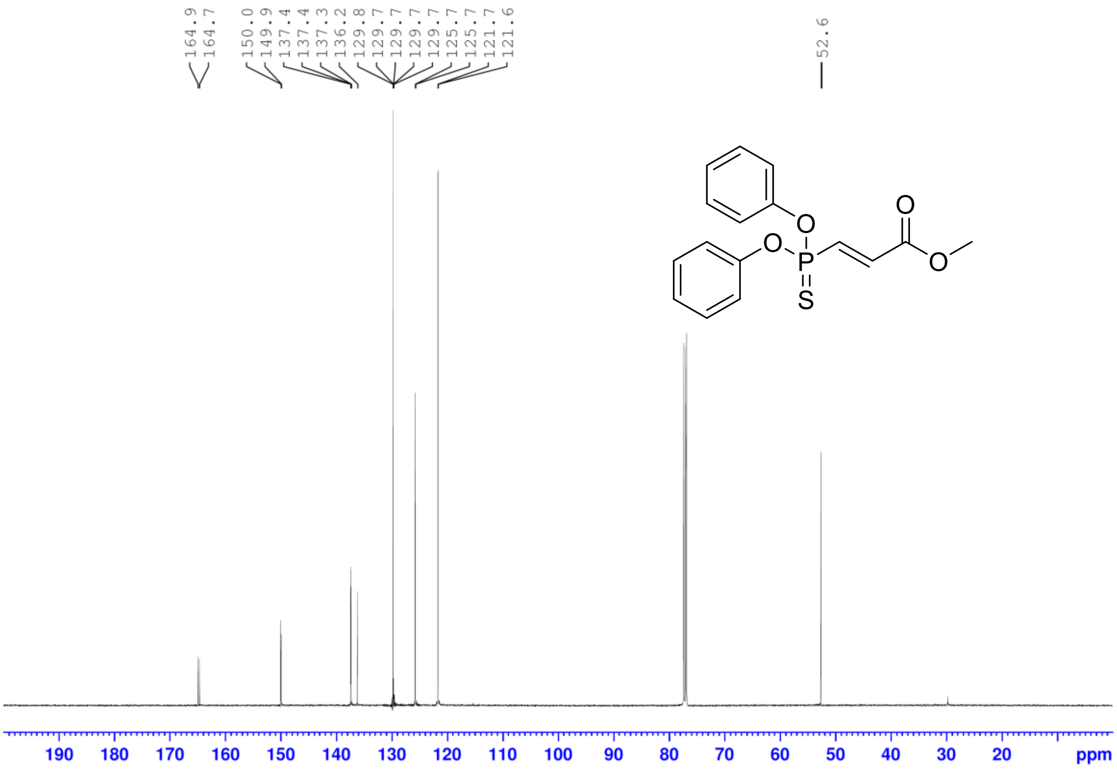

$^{13}C\{^{1}H\}$ NMR (125 MHz, $CDCl_3$) of compound **6a**

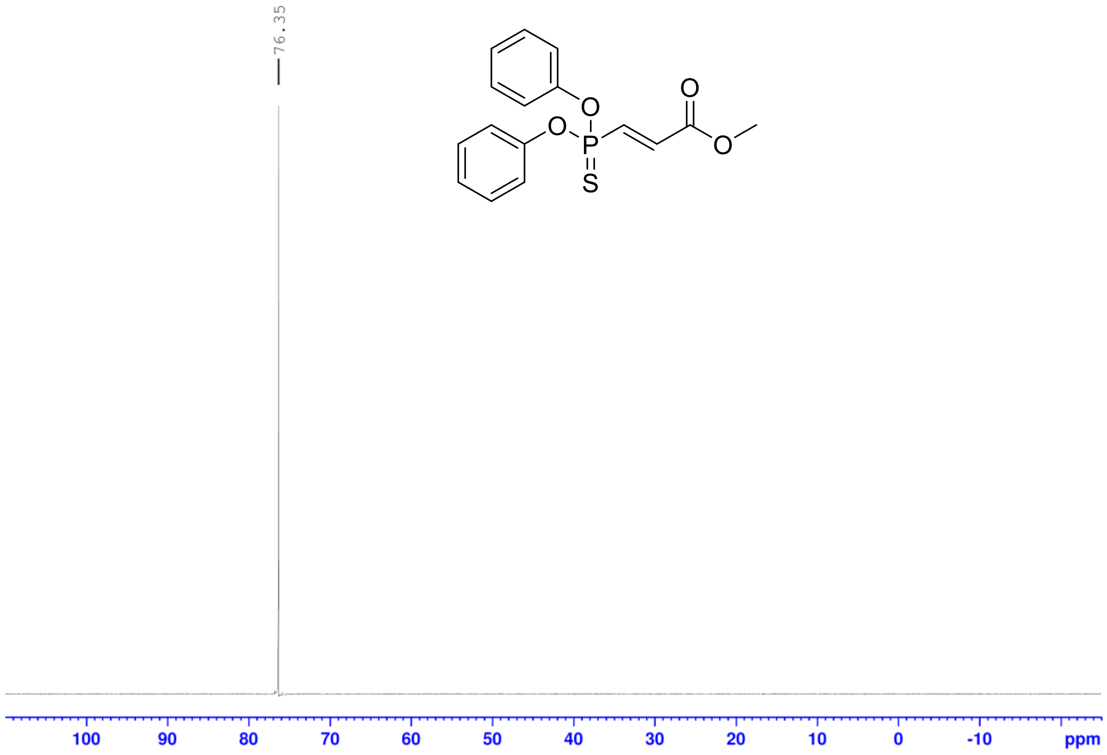

$^{31}P\{^{1}H\}$ NMR (162 MHz, $CDCl_3$) of compound **6a**

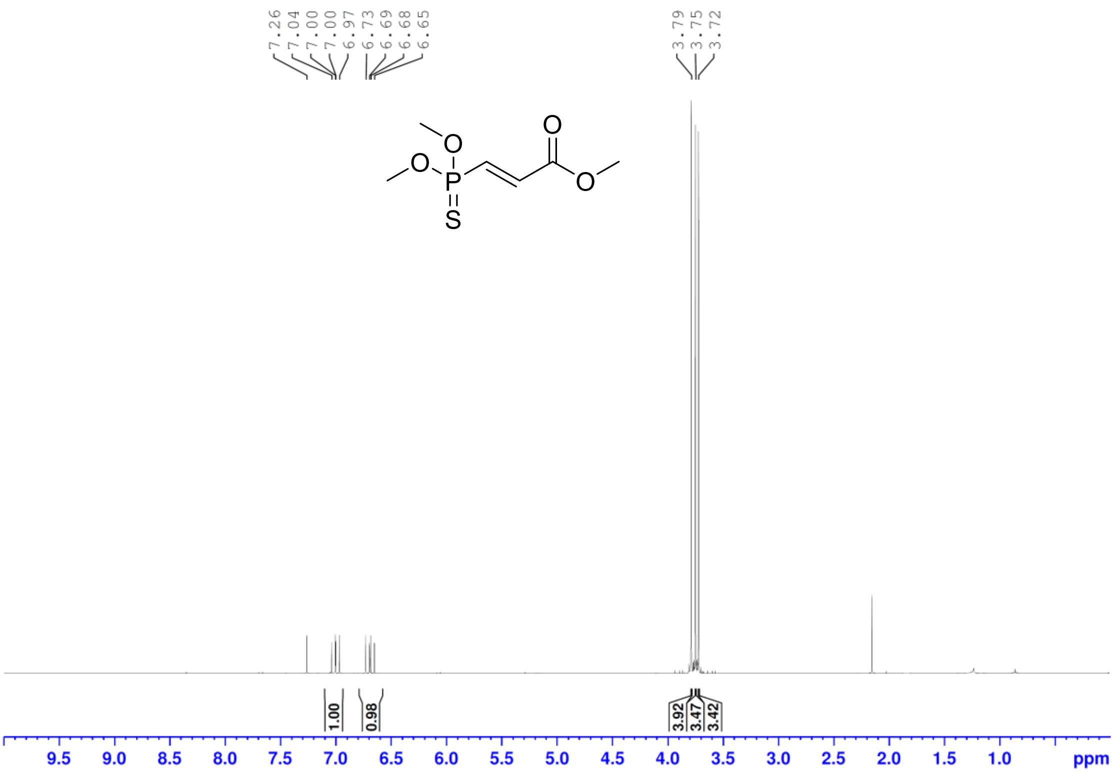

^{1}H NMR (500 MHz, $CDCl_3$) of compound **6b**

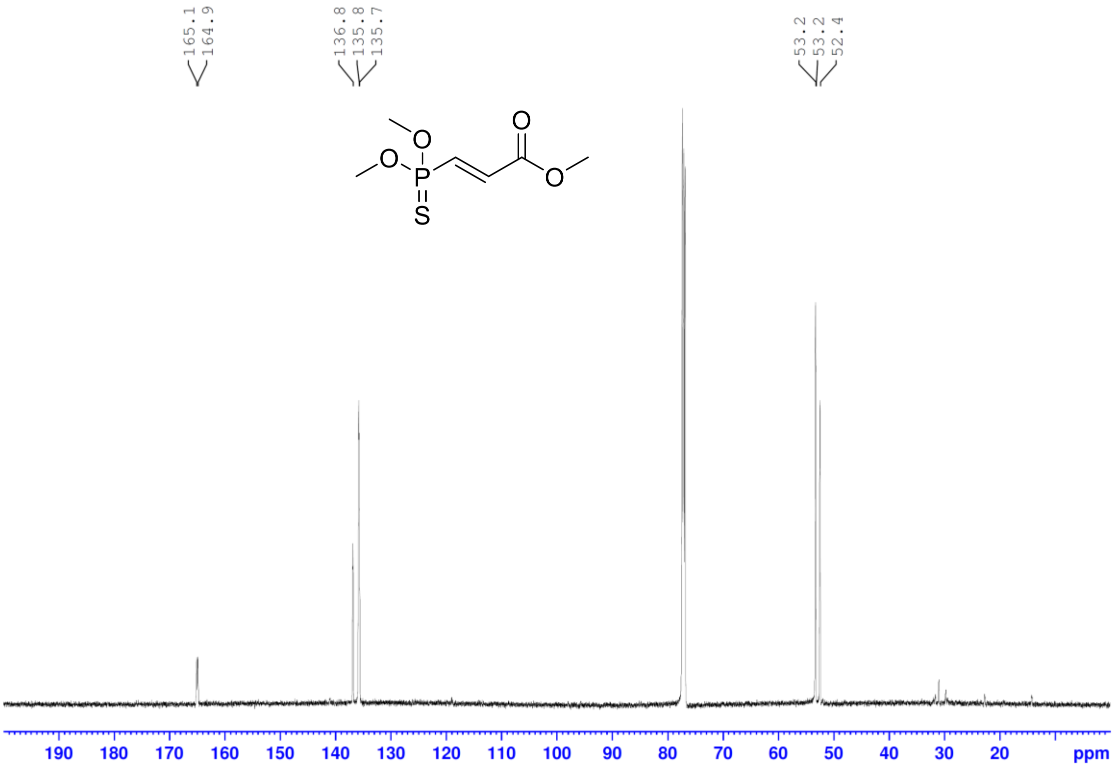

$^{13}C\{^{1}H\}$ NMR (125 MHz, $CDCl_3$) of compound **6b**

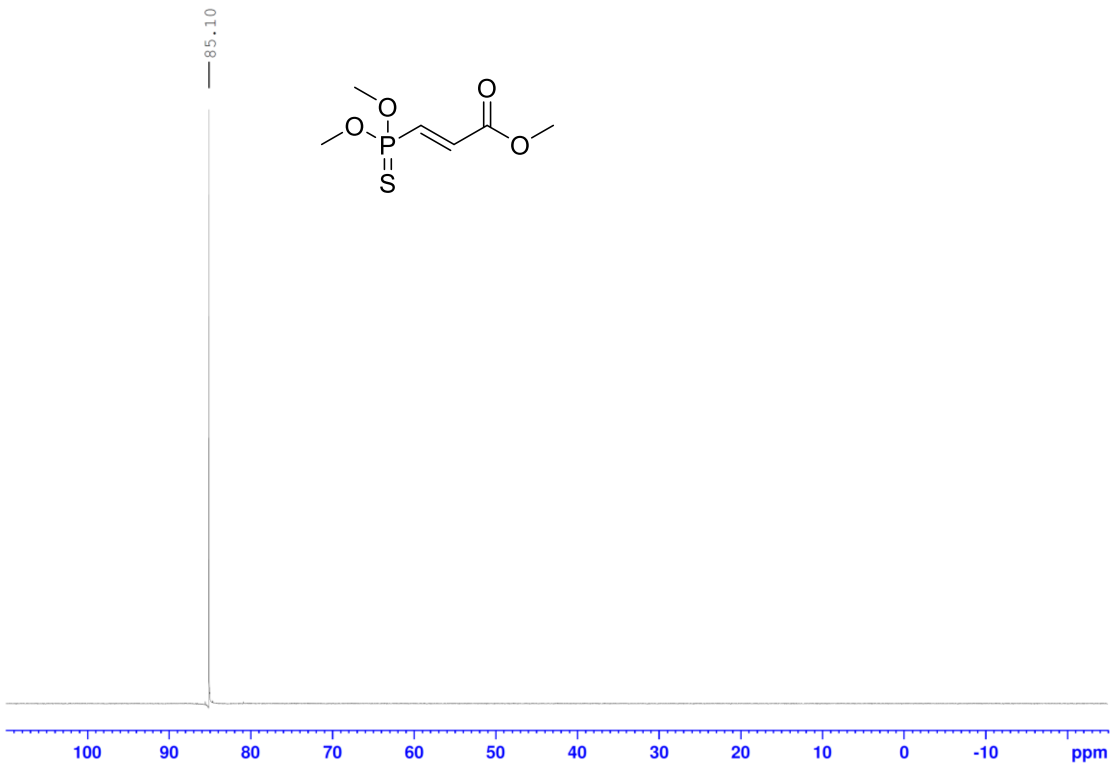

$^{31}P\{^1H\}$ NMR (162 MHz, $CDCl_3$) of compound **6b**

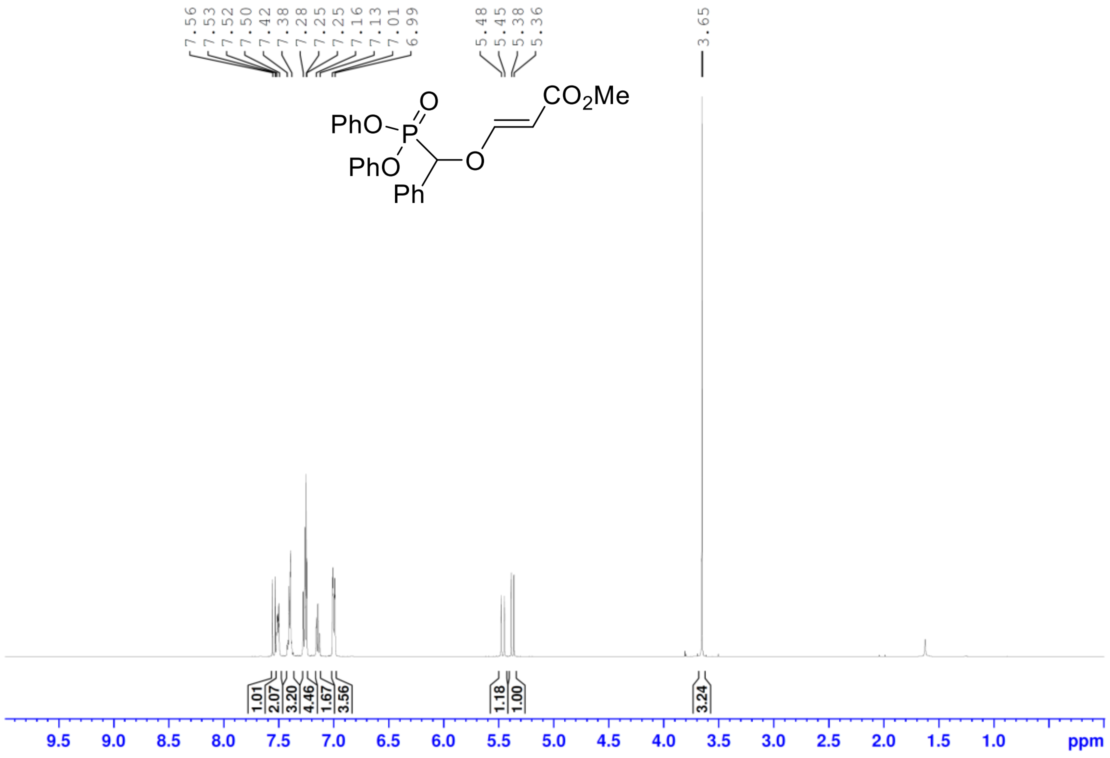

^1H NMR (500 MHz, $CDCl_3$) of compound **8aa**

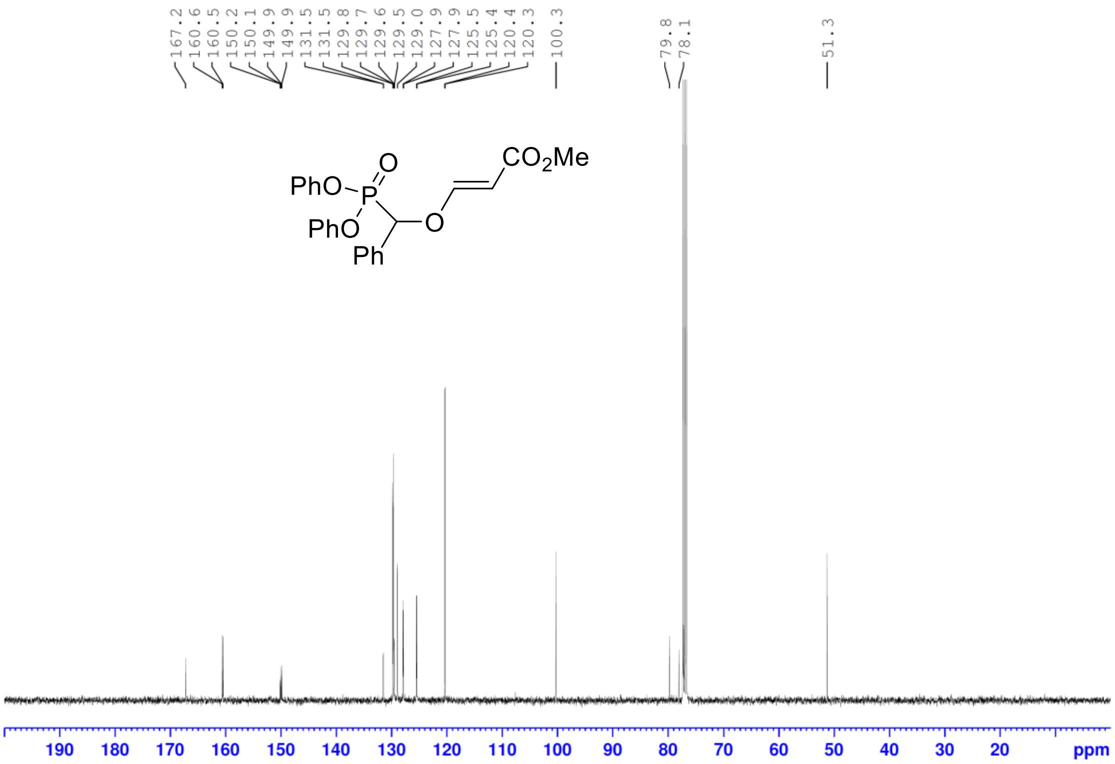

$^{13}C\{^{1}H\}$ NMR (100 MHz, $CDCl_3$) of compound **8aa**

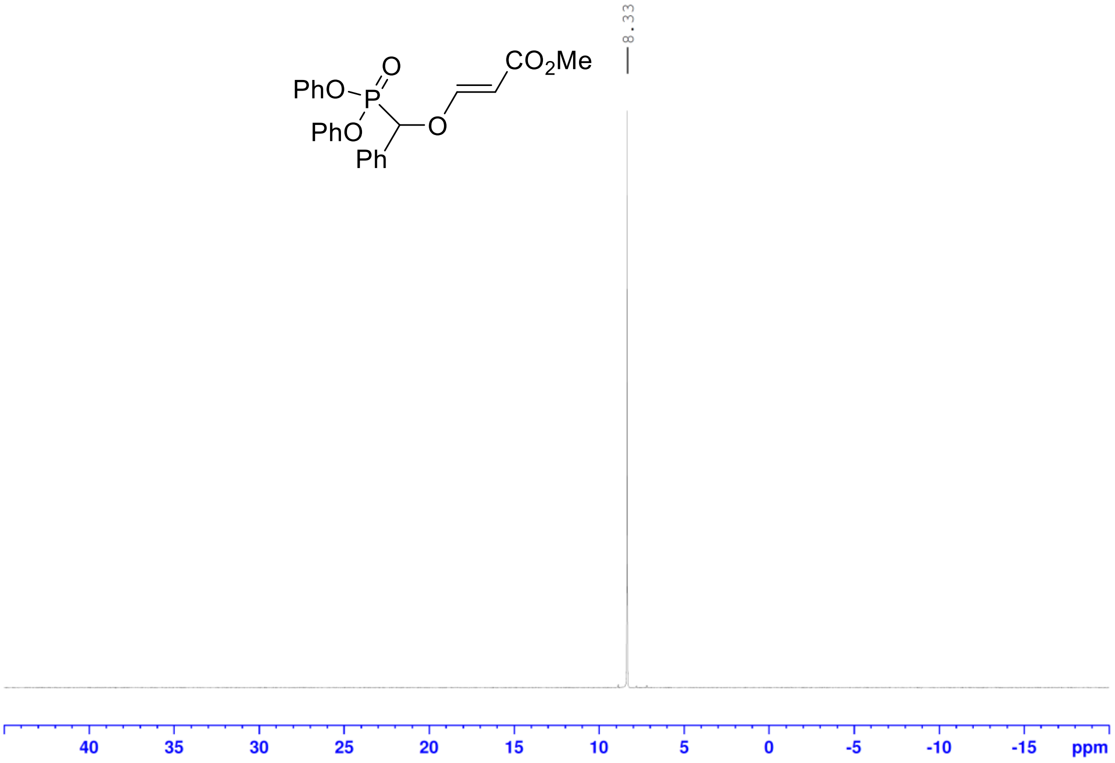

$^{31}P\{^{1}H\}$ NMR (162 MHz, $CDCl_3$) of compound **8aa**

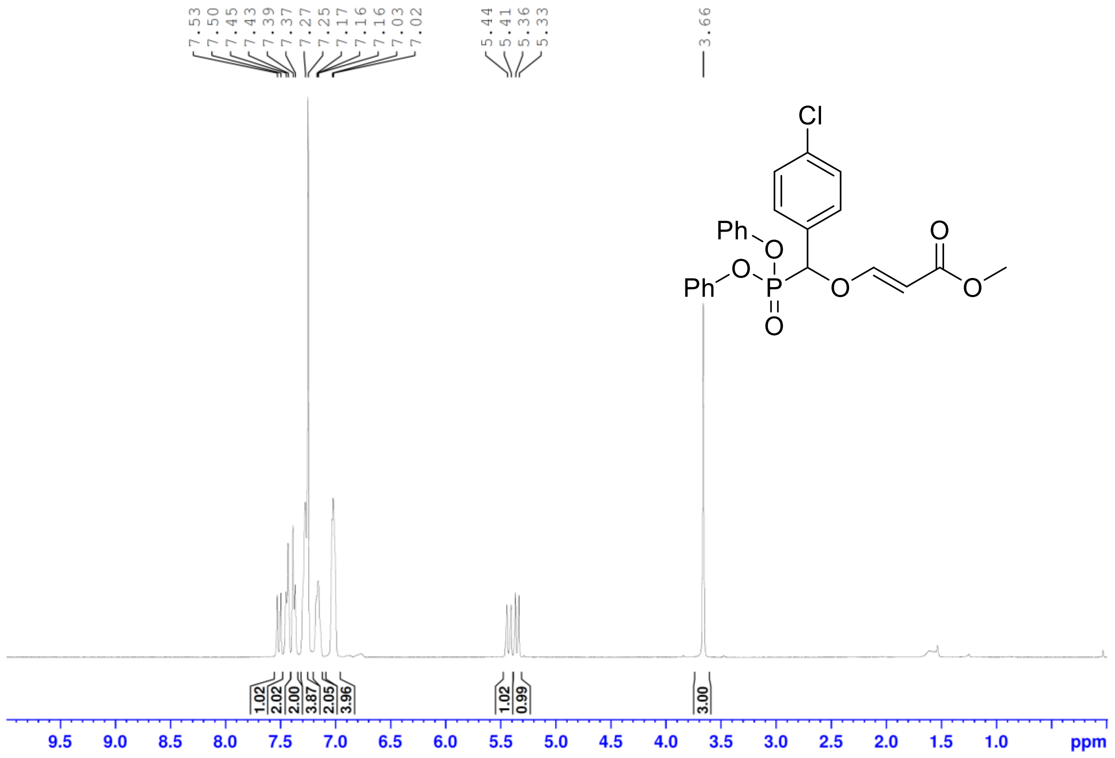

^{1}H NMR (400 MHz, $CDCl_3$) of compound **8ab**

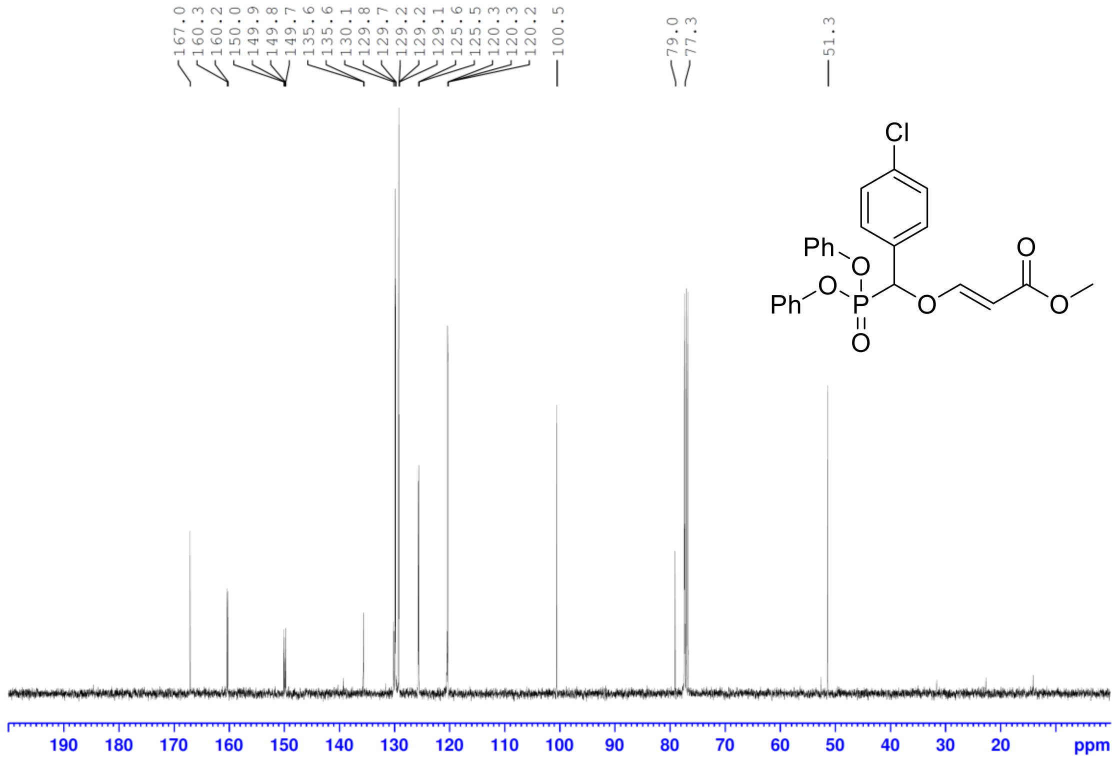

^{13}C{^{1}H} NMR (100 MHz, $CDCl_3$) of compound **8ab**

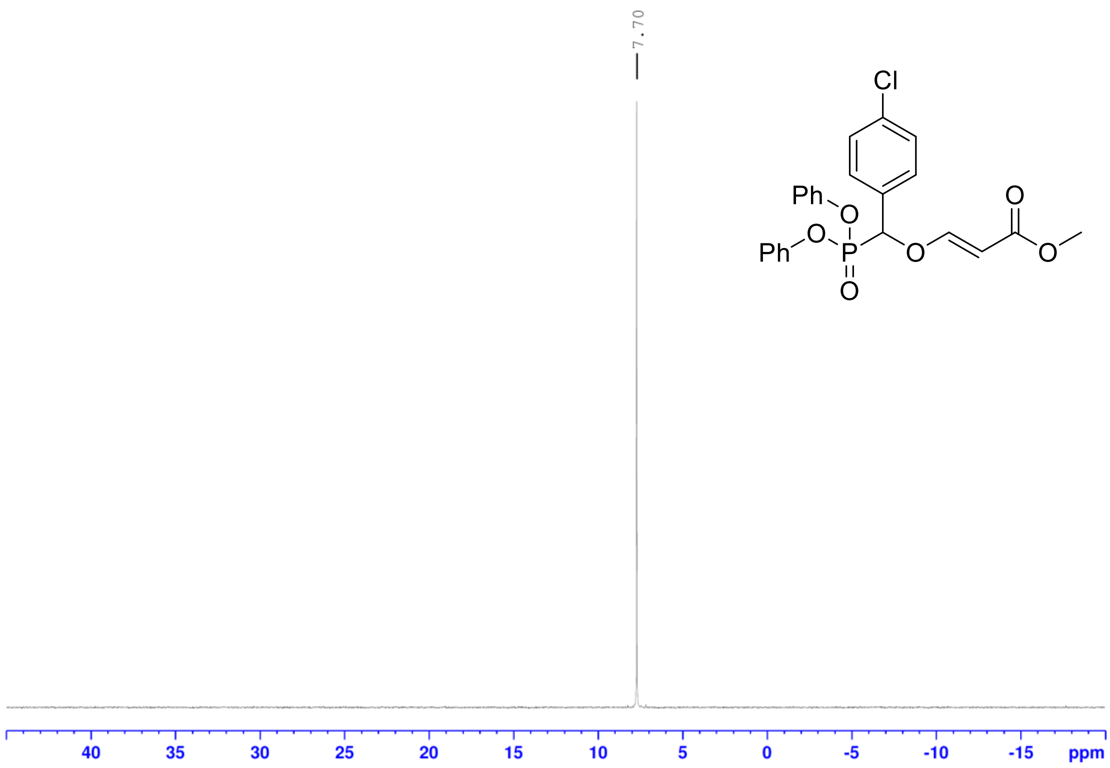

$^{31}P\{^1H\}$ NMR (162 MHz, $CDCl_3$) of compound **8ab**

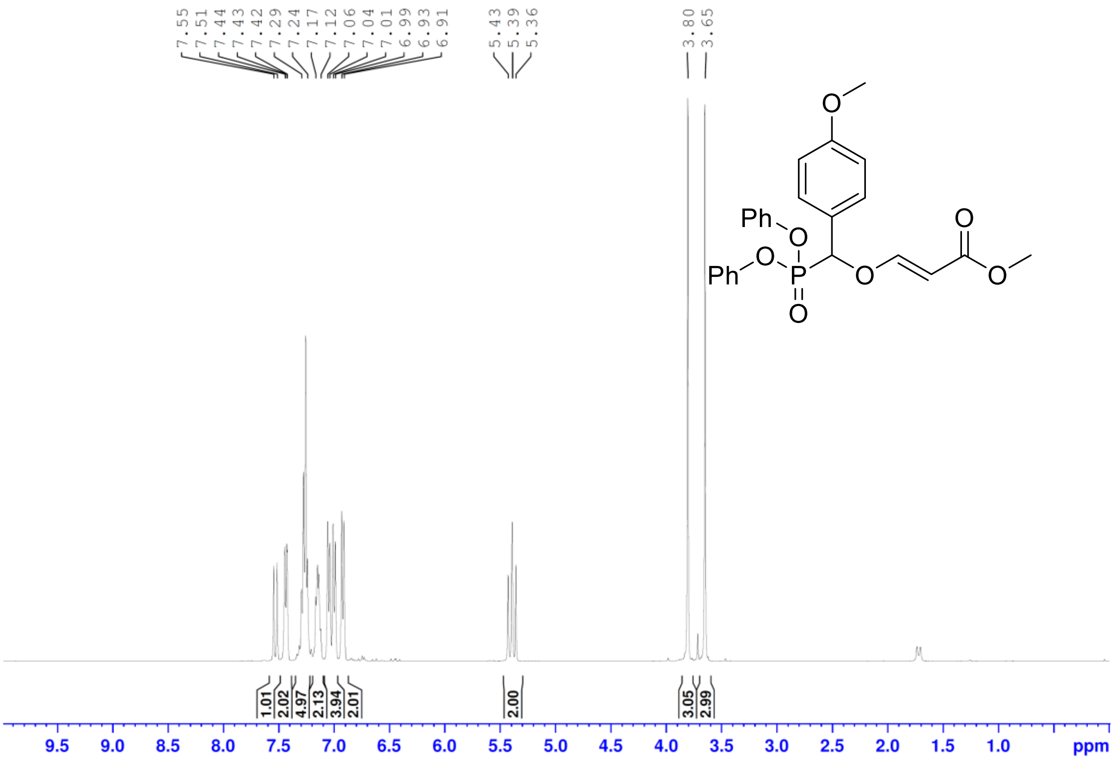

^1H NMR (400 MHz, $CDCl_3$) of compound **8ac**

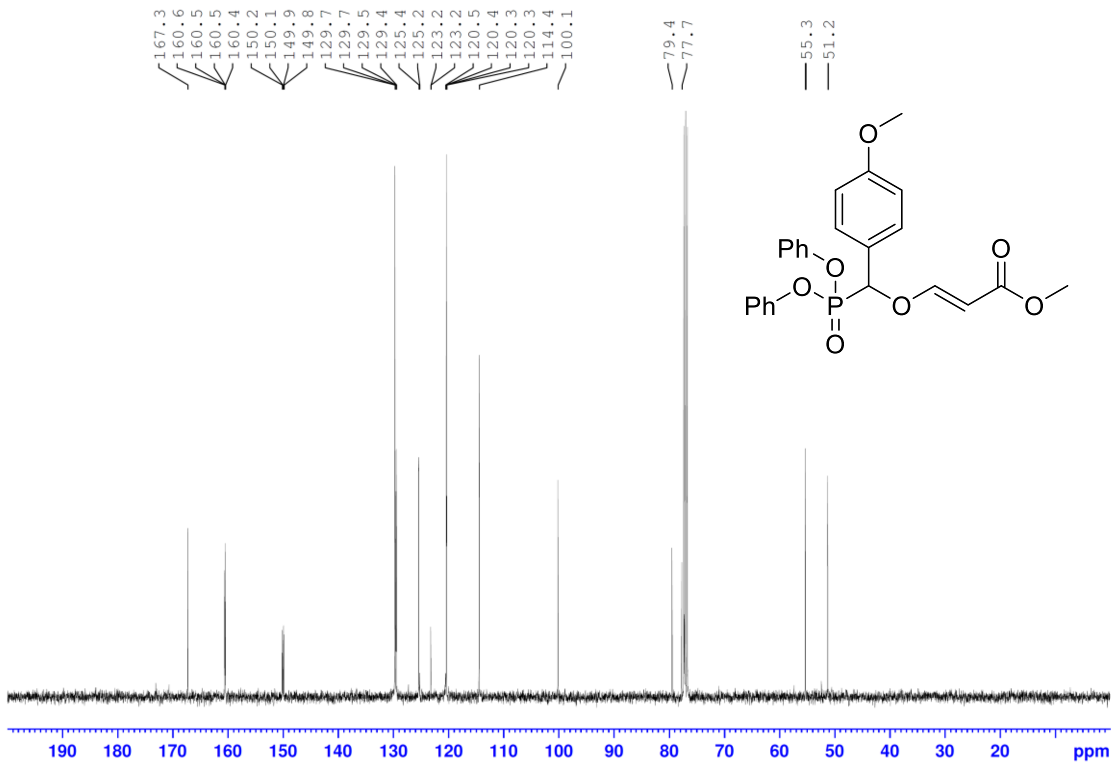

$^{13}C\{^1H\}$ NMR (100 MHz, $CDCl_3$) of compound **8ac**

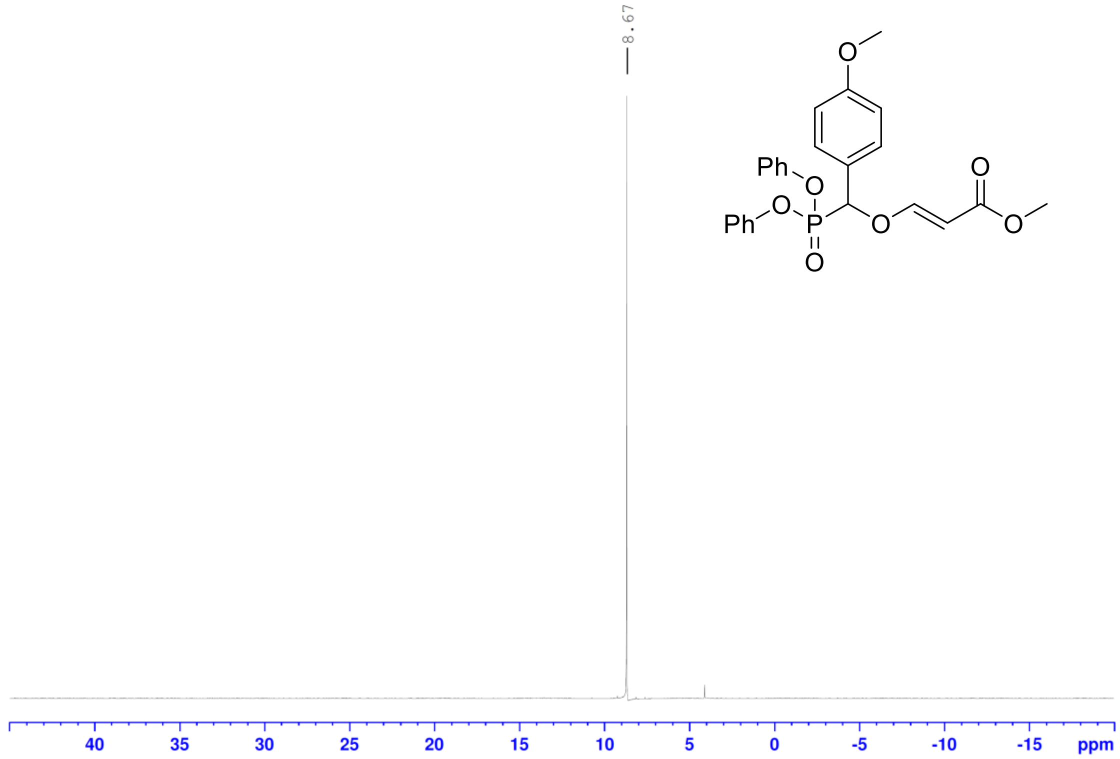

$^{31}P\{^1H\}$ NMR (162 MHz, $CDCl_3$) of compound **8ac**

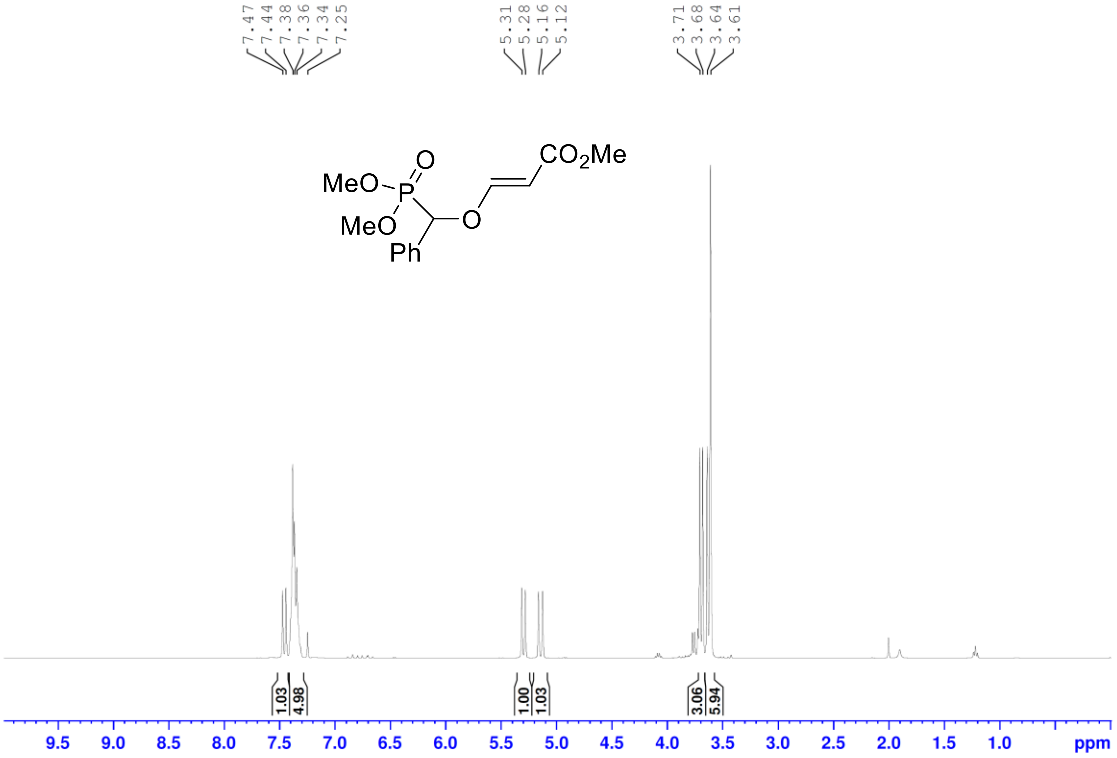

^{1}H NMR (400 MHz, $CDCl_3$) of compound **8ba**

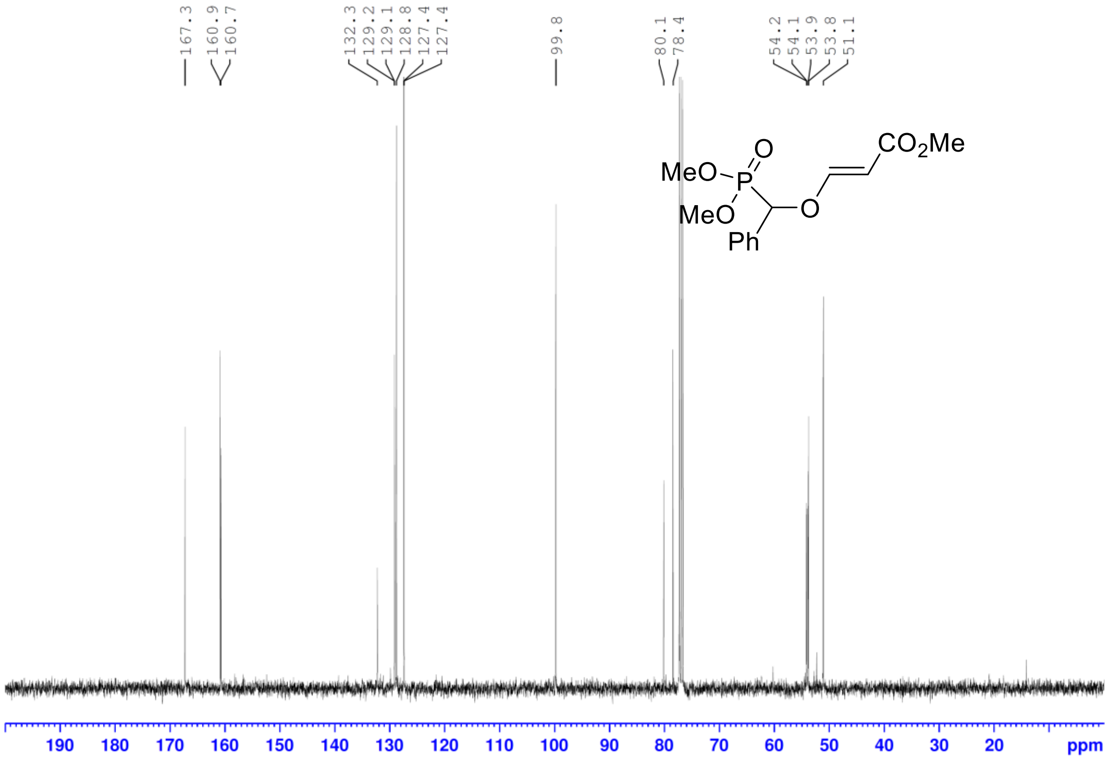

$^{13}C\{^{1}H\}$ NMR (100 MHz, $CDCl_3$) of compound **8ba**

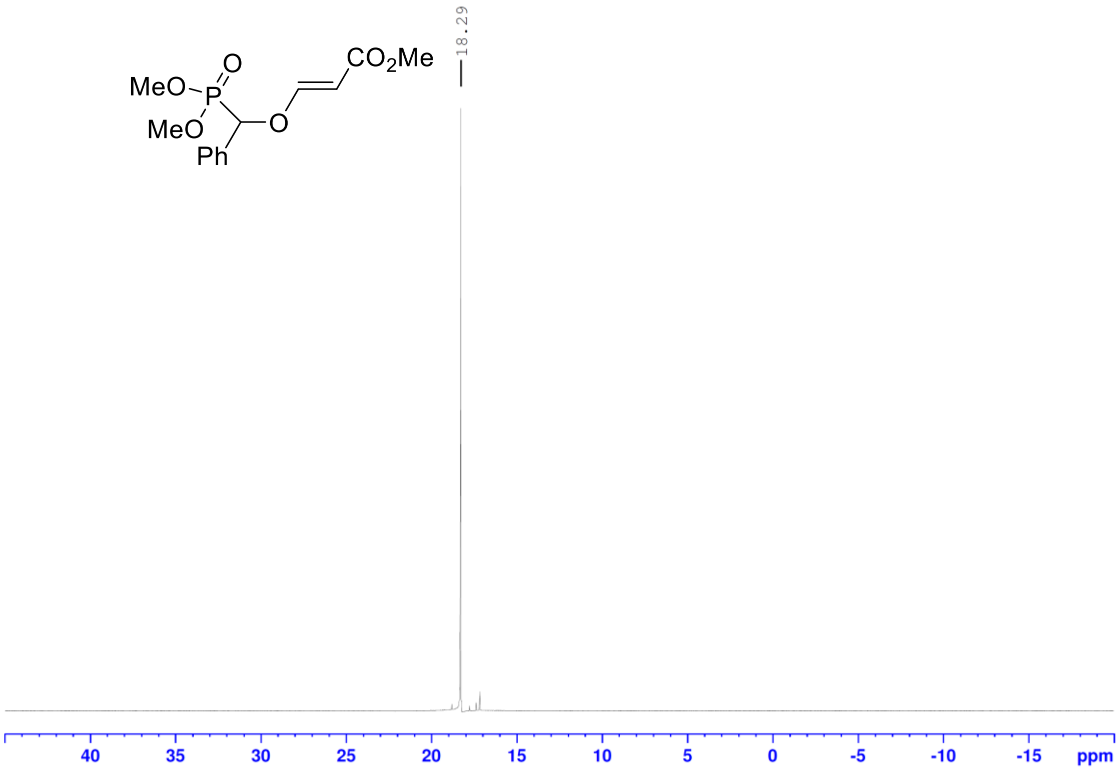

$^{31}P\{^{1}H\}$ NMR (162 MHz, $CDCl_3$) of compound **8ba**

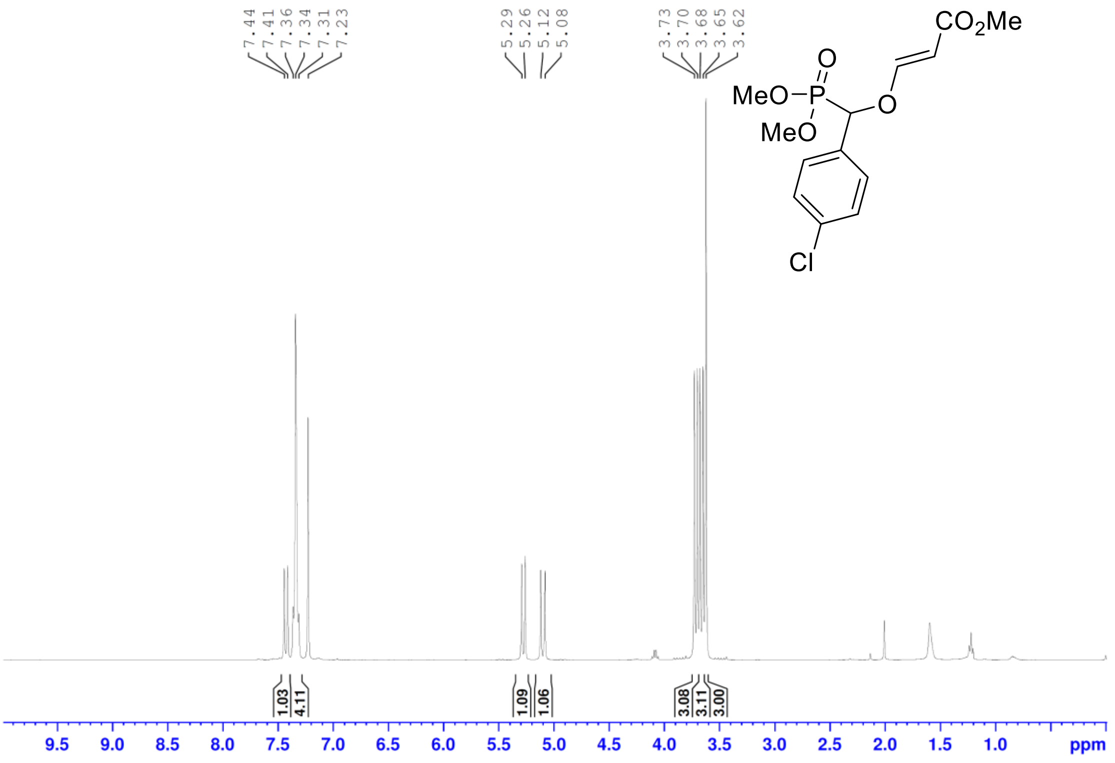

^{1}H NMR (400 MHz, $CDCl_3$) of compound **8bb**

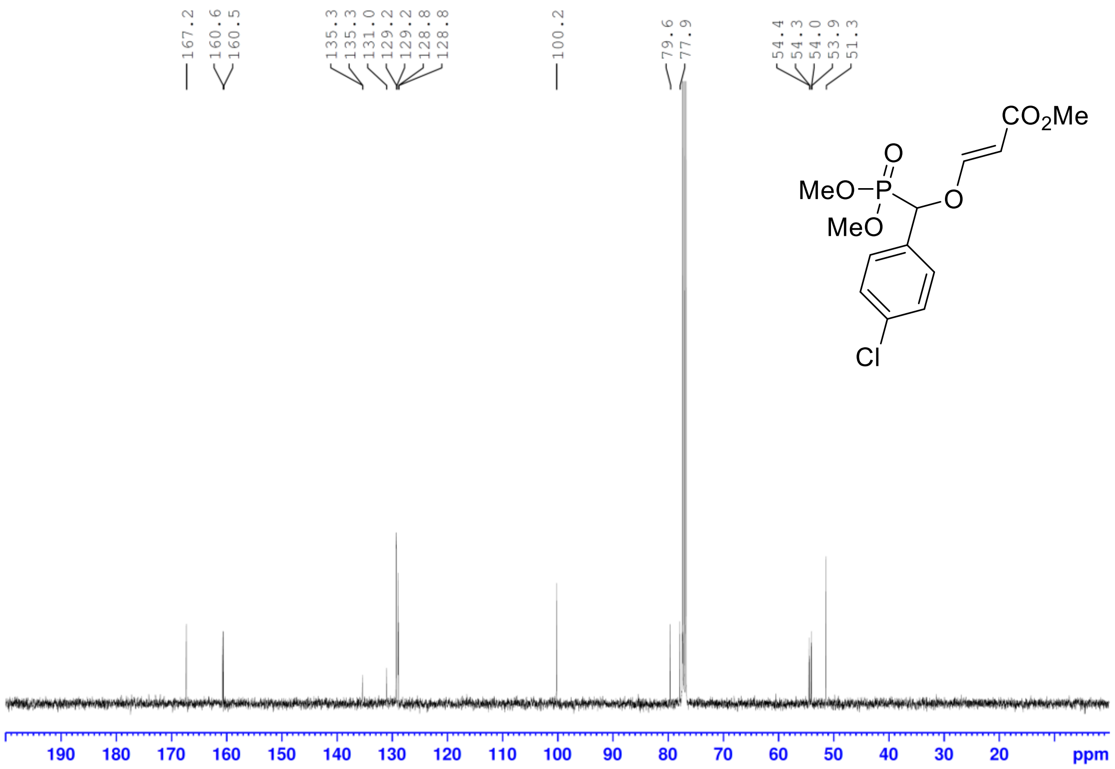

$^{13}C\{^1H\}$ NMR (100 MHz, $CDCl_3$) of compound **8bb**

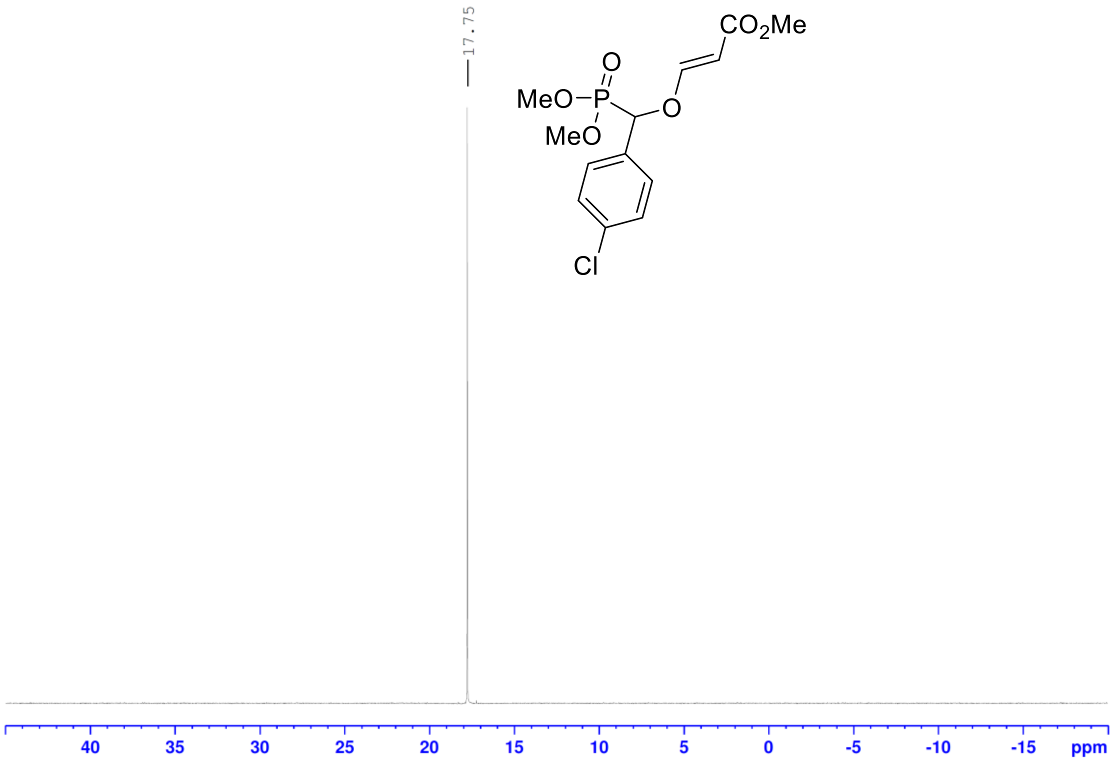

$^{31}P\{^1H\}$ NMR (162 MHz, $CDCl_3$) of compound **8bb**

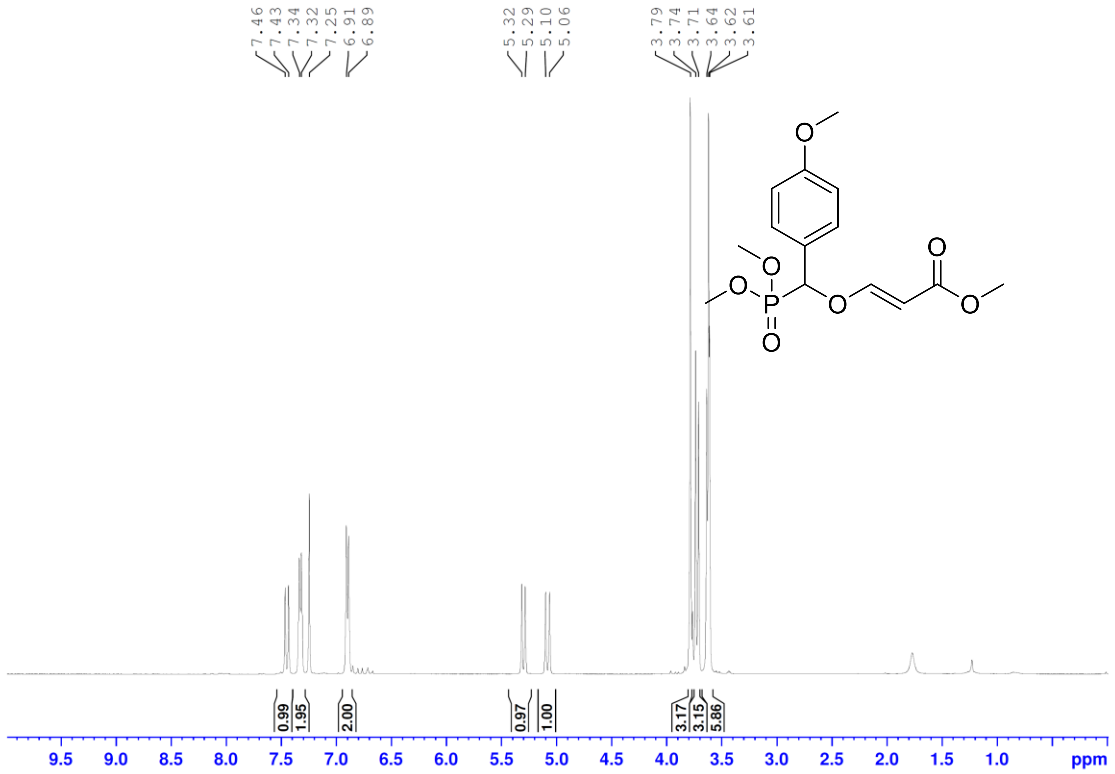

^{1}H NMR (400 MHz, $CDCl_3$) of compound **8bc**

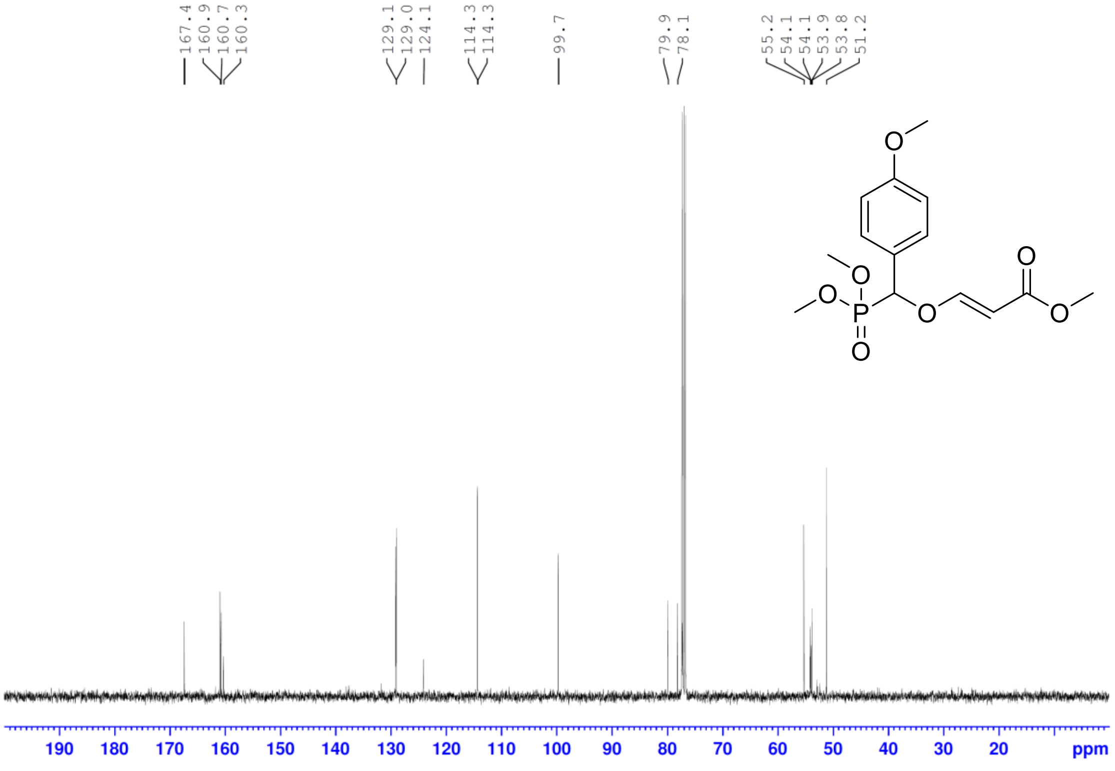

$^{13}C\{^1H\}$ NMR (100 MHz, $CDCl_3$) of compound **8bc**

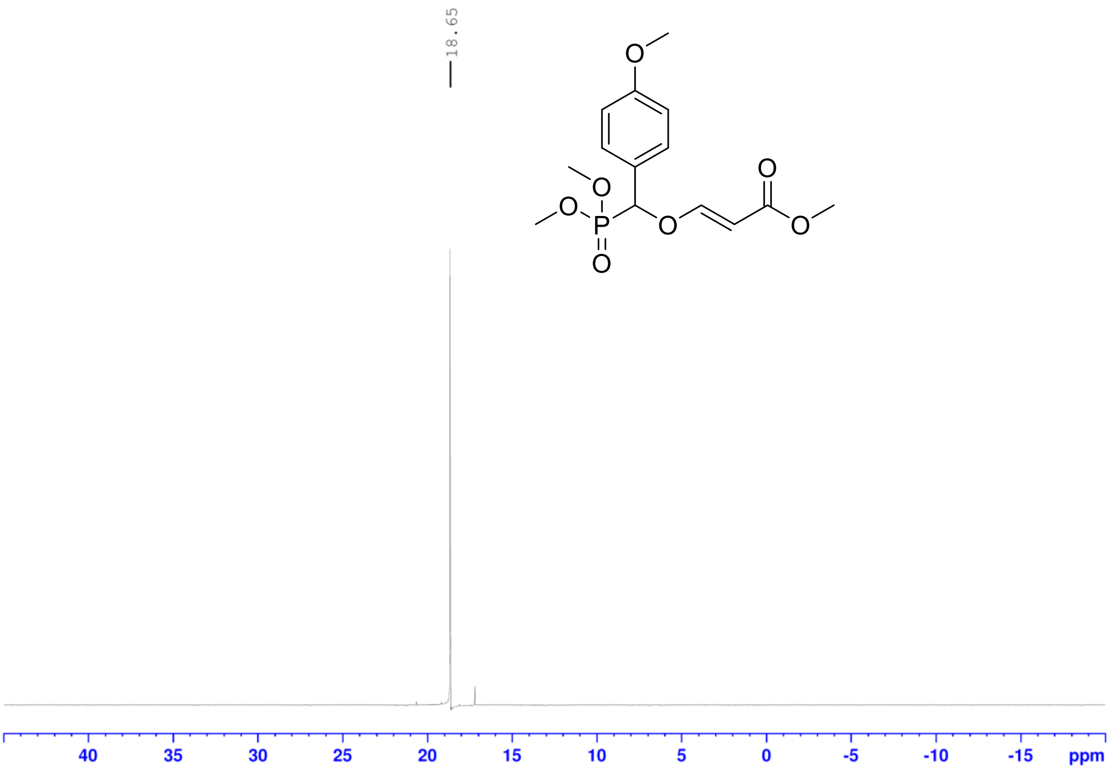

$^{31}P\{^{1}H\}$ NMR (162 MHz, $CDCl_3$) of compound **8bc**

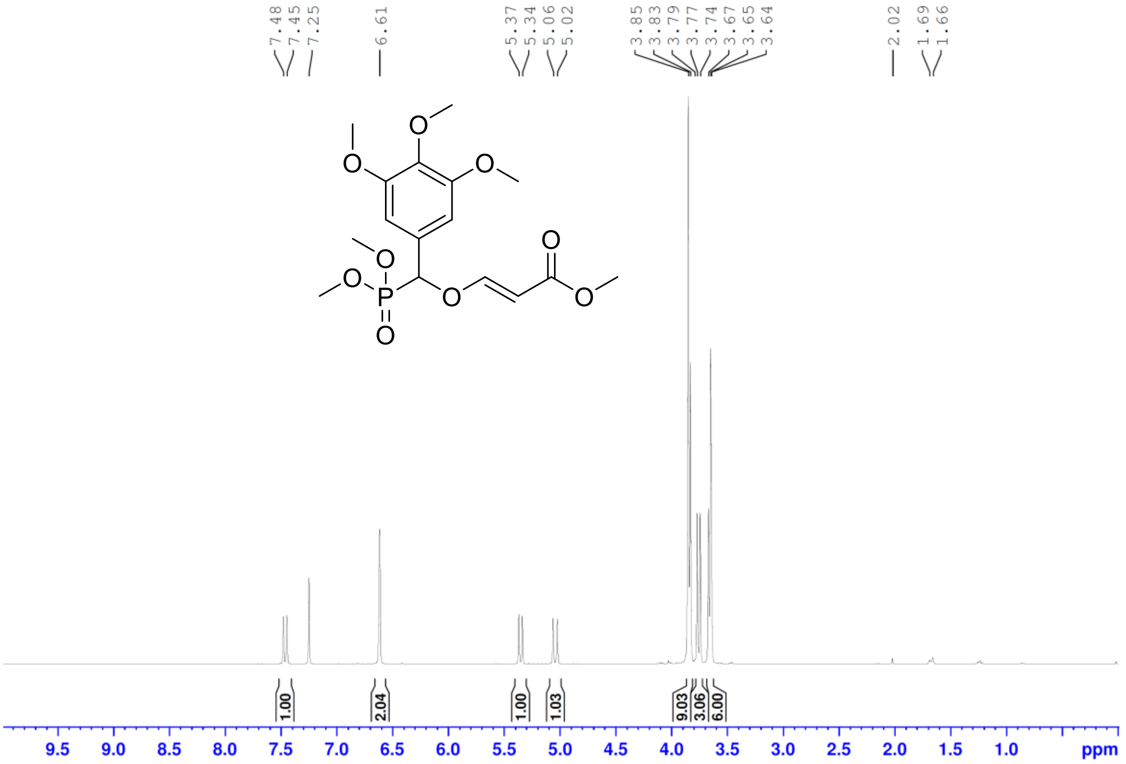

^{1}H NMR (400 MHz, $CDCl_3$) of compound **8bd**

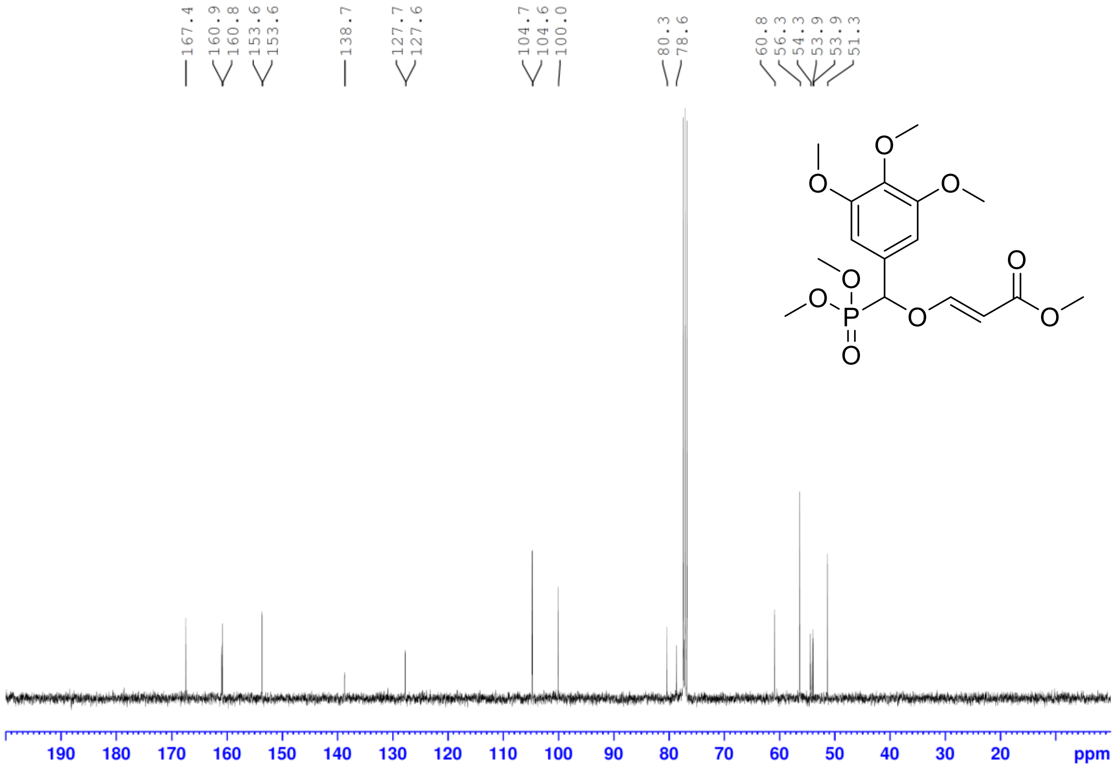

$^{13}C\{^1H\}$ NMR (100 MHz, $CDCl_3$) of compound **8bd**

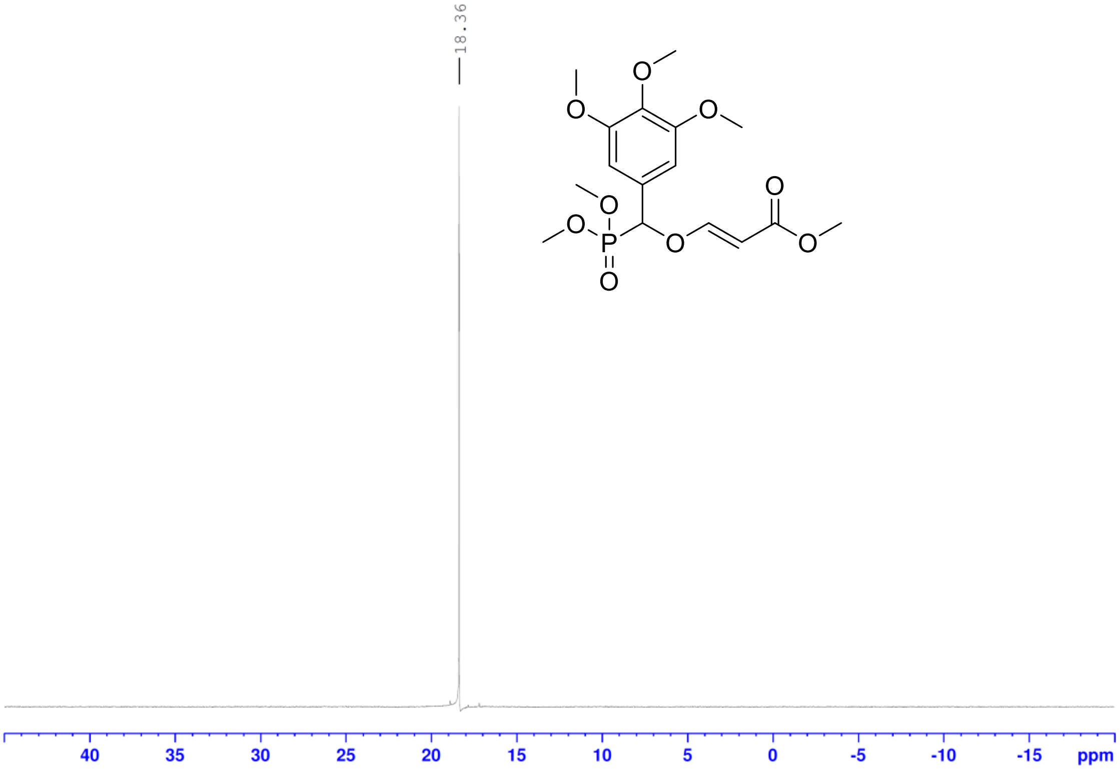

$^{31}P\{^1H\}$ NMR (162 MHz, $CDCl_3$) of compound **8bd**

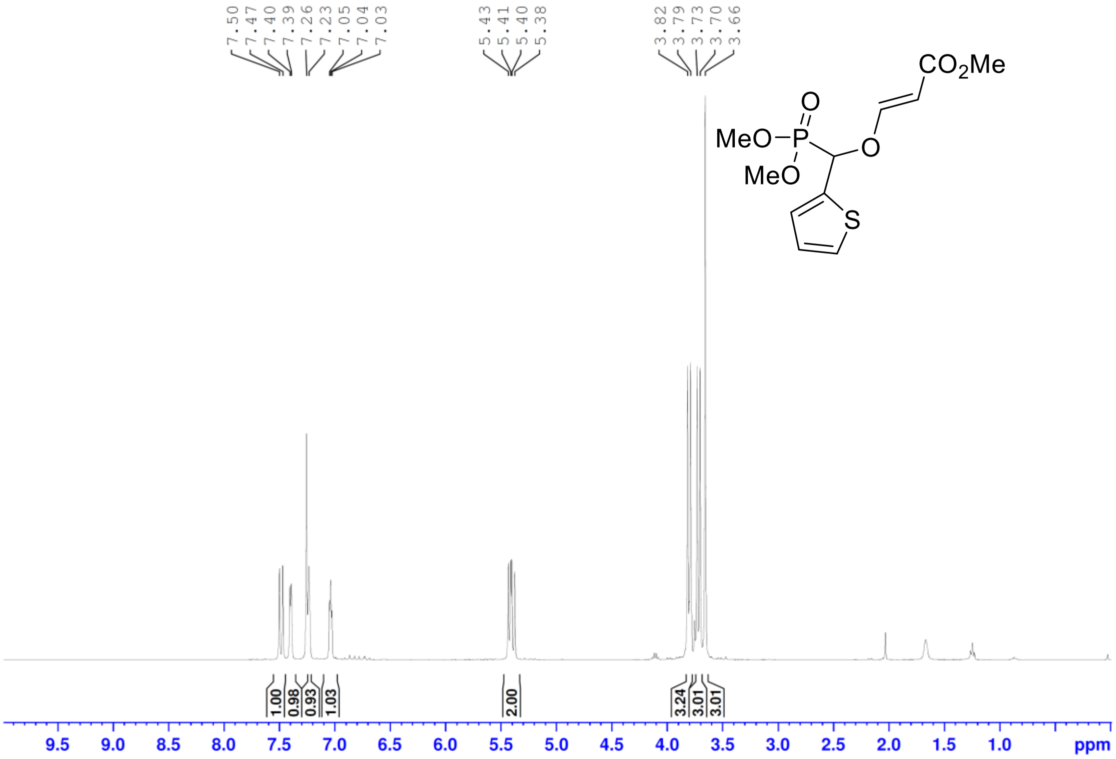

^{1}H NMR (400 MHz, $CDCl_3$) of compound **8be**

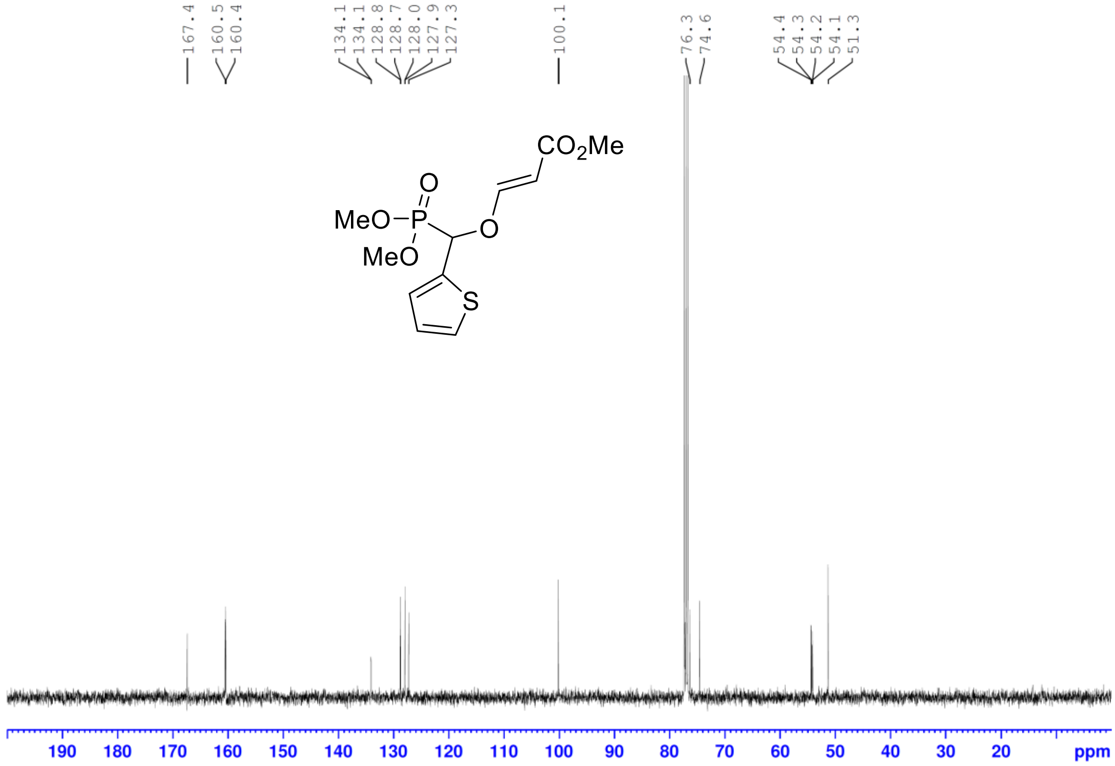

$^{13}C\{^{1}H\}$ NMR (100 MHz, $CDCl_3$) of compound **8be**

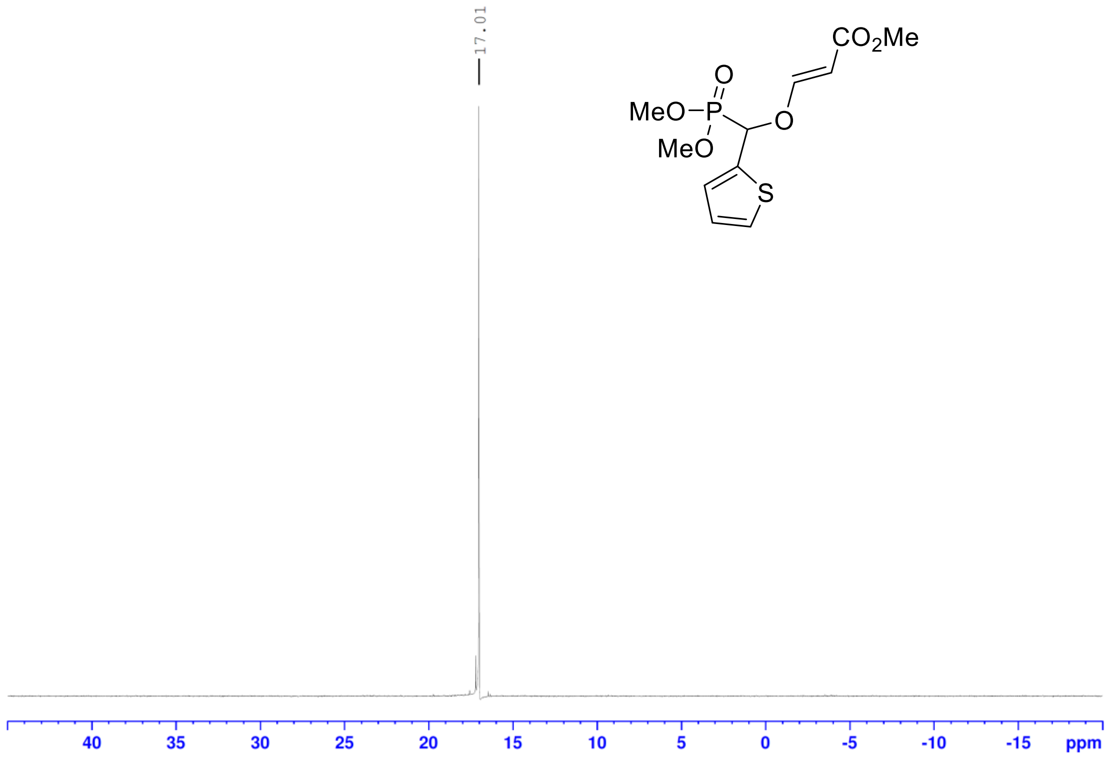

$^{31}P\{^1H\}$ NMR (162 MHz, $CDCl_3$) of compound **8be**

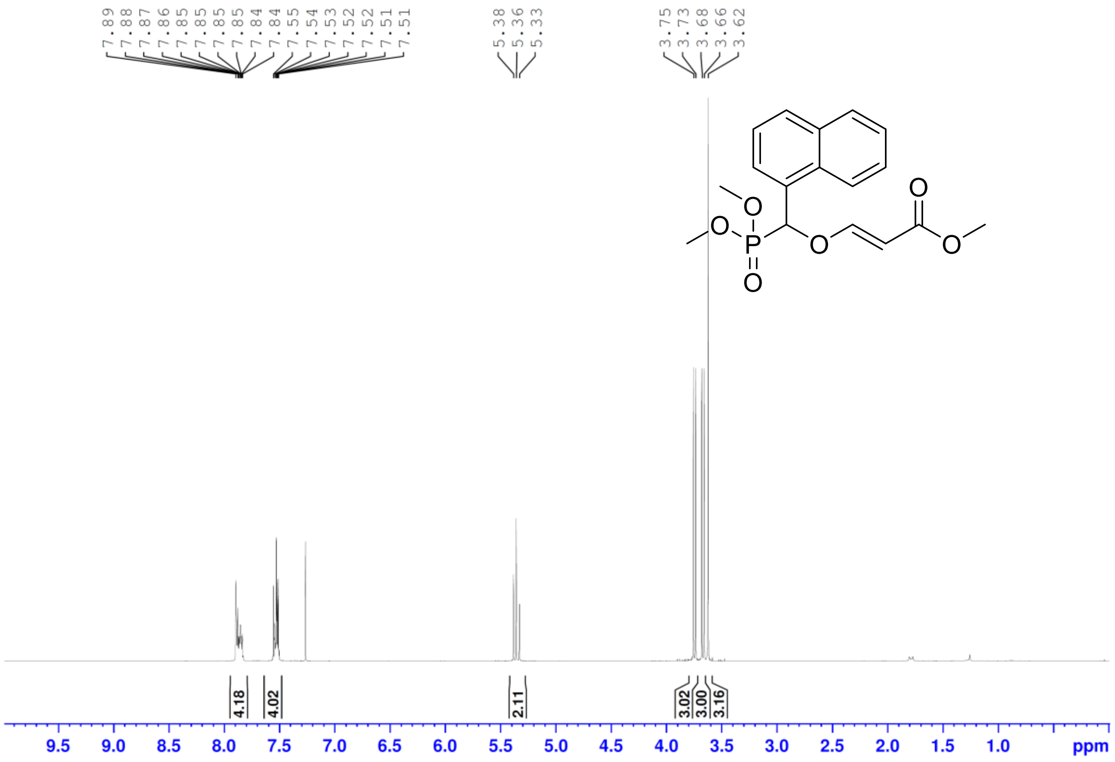

^1H NMR (4500 MHz, $CDCl_3$) of compound **8bf**

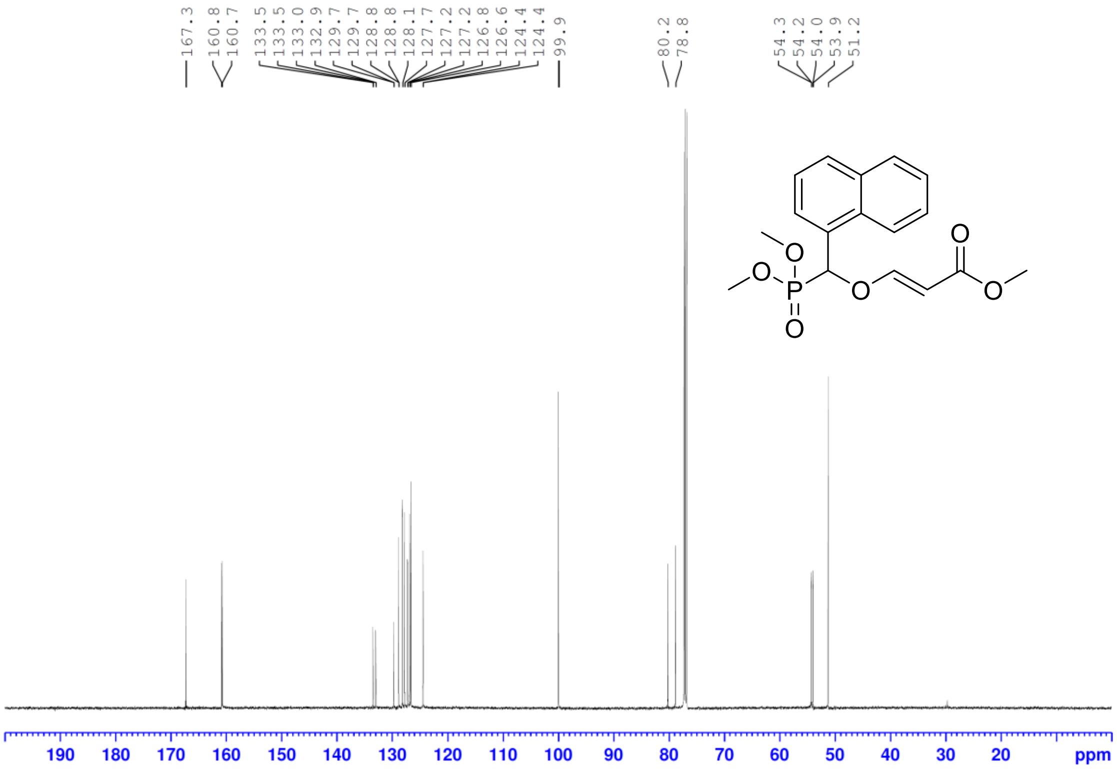

$^{13}C\{^{1}H\}$ NMR (125 MHz, $CDCl_3$) of compound **8bf**

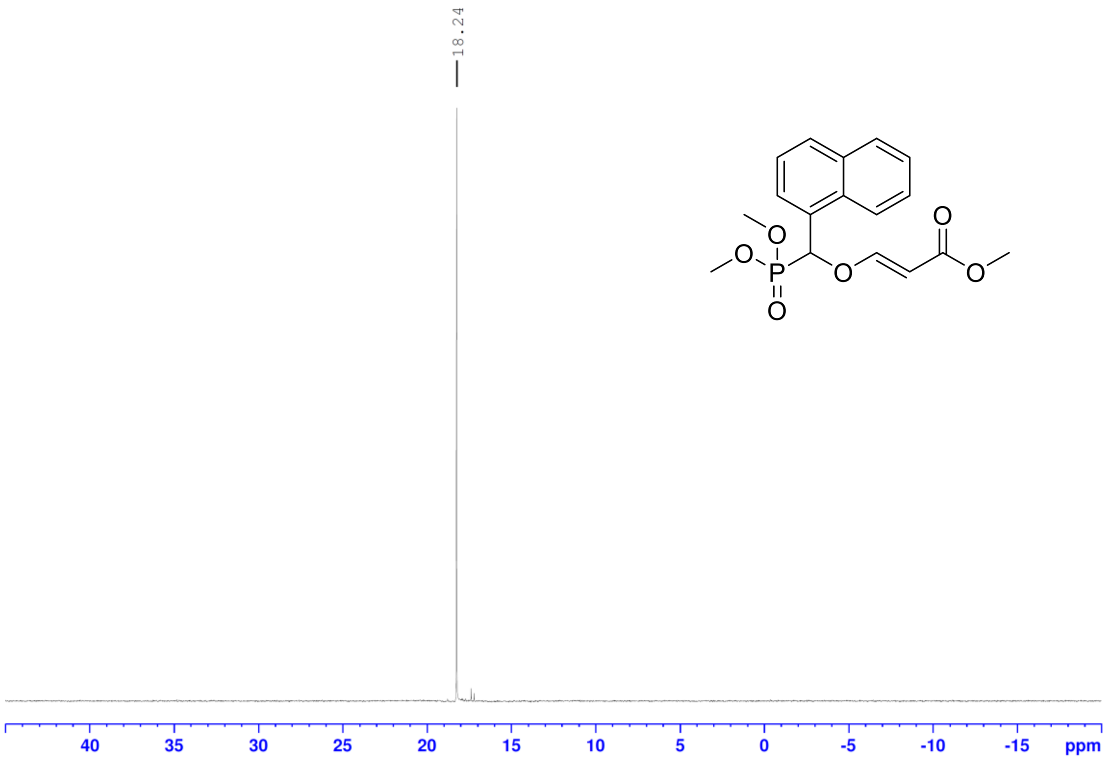

$^{31}P\{^{1}H\}$ NMR (162 MHz, $CDCl_3$) of compound **8bf**

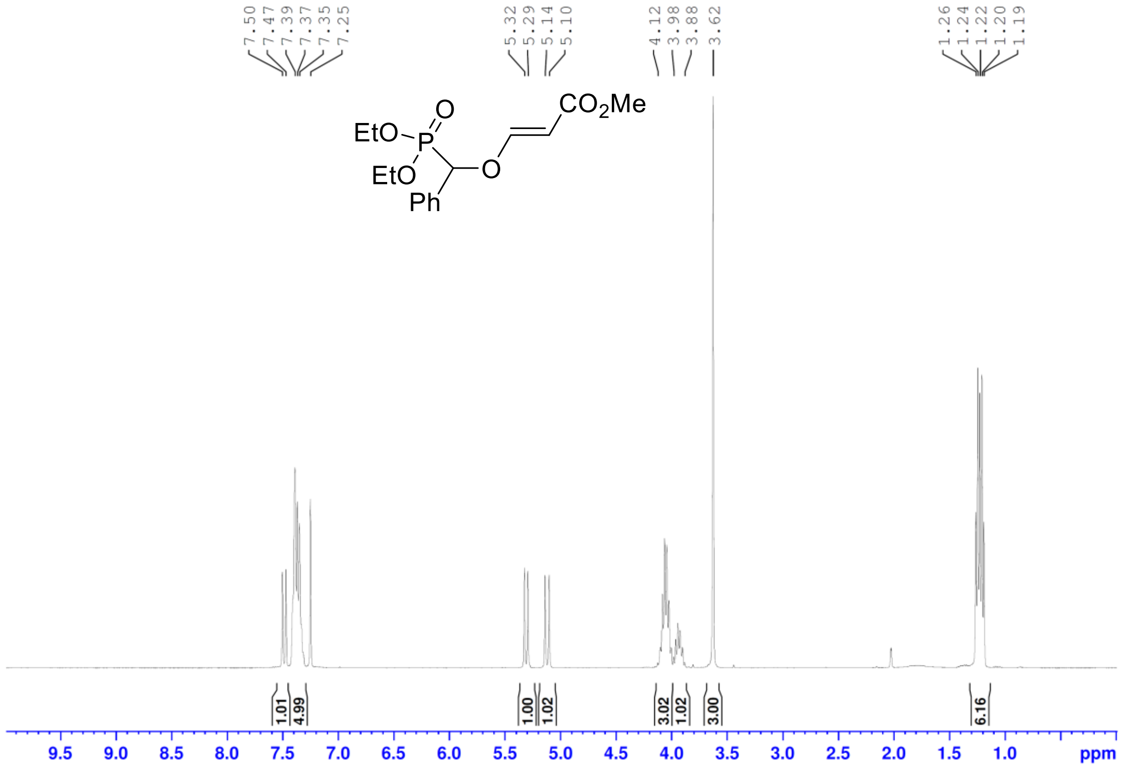

^{1}H NMR (400 MHz, $CDCl_3$) of compound **8ca**

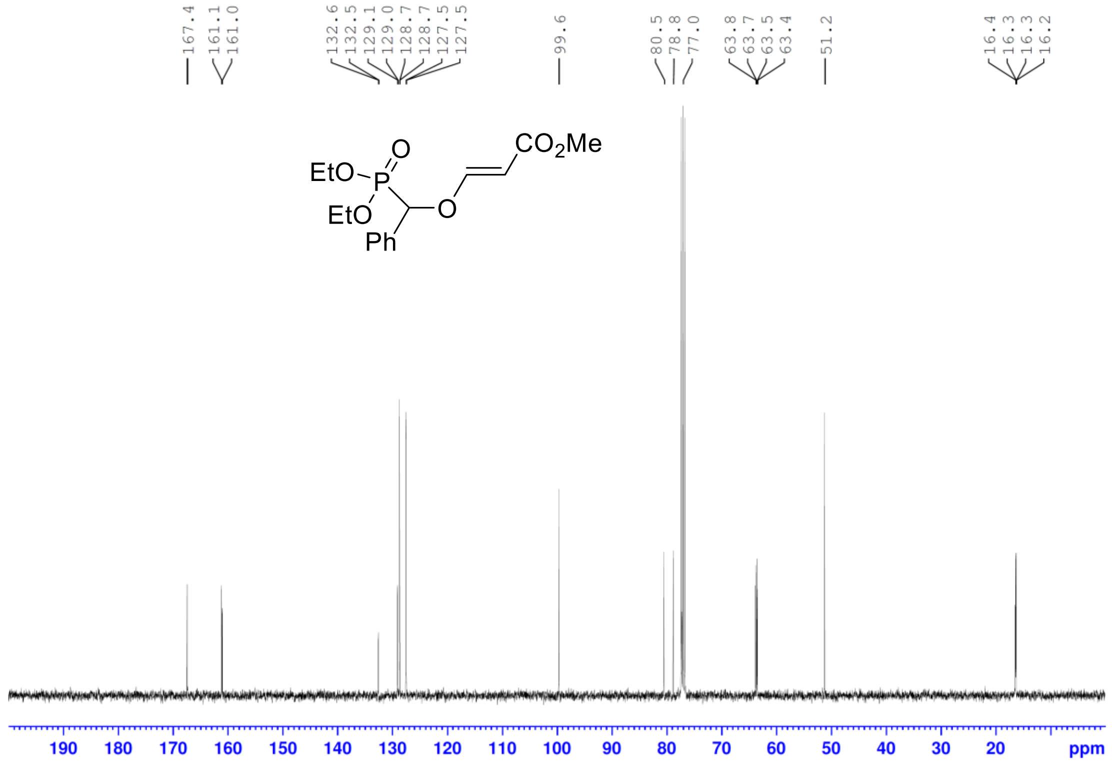

$^{13}C\{^{1}H\}$ NMR (100 MHz, $CDCl_3$) of compound **8ca**

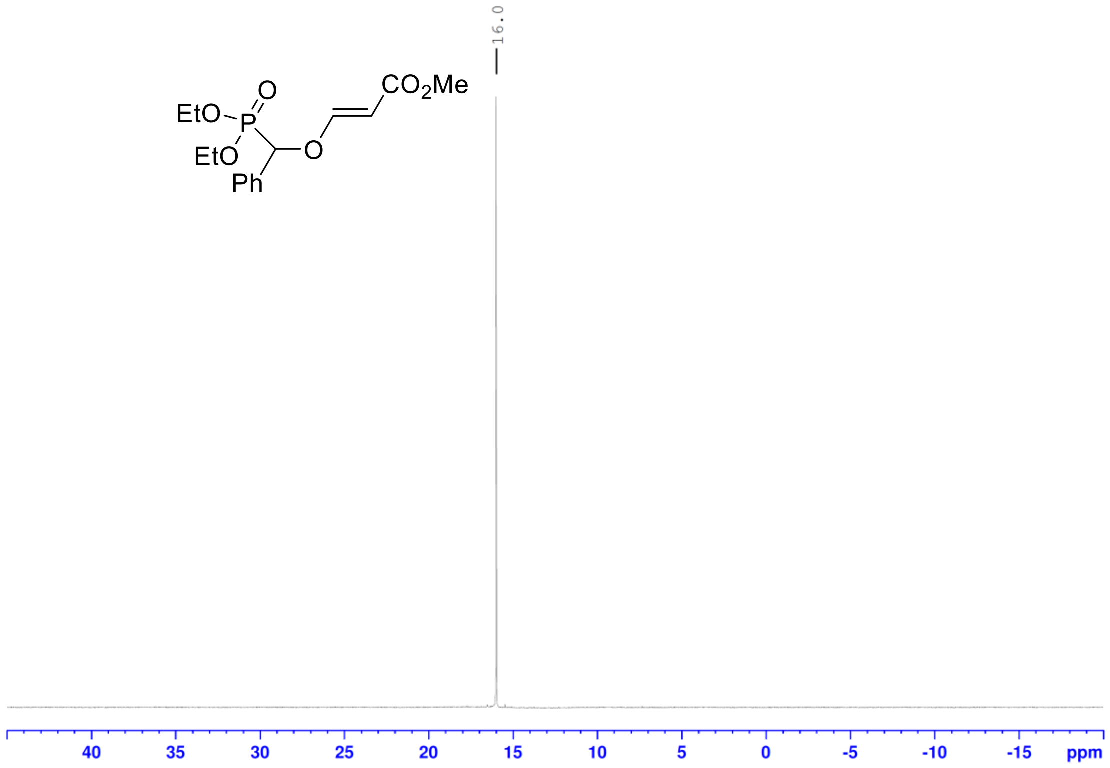

$^{31}P\{^{1}H\}$ NMR (162 MHz, $CDCl_3$) of compound **8ca**

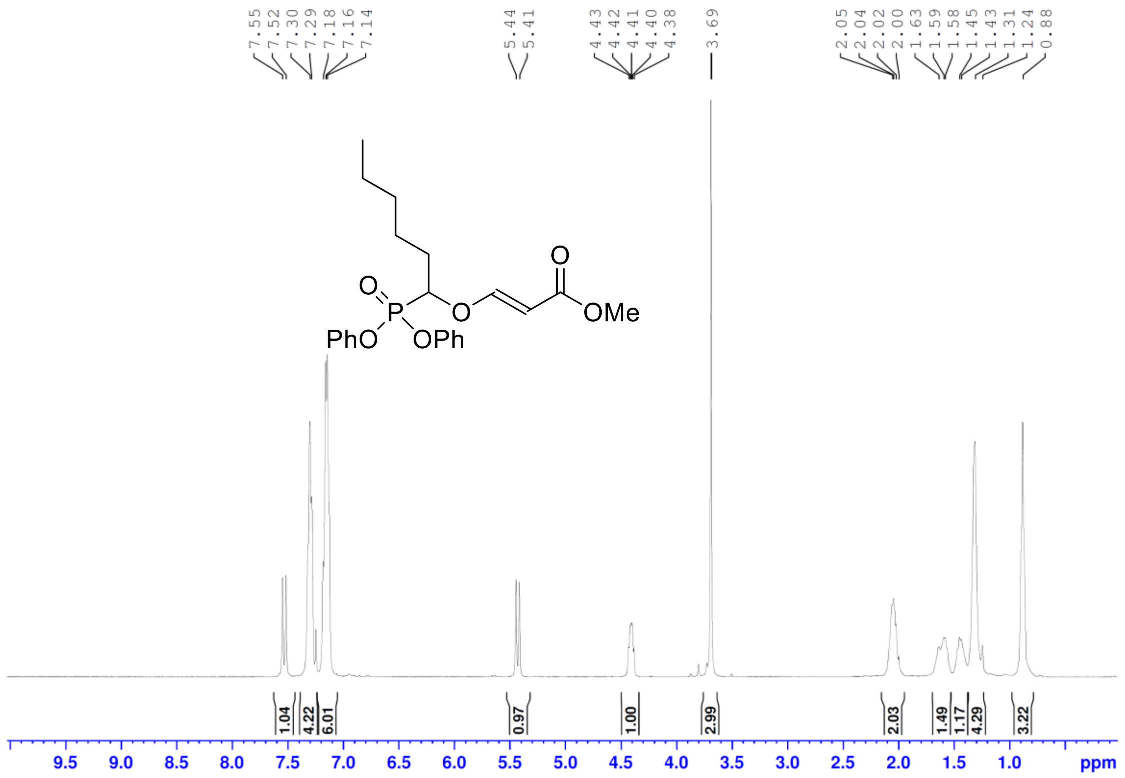

^{1}H NMR (400 MHz, $CDCl_3$) of compound **8ag**

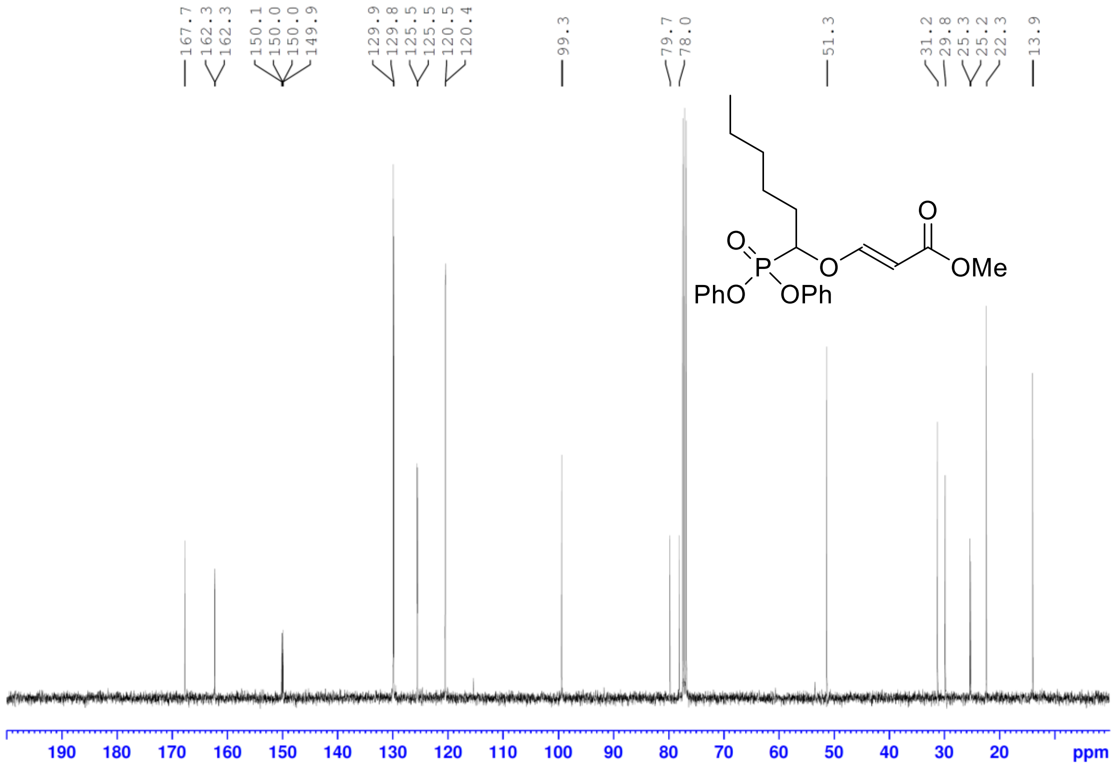

$^{13}C\{^{1}H\}$ NMR (100 MHz, $CDCl_3$) of compound **8ag**

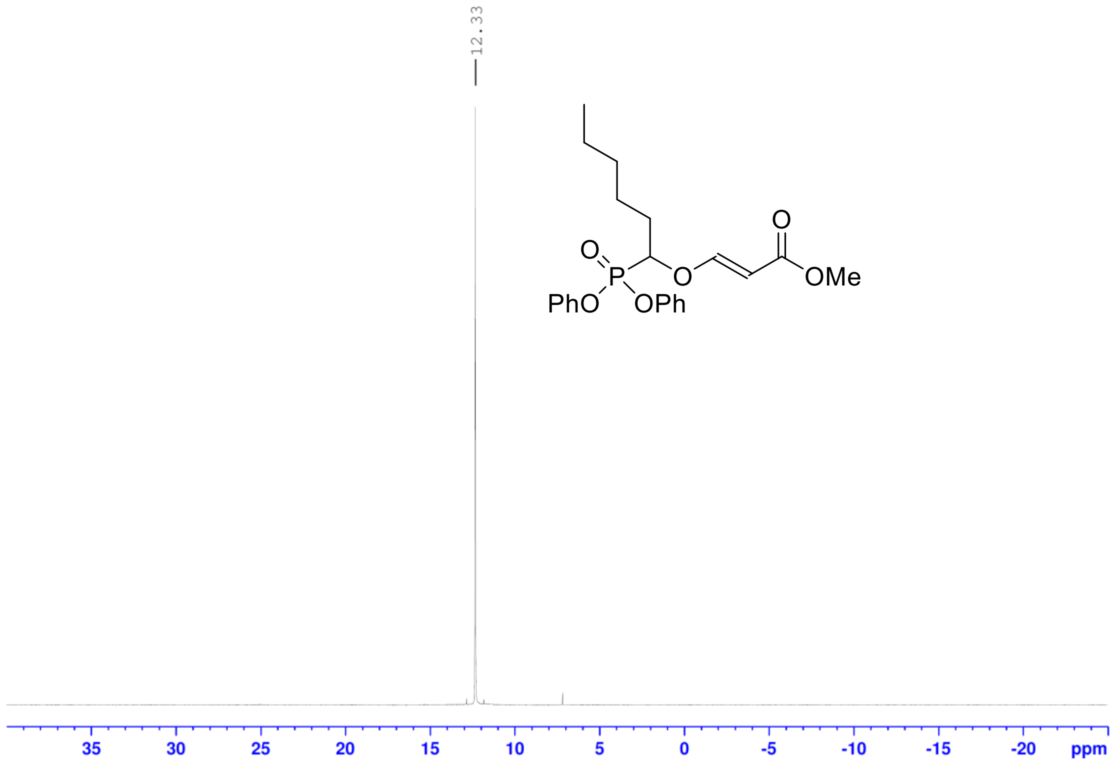

$^{31}P\{^{1}H\}$ NMR (162 MHz, $CDCl_3$) of compound **8ag**

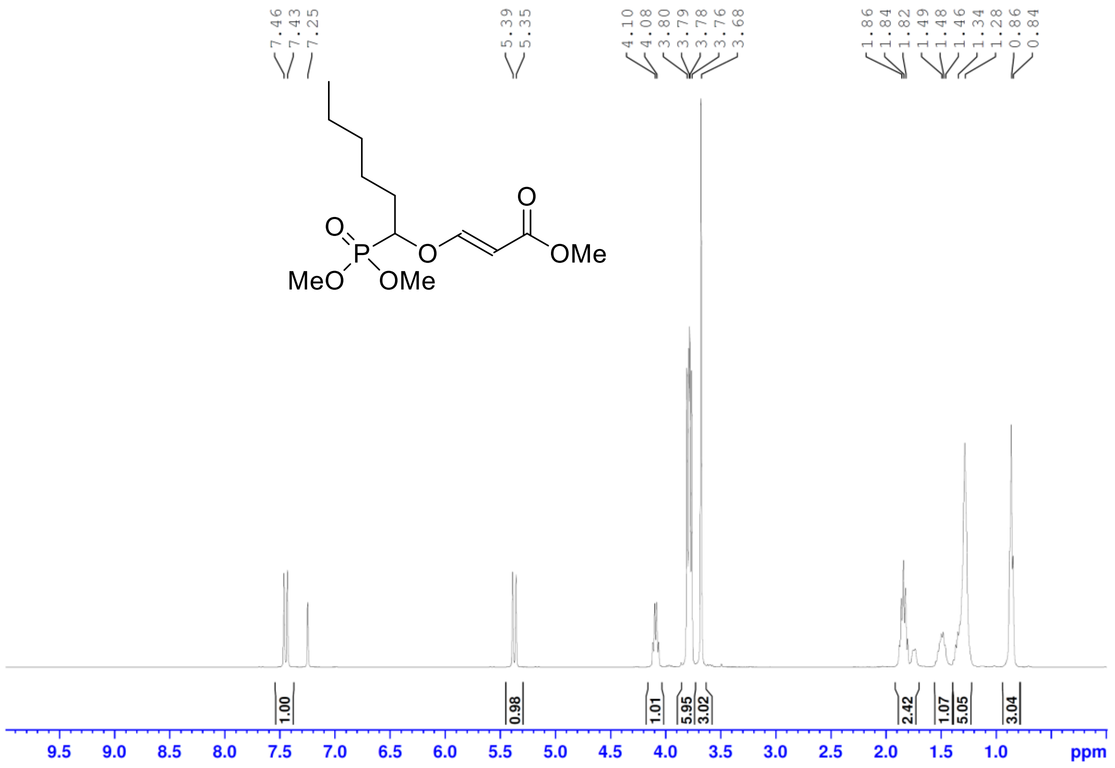

^{1}H NMR (400 MHz, $CDCl_3$) of compound **8bg**

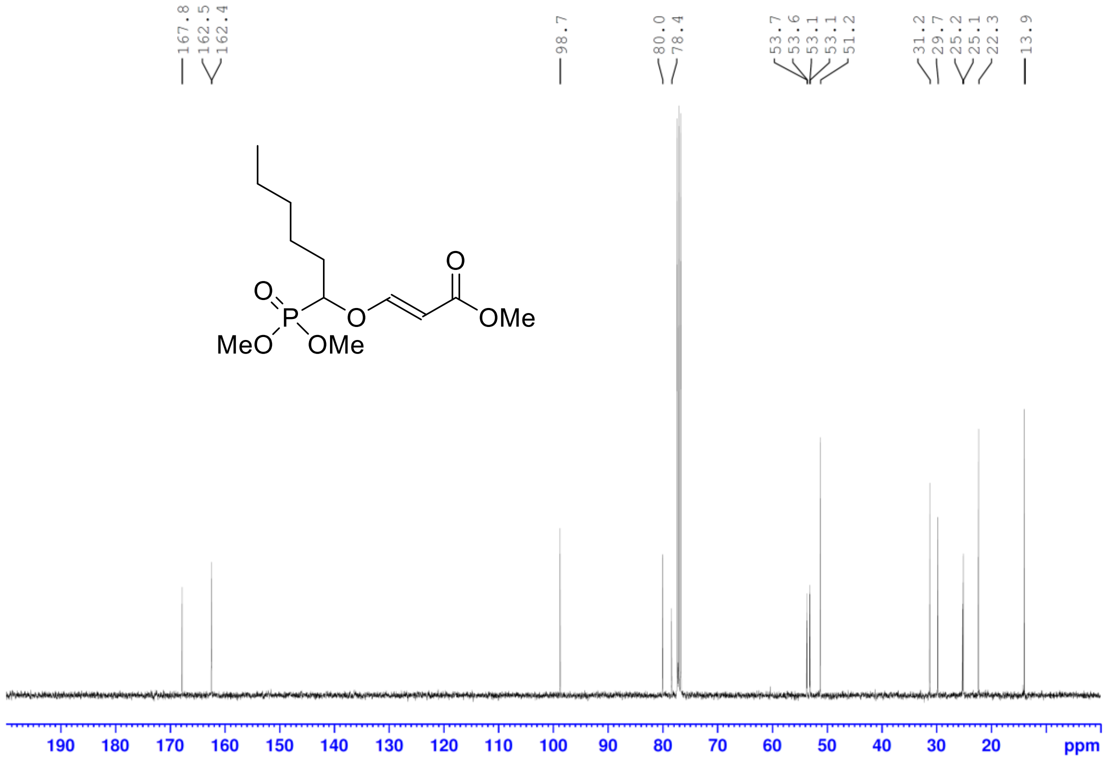

$^{13}C\{^{1}H\}$ NMR (100 MHz, $CDCl_3$) of compound **8bg**

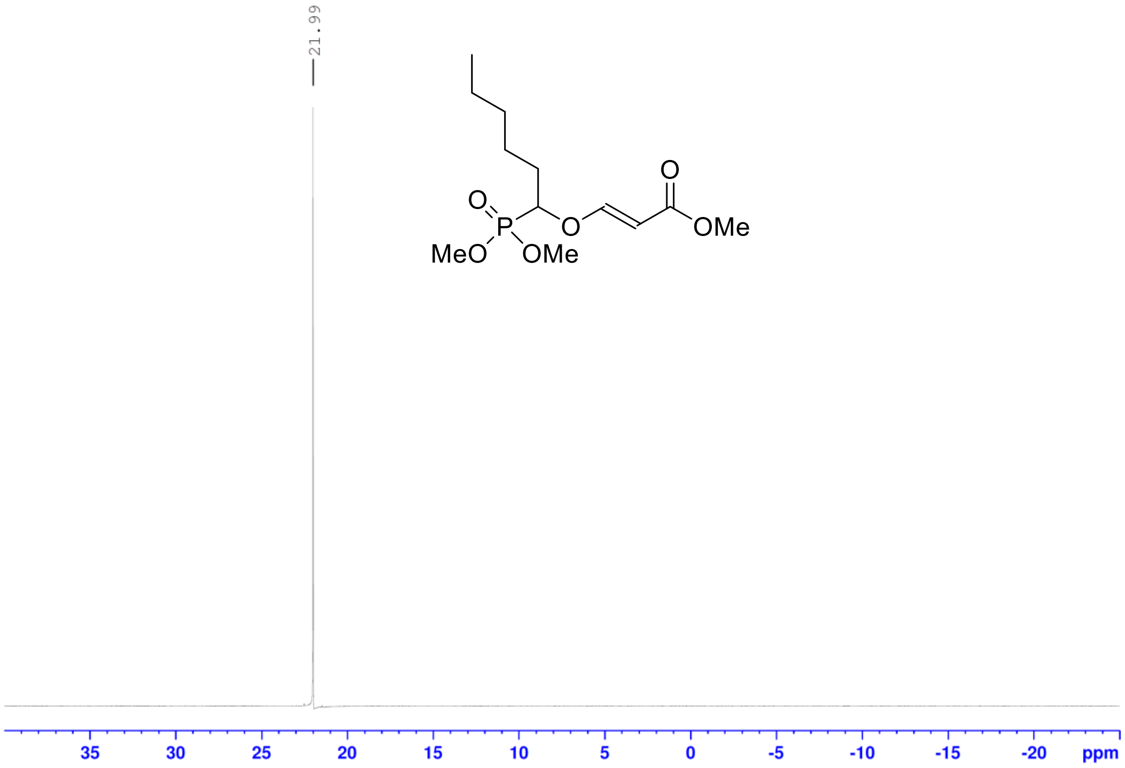

$^{31}P\{^{1}H\}$ NMR (162 MHz, $CDCl_3$) of compound **8bg**
